# Cell-free expression with a quartz crystal microbalance enables rapid, dynamic, and label-free characterization of membrane-interacting proteins

**DOI:** 10.1038/s42003-024-06690-9

**Published:** 2024-08-17

**Authors:** Aset Khakimzhan, Ziane Izri, Seth Thompson, Oleg Dmytrenko, Patrick Fischer, Chase Beisel, Vincent Noireaux

**Affiliations:** 1 School of Physics and Astronomy, University of Minnesota, Minneapolis, MN 55455 USA; 2 Helmholtz Institute for RNA-based Infection Research (HIRI), Helmholtz-Centre for Infection Research (HZI), 97080 Würzburg, Germany; 3 Medical Faculty, University of Würzburg, 97080 Würzburg, Germany

**Keywords:** Membrane biophysics, Membrane lipids

## Abstract

Integral and interacting membrane proteins (IIMPs) constitute a vast family of biomolecules that perform essential functions in all forms of life. However, characterizing their interactions with lipid bilayers remains limited due to challenges in purifying and reconstituting IIMPs in vitro or labeling IIMPs without disrupting their function in vivo. Here, we report cell-free transcription-translation in a quartz crystal microbalance with dissipation (TXTL-QCMD) to dynamically characterize interactions between diverse IIMPs and membranes without protein purification or labeling. As part of TXTL-QCMD, IIMPs are synthesized using cell-free transcription-translation (TXTL), and their interactions with supported lipid bilayers are measured using a quartz crystal microbalance with dissipation (QCMD). TXTL-QCMD reconstitutes known IIMP-membrane dependencies, including specific association with prokaryotic or eukaryotic membranes, and the multiple-IIMP dynamical pattern-forming association of the *E. coli* division-coordinating proteins MinCDE. Applying TXTL-QCMD to the recently discovered Zorya anti-phage system that is unamenable to labeling, we discovered that ZorA and ZorB integrate within the lipids found at the poles of bacteria while ZorE diffuses freely on the non-pole membrane. These efforts establish the potential of TXTL-QCMD to broadly characterize the large diversity of IIMPs.

## Abstract (web summary)

Quartz crystal microbalance combined with cellfree gene expression offers real-time, sensitive, and label-free measurements of interaction and integration of proteins with or into lipid membranes.

## Introduction

Lipid bilayers form the physical boundaries between the inner compartment of a living cell and the environment. To sense and interact with their surroundings, cells synthesize integral and interacting membrane proteins (IIMPs) that localize either into or at the surface of phospholipid membranes. IIMPs constitute a large family of biomolecules achieving broad cellular functions that interface cells with their milieu^1,2^. Lipid bilayers also serve as physical templates for IIMPs to organize cellular functions, comprising, for instance, the formation of dynamical patterns^3^ and anchoring cytoskeletons^4,5^. Characterizing IIMPs non-disruptively, such as the biochemistry and biophysics of their interactions with membranes, is often challenging to achieve in vivo as they usually require labeling with fluorescent reporters or affinity tags^6^. Conversely, the reconstitution of IIMPs in vitro provides easier access to their biochemical and biophysical characterization^7^. Yet, this approach necessitates difficult recombinant purification and reconstitution procedures that prevent rapid exploration of their properties^8^.

Cell-free transcription-translation (TXTL) simplifies and outpaces traditional recombinant approaches by enabling the scalable synthesis of proteins outside living cells, including IIMPs, in a matter of hours from plasmids or linear DNA^9–11^. Many TXTL reactions can be prepared concurrently, thus facilitating the rapid and parallel characterization of reaction products. To fold properly and not precipitate, IIMPs synthesized in TXTL require either the presence of non-natural substrates like surfactants or natural membranes made of phospholipids^12–15^, which is typically achieved by adding liposomes or nanodiscs to the TXTL reaction. Liposomes added to TXTL reactions are known to precipitate^16^. Consequently, the liposome approach to TXTL of IIMPs is limited and has been scarcely used. While nanodiscs offer a simple method to synthesize IIMPs in TXTL^16–18^, they are limited in lipid compositions^19^, reducing the scope of IIMP-lipids interactions that can be studied. IIMPs can also be synthesized in TXTL reactions encapsulated into liposomes, a system known as synthetic cells^20^. In these settings, too, disruptive fluorescent labelling is required to visualize the interaction with the membrane and the lipid composition of such liposomes is limited. Preparing TXTL-based synthetic cells with complex membrane compositions, such as *E. coli* phospholipids, is limited, as the efficacy of liposome production rapidly decreases with the complexity of the lipid composition. Thus, there is a lack of an approach that enables the sensitive and label-free characterization of IIMPs’ interactions with lipid membranes of arbitrarily complex membrane composition.

**Fig. 1:**
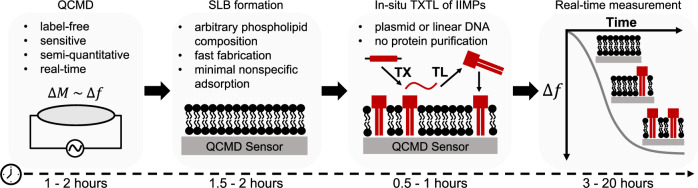
Workflow of the TXTL-QCMD experimental procedure. The TXTL-QCMD procedure allows non-disruptive semi-quantitative measurement of the interactions between in situ synthesized proteins and lipid bilayers. The first step of the procedure is the QCMD module setup, which takes ~2 hours. The second step of the experiment is the fabrication of the supported lipid bilayer (SLB). Using the solvent-assisted lipid bilayer formation method (SALB), SLBs of arbitrary phospholipid compositions are made in 2 hours. The third step is the preparation of the TXTL reaction, which involves mixing the TXTL components with a plasmid or a linear construct of interest. TXTL enables characterizing IIMPs that require co-translational integration. The last step is the incubation of the prepared TXTL reactions in contact with the SLB-sensor system. Typically, the reactions are incubated overnight; however, in some cases, as few as 3 hours are sufficient to obtain experimentally relevant data.

In this work we achieved rapid, sensitive, and label-free characterization of IIMPs’ interactions with supported lipid bilayers (SLBs) by combining the versatility of TXTL with the sensitivity of a quartz crystal microbalance with dissipation (QCMD)^21^ (Fig. 1). A QCMD is a tool to sensitively measure the added mass and the rates of molecular adsorption. This measurement is performed by continuously tracking the changes in the sensor’s resonant frequency, which, to a first approximation, is proportional to the mass deposited on the sensor^22^. We used the change in the resonant frequency of the QCMD to measure the interaction between IIMPs and a given phospholipid composition of the bilayer. The proof of concept of such an approach has been established for single IIMPs^23,24^, but the method has never been generalized to multiple and interacting IIMPs, membrane-dependent dynamical patterns, and complex phospholipid membrane compositions. This approach provides a high signal-to-noise ratio due to minimal nonspecific adsorption of the TXTL components onto SLBs. IIMPs, without any modifications such as the addition of fluorescent reporters, are dynamically synthesized on the SLBs inside the QCMD while monitoring the signal, which eliminates recombinant expression, purification, and reconstitution procedures. SLBs made of complex phospholipid compositions that are analogous to the ones found in living cells can be prepared onto QCMD sensors, circumventing the limitations of the other methods.

## Results

### TXTL reactions have minimal nonspecific adsorption on SLBs

The TXTL system uses the endogenous *E. coli* core RNA polymerase and sigma factor 70 present in the lysate as the sole primary transcription mechanism. This system does not contain any remaining live *E. coli* cells (Fig. S1). Genes are expressed either from plasmids or linear dsDNA, from *E. coli* promoters (e.g., P70a), or from the T7 promoter via a transcriptional activation cascade (Fig. S2), as previously described^25–27^.

**Fig. 2:**
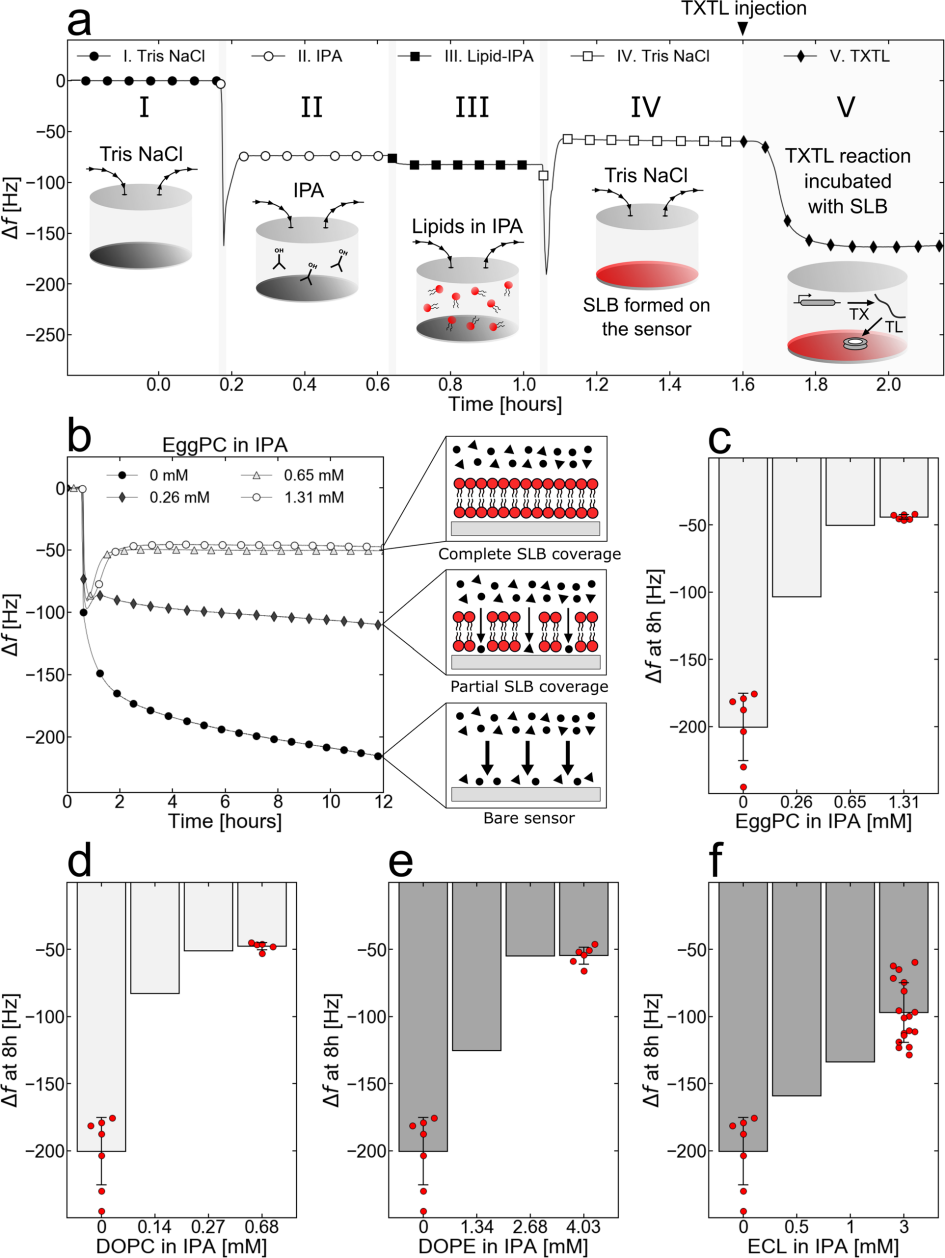
An optimal lipid-isopropanol concentration during SALB is critical for complete SLB coverage and minimal TXTL nonspecific adsorption. **a** Experimental steps of SALB formation (steps I–IV) followed by the nonspecific adsorption characterization with a TXTL reaction that only synthesizes the T7 RNA polymerase (step IV, plasmid P70a-*T7rnap* 0.15 nM). **b** The adsorption kinetics of a blank TXTL reaction (plasmid P70a-*T7rnap*, 0.15 nM) depend on the EggPC phospholipids concentration in IPA during step II. The cartoon frames on the right explain the different levels of nonspecific adsorption to the SLB-sensor system. **c** Changes in frequency after 8 hours of incubation (blank TXTL reaction with only P70a-*T7rnap*, 0.15 nM) as a function of phospholipids concentration in IPA step II for EggPC. **d**–**f** Same as **c**. for DOPC, DOPE, and *E. coli* lipids (ECL) total respectively. The bar level and error bars are the mean and standard deviation of at least *n* = 5 independent experiments.

The interaction with and integration of IIMPs into membranes depend on the membrane’s phospholipid composition^28^. Consequently, the diversity of SLBs that can be fabricated on the QCMD sensor determines the breadth of IIMPs that can be characterized, in particular IIMPs specific to the membranes of either bacteria or higher organisms. To make SLBs on the QCMD sensor, we used the solvent-assisted lipid bilayer formation method (SALB)^29–31^, as it is fast and consistent (Fig. 2a). Our model eukaryotic membrane is made of EggPC, a mixture of PC phospholipids extracted from chicken eggs, with various aliphatic chain lengths. Our model prokaryotic cell membrane is made of *E. coli* lipids (ECL) from *E. coli* cells. ECL is composed of mainly PE (phosphatidylethanolamine, ~75% mol), PG (phosphatidylglycerol, ~20% mol), and CL (cardiolipin, ~5% mol)^32^. We also studied the interactions of IIMPs with other SLBs made from mixtures of pure phospholipids. In these cases, the SLBs were made using the most abundant phospholipid found in EggPC (DOPC) or ECL (DOPE). A TXTL reaction contains a broad variety of biological molecules in large concentrations; it is thus important to make sure that they do not produce large nonspecific adsorption on the sensor-SLB system in the absence of IIMP synthesis. We devised a procedure so that SLBs cover the whole sensor and have minimal nonspecific TXTL adsorption.

The SLBs were made in four steps (Fig. 2a). In step I, the QCMD sensor is washed with a Tris NaCl buffer, which is replaced in step II by an isopropyl alcohol (IPA) solution. In step III, phospholipids dissolved in IPA are introduced onto the sensor. The spontaneous SLB formation occurs in step IV, as the IPA with the dissolved phospholipids is gradually replaced with a Tris NaCl buffer^31^. The difference between the stabilized resonance frequency (∆*f* = 0) from steps I to IV is proportional to the mass of the SLB on the sensor^33,34^. When no phospholipids are added to the IPA during step III, the difference is zero (Fig. S3). In all the subsequent data presented, the resonance frequency at the end of step IV, before adding the TXTL reaction onto the QCMD sensor, was taken as the reference and set to zero. A short-lived dip in frequency is systematically produced at the beginning of each step. This is due to the introduction of a different solution into the QCMD modules (Fig. 2a). This short-lived dip in frequency, also present when a TXTL reaction is introduced into the QCMD chambers, is irrelevant to the measurement of the interaction between the IIMPs and the SLBs.

To determine whether an SLB fully covers the sensor, is stable, and does not allow nonspecific TXTL adsorption, a blank TXTL reaction (with only the plasmid P70a-*T7rnap*, 0.15 nM) was incubated into the QCMD module in contact with the SLB-sensor system. SLBs were made with different concentrations of phospholipids to determine at which phospholipid concentration nonspecific TXTL adsorption is not observed. This assay was carried out for each SLB tested in this work. In the case of an EggPC membrane, the full coverage of the QCMD sensor is achieved at a phospholipid concentration at or above 0.65 mM (0.5 mg/mL) in IPA, as observed by the flat frequency shifts (Fig. 2b). Below a concentration of 0.65 mM, an EggPC membrane shows nonspecific TXTL adsorption as observed by the decrease in ∆f for lower phospholipid concentration (Fig. 2c). The minimal phospholipid concentration to get SLBs that fully cover the QCMD sensor was first determined for our four base SLBs, namely EggPC, DOPC, DOPE, and ECL SLBs (Fig. 2c–f, Fig. S4, Table S1). Interestingly, the concentrations needed for full coverage varied depending on the type of lipids, presumably due to the charge of the lipid head. Lipids with PC headgroups formed SLBs at smaller concentrations compared to the lipids with the more polar PE headgroups. ECL requires an even larger concentration and is composed of PE lipids along with more polar and charged lipids with PG and CL headgroups.

We found that the four major phospholipid compositions used in this work can be used reproducibly for QCMD-TXTL experiments without nonspecific adsorption from the TXTL components (Table S1). Achieving full coverage of the QCMD sensor with lipid bilayers is a striking result that shows the natural resistance of phospholipid membranes to nonspecific adsorption of a complex physiological solution like a TXTL reaction composed of 10 mg/mL of proteins, tRNAs, and rRNAs, ~250–300 mM salts and many other chemicals^27,35^. The affinity of TXTL to the different SLBs can be estimated by comparing the frequency shifts between the pre-TXTL (step IV) and post-TXTL Tris NaCl flushes (Fig. S5a). One reason for the affinity of TXTL to the DOPE and ECL SLB-sensor systems is larger than to DOPC and EggPC SLBs is because the proteins in the lysate bind to bilayers composed of their naturally occurring lipids (PE, PG, CL) (Fig. S5b).

### Cell-free synthesized IIMPs interact specifically with their natural membranes

**Fig. 3:**
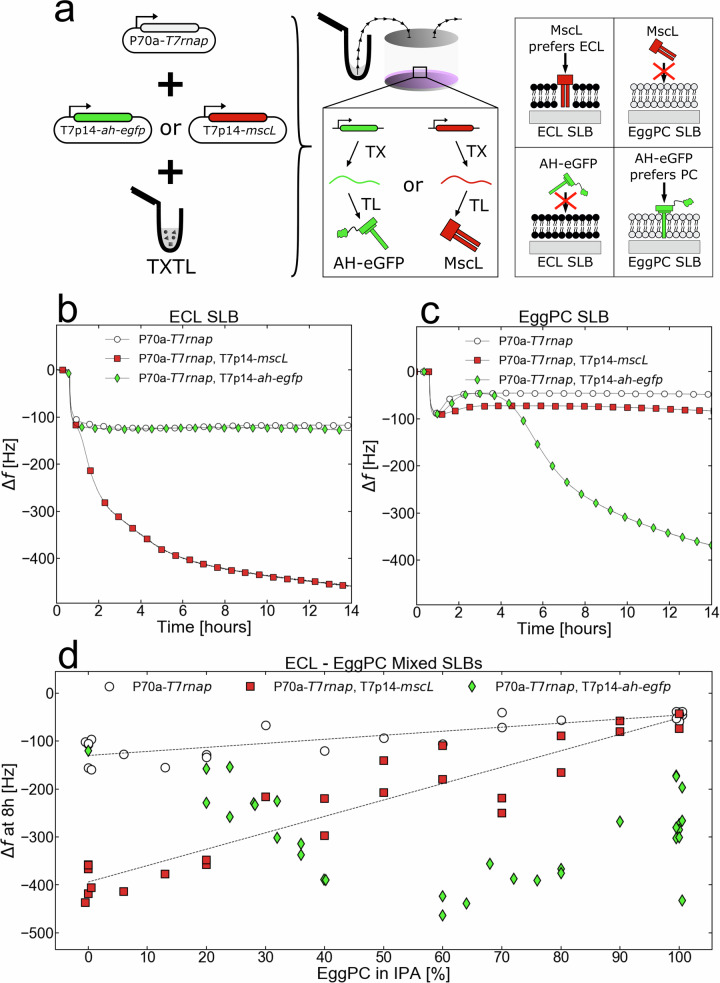
In situ cell-free synthesized MscL and AH-eGFP exhibit their natural phospholipid affinities in ‘pure’ and ‘mixed’ SLBs. **a** MscL or AH-eGFP is synthesized into the QCMD module on top of either a PC-rich EggPC SLB or a PE-rich *E. coli* lipids (ECL) SLB. The rightmost cartoon demonstrates the expected protein-lipid preferences of both proteins. **b**, **c** Adsorption kinetics of blank (P70a-*T7rnap*, 0.15 nM), AH-eGFP (P70a-*T7rnap*, 0.15 nM, T7p14-*ah-egfp*, 5 nM), and MscL (P70a-*T7rnap*, 0.15 nM, T7p14-*mscL*, 5 nM) synthesizing TXTL reactions in contact with an ECL SLB and an EggPC SLB respectively. **d** The frequency changes after 8 hours of incubating either blank (P70a-*T7rnap*, 0.15 nM), AH-eGFP (P70a-*T7rnap*, 0.15 nM, T7p14-*ah-egfp*, 5 nM), or MscL (P70a-*T7rnap*, 0.15 nM, T7p14-*mscL*, 5 nM) TXTL reactions in ECL–EggPC Mixed SLBs. The x-axis percentage indicates the concentration of EggPC (and ECL) relative to the working concentration from Table S1. For example, 40% EggPC means that the Lipid-IPA mix contains 0.52 mM of EggPC phospholipids (40% of 1.3 mM) and 1.8 mM of ECL phospholipids (60% of 3 mM). All of the performed replicates are shown in the figure as individual data points.

To test the QCMD-TXTL approach to membrane protein-lipid interaction specificities, we chose two proteins known to preferentially reside in different natural membranes. We performed a set of TXTL reactions that synthesize either the pore-forming protein Alpha-Hemolysin (AH) from *S. aureus*, known to target mammalian cells rich in PC^36^, and the large-conductance mechanosensitive channel MscL from *E. coli* in membranes (ECL) mostly composed of PE, PG, and CL. To quantify the amount of protein produced in the QCMD chamber, we used a fusion AH-eGFP proven to be functional in TXTL because it is produced as a soluble protein and thus easy to quantify^37^. Both genes were cloned under the T7p14 promoter and expressed via the T7 transcriptional activation cascade (T7p14-*ah-egfp*, T7p14-*mscL*) (Fig. 3a). AH assembles into a heptameric pore^38^, while MscL assembles into a pentamer channel^39^. We observed a net frequency drop relative to the blank only when MscL was synthesized onto the ECL SLB. Conversely, we observed a net frequency drop only when AH-eGFP was synthesized onto the EggPC SLB (Fig. 3b, c). Replicates of these experiments confirmed these results (Figs. S6, S7). The same protein-lipid specificities were observed with DOPE (interaction with AH-eGFP, no interaction with MscL) and DOPC (no interaction with AH-eGFP, interaction with MscL) SLBs (Fig. S8). A delay in frequency shift was observed for AH, presumably because it must reach a critical concentration before interacting with the membrane^40^. MscL pre-synthesized in TXTL reactions without membranes did not produce a frequency change (Fig. S9), which is expected because its integration is coupled to its translation. Co-translational integration also indicates that MscL most likely first integrates into bilayers as a monomer and afterwards assembles in the bilayer into the pentameric MscL. Unlike MscL, AH is a soluble pore-forming toxin that can be pre-synthesized 24 hours before being added onto the QCMD chip and still integrates into the PC SLB (Fig. S10), without a delay as it is introduced into the QCMD module at a large concentration. The concentration of IIMP produced in the QCMD chamber of volume 40 µL was estimated with AH-eGFP to be 1–2 μM (Fig. S11). We found this concentration range relevant as it corresponds to the average protein concentration in *E. coli*^41^. Using MscL, we estimated the resolution of the mass added to the SLB to be on the order of 1 ng, which is a hundred times more sensitive than a standard protein gel (Fig. S12). This estimation also shows that about twenty times more proteins are synthesized in the 40 µL-QCMD chambers with respect to the membrane’s maximum binding capacity.

When proteins are inserted into an SLB fully covering the QCMD sensor, the possibilities are that lipids are expelled from the SLB, the surface area per lipid decreases, or lipid protrusions form. Previous studies done by AFM have not shown the formation of lipid protrusions^23^. To determine if some lipids are expelled from the SLB during the incubation of either a blank TXTL reaction or an IIMP-synthesizing reaction, in this case, an MscL-synthesizing TXTL reaction, we added a fluorescent lipid (Rhodamine-PE, Rhod-PE). After 12 h of incubation, we dissolved the SLBs in IPA and measured the fluorescence. First, adding 3% of Rhod-PE to the SLB did not affect the integration of MscL into the DOPE SLBs (Fig. S13a). With that confirmed, we fabricated a DOPE + Rhod-PE SLB on the sensor and incubated it with four different conditions: a TXTL feeding solution (TXTL reaction without the lysate), a blank TXTL reaction (0.15 nM P70a-*T7rnap*), an MscL TXTL reaction (0.15 nM P70a-*T7rnap*, 5 nM T7p14-*mscL*), and 1% SDS (Fig. S13b). After 12 h of incubation, we dissolved the SLBs of each condition with IPA and measured the fluorescence intensity. It appears that neither the blank TXTL reaction nor the MscL TXTL reaction demonstrate an effect of lipid expulsion larger than the 8% uncertainty of the measurements (Fig. S13c). This either indicates that MscL occupies a relatively small percentage of the SLB, such that the expulsion of lipids would be unnoticeable or that the SLB can compact to accommodate the MscL channels without being expelled. The compaction is indeed possible as demonstrated in experiments with model lipid bilayers^42^, with the area per lipid contracting by almost 30% in some cases.

To determine whether IIMPs specific to different lipid kingdoms can coexist in hybrid membranes, such as the one that could be developed in engineered biochemical systems^43^, we devised hybrid SLBs made of EggPC and ECL. We measured the shift in frequency Δf of blank TXTL reactions (plasmid P70a-*T7rnap* only) and of TXTL reactions expressing *ah-egfp* and *mscL* (Fig. 3d, Fig. S14). The shift in frequency Δf was measured after eight hours of incubation, taken as an amount of time sufficient for the TXTL reaction and interaction with the SLB to reach equilibrium. We first established that at any ratio of phospholipids, the hybrid membrane formed remains devoid of nonspecific adsorption. In contact with the blank TXTL reaction, at any EggPC ratio, the frequency shift stabilized within 2 hours of incubation (Fig. S14a). We also observed a slight linear increase in the Δf with the increase of the EggPC ratio, which accounts for slight nonspecific interactions of the TXTL reaction with *E. coli* SLBs. For AH-eGFP, we observed a sigmoidal decrease of Δf as the EggPC ratio increased, whereas for MscL Δf increased linearly with the increase of the EggPC ratio, consistent with the respective specificity of AH-eGFP and MscL integration into EggPC and ECL. Considering the cooperativity of the assembly of multimeric complexes in the plasma membrane of bacteria^44^, the sigmoidal dependence for AH-eGFP is expected^45^, while the linear dependence observed for MscL is surprising. When the MscL TXTL reaction was incubated onto DOPE-DOPC hybrid SLBs, MscL exhibited a sigmoidal increase with the increase of the DOPC ratio (Fig. S15). These experiments underline the existence of different self-assembly and integration mechanisms, depending on the presence of lipids, such as DOPG or CL, present in ECL but absent in DOPE^46–48^.

### SLBs’ lipid compositions can be broadly tuned

**Fig. 4:**
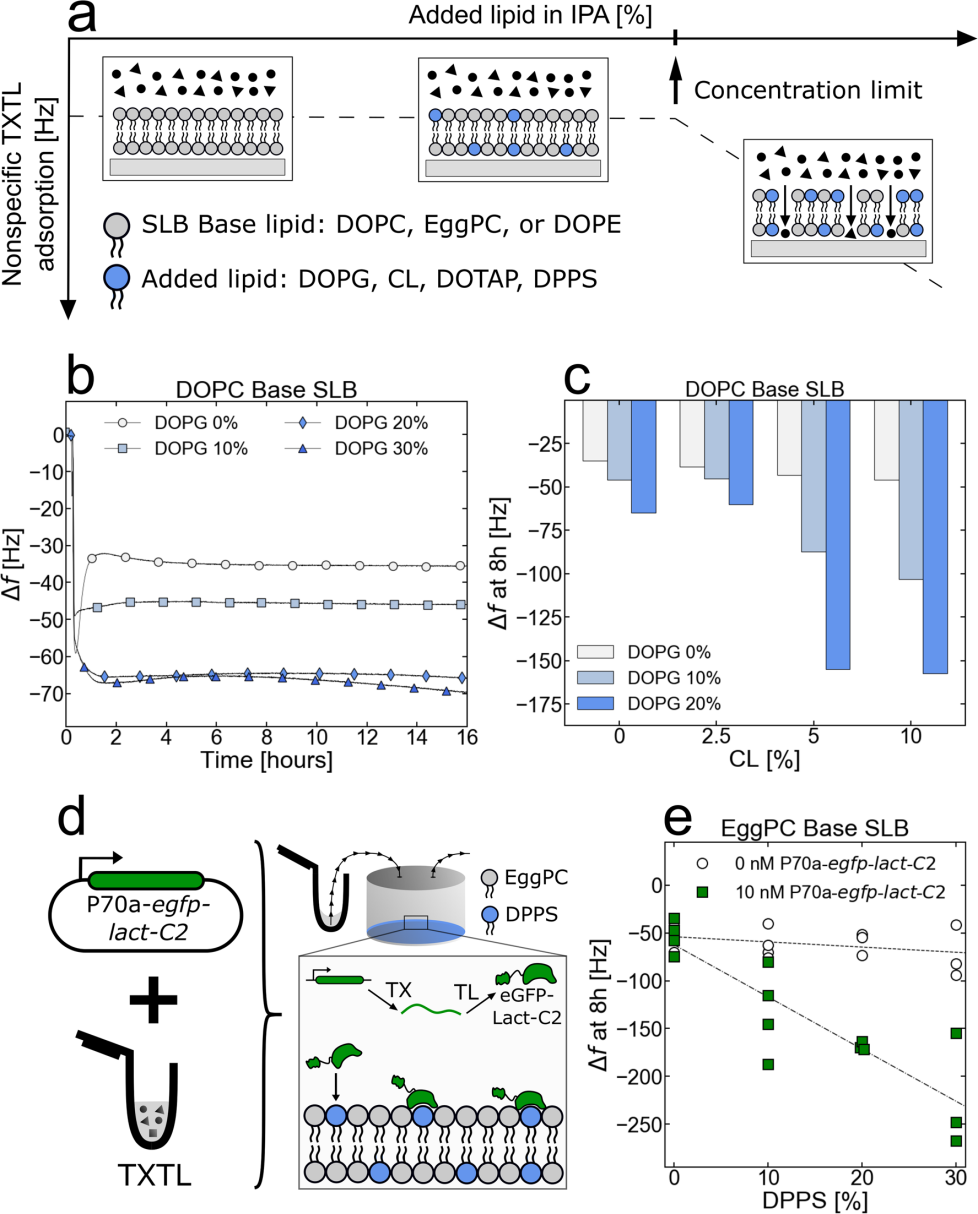
Phospholipids that cannot form stable SLBs in normal TXTL conditions can be added to ‘base lipid’ SLBs such as DOPC, EggPC, and DOPE SLBs. **a** Graphical illustration of the ‘added lipid’ concentration limit. Although phospholipids with various headgroups can be added to base SLBs, there is an observed limit to the added lipid concentration in the phospholipid-IPA mix before the SLB-sensor system incurs nonspecific adsorption from a blank TXTL reaction. **b** Adsorption kinetics of a blank TXTL reaction (P70a-*T7rnap*, 0.15 nM) in contact with DOPC/DOPG SLBs of different ratios. **c** The frequency change after 8 hours of a blank TXTL reaction (P70a-*T7rnap*, 0.15 nM) incubating over a DOPC Base SLB as a function of added CL and DOPG. **d** Cell-free synthesis of eGFP-Lact-C2 (plasmid P70a-*egfp-lact-C2*, 5 nM) into the QCMD module. The in situ synthesized eGFP-Lact-C2 interacts with the DPPS phospholipids. **e** The frequency change after 8 h of either a blank (no DNA added) or eGFP-Lact-C2 (P70a-*egfp-lact-C2*, 5 nM) TXTL reaction being in contact with EggPC/DPPS SLBs fabricated with varying SALB DPPS concentrations. All of the collected experiments are shown in this plot as individual data points.

Although PC (eukaryotes) and PE (bacteria) are usually the predominant phospholipids in natural cell membranes^49–51^, other headgroups are similarly essential for the function of IIMPs^28^. For example, some IIMPs only anchor to specific phospholipid heads other than PE or PC^28^, while other IIMPs require the presence of anionic phospholipids to bind^52^. While making SLBs from a mixture of different phospholipids is technically feasible with the SALB method (Fig. 4a), one must determine the degree of nonspecific adsorption of TXTL on the SLBs. We measured the concentrations of DOPG, CL, and DPPS (1,2-dipalmitoylphospho-L-serine) into three of our base SLBs (DOPC, EggPC, and DOPE) for which nonspecific TXTL adsorption is absent (Fig. 4a). We used the PS sensing protein LactC2 to show that one can adjust quantitatively the amount of an added lipid to an SLB.

The added lipid percentage of the lipid-IPA mixture is defined as the ratio of the molarity of the added lipid over that of the base lipid. Using a blank TXTL reaction (plasmid P70a-*T7rnap*, 0.15 nM), we found that adding DOPG up to 30% to either DOPC or DOPE SLBs did not produce nonspecific TXTL adsorption (frequency drifts of <1 Hz/hour) (Fig. 4b and Fig. S16). We also found that up to 10% of CL added to DOPE SLBs (Fig. S17) and in DOPC SLBs (Fig. S18a) did not produce nonspecific adsorption. The conditions for which nonspecific TXTL adsorption is not observed when both DOPG and CL are added to a DOPC SLB were also determined (Fig. 4c, Fig. S18b, c). As the concentration of CL in the lipid-IPA mix is increased, the amount of DOPG must be decreased to prevent nonspecific TXTL adsorption. The limit concentration of each of the tested added lipids was determined by following the same procedure (Table S1).

LactC2 is a mammalian signaling protein that binds specifically to PS phospholipids^53–55^. When eGFP-LactC2 was synthesized (plasmid P70a-*egfp-lactc2*, 5 nM) on DPPS-EggPC SLBs, the shift in frequency Δf was linearly proportional to the amount of DPPS added to the IPA-EggPC-DPPS solution used to form the SLB (Fig. 4d, e, Fig. S19). Because more eGFP-LactC2 proteins were synthesized than DPPS sites in the SLB available for binding (Fig. S19c), this experiment shows that the amount of DPPS in an EggPC base SLB corresponds to the proportion of DPPS used to make the SLB.

### The *E. coli* Min system produces oscillations on SLBs

To determine whether the TXTL-QCMD approach enables the sensing of membrane-based dynamical patterns on and greater than mesoscopic scales, we assayed the *E. coli* Min system that uses the inner membrane to position the division machinery via pole-to-pole dynamical oscillations^56^. In vitro, the three Min proteins MinD, MinE, and MinC create a myriad of oscillatory patterns in vesicles^57–60^ and on SLBs^61–64^. These patterns are the result of the reaction-diffusion dynamics of MinD and MinE. Upon ATP binding, MinD forms a dimer that binds to the inner bacterial membrane, which accelerates MinD recruitment at the membrane. MinE, which forms a dimer in the cytoplasm, binds to the MinD dimer at the membrane and catalyzes the hydrolysis of the ATP bound to MinD, effectively releasing MinD from the membrane^65–68^. While not essential for oscillations^57^, MinC is known to bind to MinD and ‘ride’ the MinD waves^69^. Visualizing MinCDE dynamical patterns requires fluorescent tagging of the proteins, which can interfere with the function of proteins^70^.

**Fig. 5:**
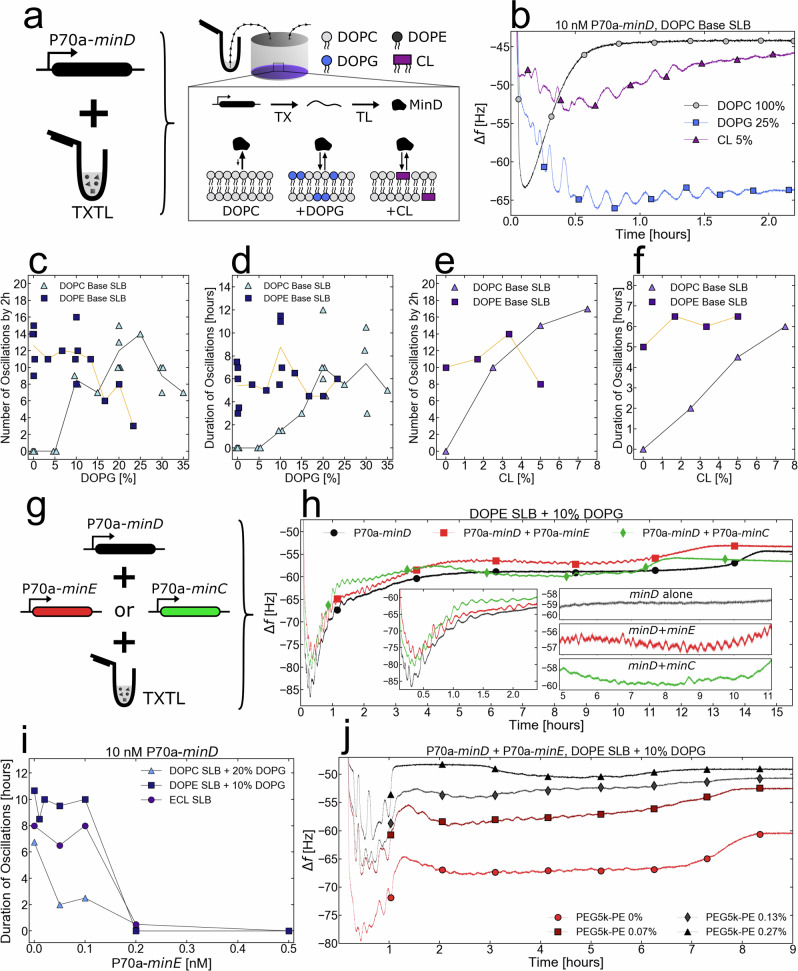
In situ TXTL synthesis of Min proteins induces coherent long-range mass oscillations across the SLB-Sensor system. **a** Cell-free synthesis of MinD (linear P70a-*minD*) into the QCMD modules on top of SLBs. The cartoon shows a very simplified model of the synthesized MinD binding kinetics when the reaction is in contact with either a pure DOPC SLB, a DOPC/DOPG SLB, or a DOPC/CL SLB. **b** Adsorption kinetics of MinD with either a pure DOPC SLB, a DOPC/DOPG SLB, or a DOPC/CL SLB. The curves are labeled with a few symbols, the lines are experimental points. **c** Number of oscillation peaks within the first 2 hours as a function of the relative DOPG concentration during SALB for a DOPE and DOPC Base SLB. **d** Duration of oscillations as a function of the relative DOPG concentration during SALB for a DOPE and DOPC Base SLB. **e** Number of oscillation peaks within the first 2 hours as a function of the relative CL concentration during SALB for a DOPE and DOPC Base SLB. **f** Duration of oscillations as a function of the relative CL concentration during SALB for a DOPE and DOPC Base SLB. **g** Cell-free synthesis of MinD with either MinE (linear P70a-*minE*) or MinC (linear P70a-*minC*) into the QCMD modules on top of SLBs. **h** Adsorption kinetics of MinD (P70a-*minD*, 10 nM), MinDE (P70a-*minD*, 10 nM and P70a-*minE*, 0.1 nM), and MinCD (P70a-*minD*, 10 nM and P70a-*minC*, 5 nM), on a 10% mol ratio DOPG/DOPE SLB. The left inlet shows a close-up of the oscillations during the first 2.4 hours. The right inlet shows a close-up of the oscillations between *t* = 5 hours and *t* = 11 hours. The curves are labeled with a few symbols, the lines are experimental points. **i** Duration of oscillations as a function of P70a-*minE* concentration in the TXTL reaction for a DOPG/DOPE SLB, a DOPG/DOPC SLB, and an *E. coli* lipids (ECL) SLBs. **j** Adsorption kinetics of a MinDE (P70a-*minD*, 10 nM and P70a-*minE*, 0.1 nM) on a 10% mol ratio DOPG/DOPE SLB with a range of PEG5k-PE added during SALB. The curves are labeled with a few symbols, the lines are experimental points. Each measurement is represented as an individual data point.

First, we performed a set of experiments in which we only synthesized MinD onto the SLBs (10 nM linear P70a-*minD*). Previous studies have shown that anionic phospholipids, such as PG and CL, facilitate MinD interaction with the membrane^71^. We added either DOPG or CL to a DOPC SLB (Fig. 5a). No oscillations were observed on pure DOPC SLBs, while on the DOPG-DOPC and CL-DOPC SLBs the synthesized MinD-induced oscillations with 5.5 min and 6.7 min mean periods respectively in the first two hours (Fig. 5b). While MinC is not necessary for the Min oscillations^57^, it has been reported that both MinD and MinE are required to form spatiotemporal patterns^3^. Proteomics of our TXTL system showed trace amounts of MinD, whereas MinE and MinC were not detected^35^. Blank TXTL reactions never produced spontaneous oscillations on SLBs. The synthesized MinD oscillations exhibited two different modes: an unstable and large-amplitude mode in the first 2 hours of incubation and a stable small-amplitude mode for the rest of the reaction (Fig. S20a–c). In both modes, the period was around 10 minutes, longer than the 1 minute periods observed in confined systems such as *E. coli*  bacteria^56^ or micron-sized microwells^72^. The oscillations stopped with an increase in Δf of 2–10 Hz depending on the replicate, which we interpret as the final unbinding of MinD from the SLB after the ATP that fueled the oscillations has been depleted (Fig. S20d). Next, we determined the ranges of concentrations of DOPG and CL that can be added to either a pure DOPC (not found in *E. coli* membranes) or pure DOPE (major phospholipid in *E. coli* membranes) SLB for which the Min oscillations were observed when MinD (10 nM linear P70a-*minD*) only was synthesized in the TXTL reaction. To track the effect of DOPG concentrations, we counted the number of oscillations during the first mode (larger oscillations), and the duration of the oscillations during the second mode (smaller oscillations). In this series of experiments, the behavior of the initial mode was strongly dependent on the charge of the SLB, with both the DOPE and DOPC base SLBs exhibiting optimal DOPG molar ratios of 5–10% and 20–25%, respectively (Fig. 5c, Figs. S21, S22). The difference in the optimal DOPG percentage can be partially attributed to the difference in the availability of the positive charge in the zwitterionic headgroups of DOPE and DOPC. When the reaction duration was measured, we noticed that the DOPE SLBs were significantly more robust to variations in the DOPG concentration compared to the DOPC SLB (Fig. 5d, Figs. S23, S24). Similarly, as we increased the concentration of CL in the SLB, we observed an increase in the number of oscillations in the first 2 hours alongside the increase of oscillation lifetime for the DOPC SLB cases (Fig. 5e, f). Changes in the CL concentration in DOPE base SLB did not significantly affect the behavior of the pattern-forming reactions. Overall, the DOPE base SLB provided more robustness to lipid composition changes compared to the DOPC base SLBs for observing oscillations. When DOTAP, an artificial cationic lipid, was added to DOPC, DOPG-DOPC, DOPE, and DOPG-DOPE SLBs, the oscillations became weaker as the concentration of DOTAP increased (Figs. S25–S27). This shows that Min patterns are sensitive to the charges present in the SLB, with negative charges helping their formation, while positive charges inhibit oscillations. This result can be considered together with the fact that the *E. coli* inner membrane has a highly nonuniform density of PG and CL, with larger densities localized at the poles, especially for CL^73–75^. Min patterns in *E. coli* must be able to maintain oscillatory order in these locally different phospholipid compositions simultaneously, thus requiring the patterns to be robust to anionic phospholipid variation.

We co-synthesized either MinC or MinE with MinD in the presence of a DOPE-based SLB with 10% DOPG (Fig. 5g) to determine the effect of each on the MinD oscillations. In the presence of MinC (2 nM linear P70a-*minC*), the period of the oscillations remained unchanged while their amplitude increased. Adding MinE in some of the reactions produced a doubling of the mass oscillation period (Fig. 5h), which is likely an oscillatory regime in which MinE is recruited by MinD on some regular distance as observed in previous MinDE SLB experiments^62^. This regime did not occur in all the MinDE experiments that we tested but revealed patterns that can exist on the sensor when both *minD* and *minE* were expressed at optimal concentrations. To determine the concentrations of P70a-*minE* for which the pattern-forming reaction is the longest, we performed a series of experiments in which the *minD* expressing DNA concentration was fixed (10 nM linear P70a-*minD*), while the concentration of the *minE-*expressing DNA was varied (linear P70a-*minE*). These *minE* DNA ranges were performed for the optimal DOPG concentrations of both the DOPE (10% DOPG) (Fig. S28) and DOPC (20% DOPG) (Fig. S29) base SLBs and for the pure ECL SLB (Fig. S30). The DOPG/DOPC SLB demonstrated a decay in the reaction time as the MinE-expressing DNA was increased in concentration. DOPG/DOPE and ECL SLBs demonstrated robustness to the MinE variation up to 0.1 nM of added P70a-*minE* and no prolonged oscillations with P70a-*minE* concentrations greater than 0.1 nM (Fig. 5i). These results support that PE phospholipids play an integral role in the robustness of Min patterns, and that PG is not the only critical headgroup for Min oscillations.

Natural membranes are crowded with many membrane proteins. To address the effect that molecular crowding has on the Min patterns, we added PEG5000-PE (PE phospholipid with a PEG moiety of molecular mass 5000 g/mol) at various concentrations into a DOPE SLB with 10% DOPG. We expressed both *minD* (10 nM linear P70a-*minD*) and *minE* (0.1 nM linear P70a-*minE*) at optimal concentrations in the QCMD module. As we increased the amount of PEG5000-PE in the SLB, we observed an increase in the amplitude of the early oscillations (Fig. 5j). Membrane molecular crowding accelerates the binding of MinD to the SLB, which can be reasoned by the fact molecular crowding facilitates the formation of MinD multimers^76–79^. Molecular crowding decreased the total reaction duration and, in the 0.27% PEG5000-PE case, caused a 2-hour long transient disappearance of oscillations after the large-amplitude oscillations in the first hour. It is important to note that producing mass oscillations across the entire sensor requires that the MinD proteins collectively bind and unbind from the SLB in-phase and the transient disappearance of the oscillations might indicate that the Min patterns are exhibiting energetically costly chaotic dynamics^64,80,81^ during which the MinDE system is assessing a more ordered state^82,83^. This result emphasizes the strength of a tool (such as QCMD) that measures the total mass of a pattern-forming system. With the amplitude of the oscillations and the duration of the reactions, we could simultaneously assess the relative correlation lengths and the energy efficiency of the pattern-forming system as a function of the randomly distributed SLB impurities (PEG) and morphogen concentrations. In future studies, it would be particularly interesting to see how adding impurities of controlled size and position on the sensor affects the energetic efficiency and coherence of MinCDE patterns or other membrane-associated dynamical pattern-forming systems.

### Type II Zorya antiphage defense system is membrane associated

**Fig. 6:**
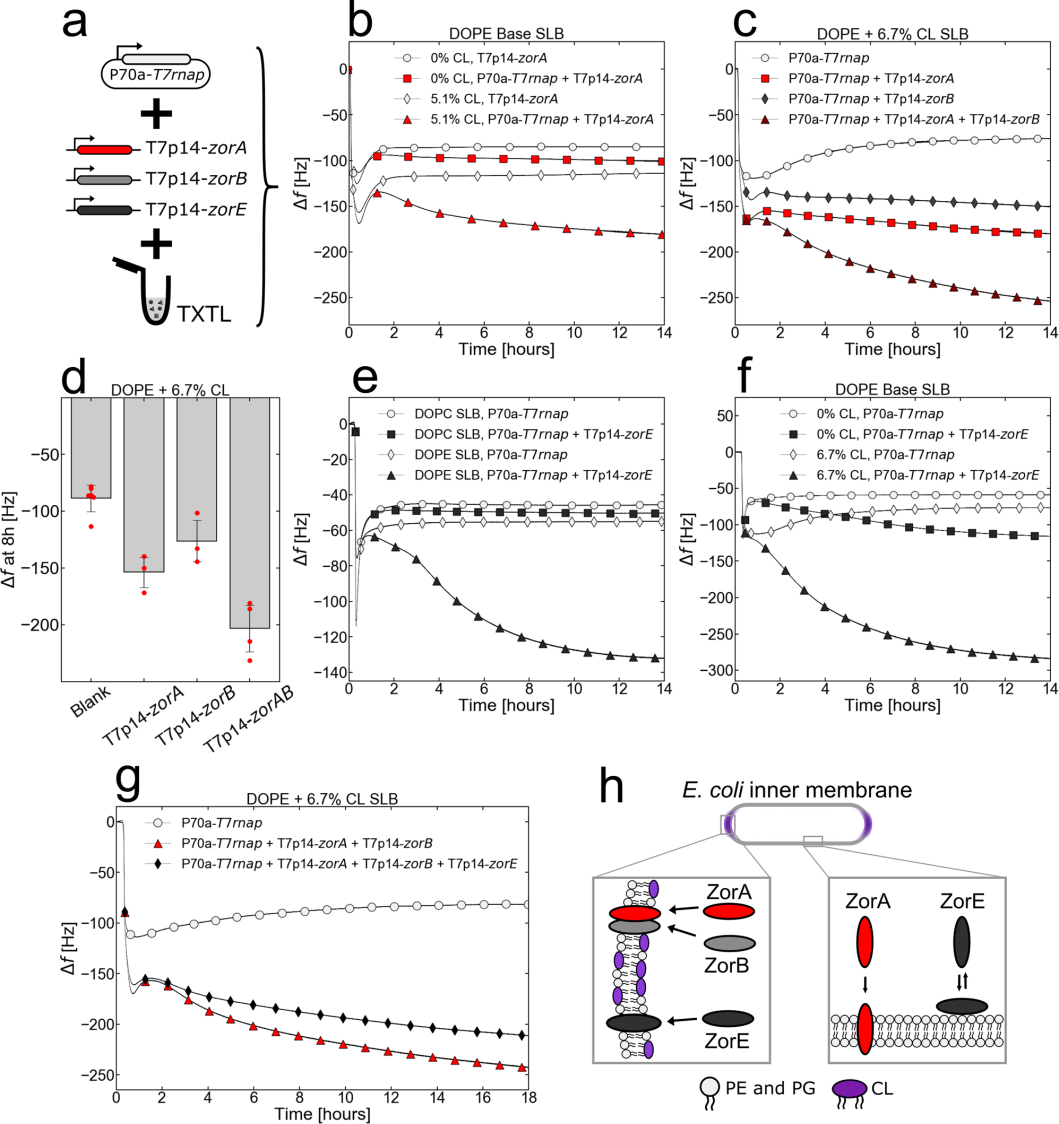
Type II Zorya defense proteins interact with cardiolipin-rich lipid membranes. **a** ZorA, ZorB, and ZorE were synthesized via the T7 transcriptional activation cascade (plasmid P70a-*T7rnap*, 0.15 nM, linear T7p14-*zorA*, T7p14-*zorB*, T7p14-*zorC*, 10 nM). The TXTL reactions were immediately pushed into the QCMD modules with SLBs on the sensor. **b** Adsorption kinetics of the blank (P70a-*T7rnap*, 0.15 nM) and ZorA (P70a-*T7rnap*, 0.15 nM and T7p14-*zorA*, 10 nM) conditions with either a pure DOPE or DOPE + 5.1% CL SLBs. **c** The adsorption kinetics of the blank (P70a-*T7rnap*, 0.15 nM), ZorA alone (P70a-*T7rnap*, 0.15 nM and T7p14-*zorA*, 10 nM), ZorB alone (P70a-*T7rnap*, 0.15 nM and T7p14-*zorB*, 10 nM), and ZorA and ZorB together (P70a-*T7rnap*, 0.15 nM, T7p14-*zorA*, 10 nM and T7p14-*zorB*, 10 nM) with a DOPE + 6.7% CL SLB. **d** The frequency changes after 8 hours of TXTL incubation, for the cases described in **c**. The bar plot level and error bars represent the mean and standard deviation of at least 3 replicates. **e** The adsorption kinetics of the blank (P70a-*T7rnap*, 0.15 nM) and ZorE (P70a-*T7rnap*, 0.15 nM and T7p14-*zorE*, 10 nM) conditions with either a pure DOPC or pure DOPE SLBs. **f** The adsorption kinetics of the blank (P70a-*T7rnap*, 0.15 nM) and ZorE (P70a-*T7rnap*, 0.15 nM and T7p14-*zorE*, 10 nM) conditions with either a pure DOPE or DOPE + 6.7% CL SLBs. **g** The adsorption kinetics of the blank (P70a-*T7rnap*, 0.15 nM), ZorAB (P70a-*T7rnap*, 0.15 nM, T7p14-*zorA*, 10 nM, and T7p14-*zorB*, 10 nM), and ZorABE (P70a-*T7rnap*, 0.15 nM, T7p14-*zorA*, 10 nM, T7p14-*zorB*, 10 nM, and T7p14-*zorE*, 5 nM) conditions with a DOPE + 6.7% CL SLB. **h** An illustration of the hypothetical localization of the Zorya proteins in the host *E. coli* inner membrane. All Zorya proteins preferentially interact with the CL-rich polar regions of the inner membrane. In addition, ZorA likely has a weak interaction with the equatorial region of the inner membrane and ZorE likely has a reversible interaction with the equatorial region of the inner membrane.

Recently, a plethora of novel microbial defense systems have been discovered in genomic defense islands^84,85^ and in phages^86^. Yet, their mechanisms of immunity largely remain unknown. One of such defense systems, type II Zorya, consists of ZorA, ZorB, and ZorE proteins that together protect their host *E. coli* from phage infection via an unknown mechanism^84^. ZorA and ZorB are likely IIMPs, as they contain predicted membrane-interacting domains (Fig. 6a) and are homologous to the flagellar motor proteins MotA and MotB^84^. In a recent cryo-EM study, the type I ZorA and ZorB proteins have been studied using cryo-EM and it has been determined that ZorA has three transmembrane domains, while ZorB has one^87^. Here, we demonstrated that the native Zorya system protects *E. coli* ATCC 8739 against phages T7 and PhiX174 (Fig. S31a). However, tagging the individual Zorya proteins for determining their localization and for subsequent purification disrupted their protective function (Fig. S31b–d), making this novel defense system particularly challenging to study. To overcome these limitations, we used the TXTL/QCMD approach to unravel the interactions of the Zorya proteins with lipid bilayers and to shed light on its mechanism of immunity.

ZorA, ZorB, and ZorE were synthesized through the T7 transcriptional activation cascade (Fig. 6a). The interaction of ZorA with SLBs was enhanced when CL was added to DOPE SLBs (Fig. 6b). However, adding DOPG to DOPE SLBs did not result in improved ZorA interaction (Fig. S32). Expectedly, ZorA did not interact with a DOPC SLB suggesting a preference for bacterial lipids (Fig. S33). When ZorA and ZorB were synthesized together, they produced a larger QCMD frequency shift than either of them alone, which indicates that they form a complex at the membrane (Fig. 6c, d). This is consistent with the cryo-EM result of type I ZorA and ZorB forming 5:2 ZorAB complexes similar to MotAB^87^, but additionally demonstrates that each protein can be incorporated in the bilayer on its own. On an ECL SLB, ZorA produces a small frequency shift with or without ZorB (Fig. S34). The kinetics of such shift is fast, indicating that the concentration of CL in the ECL SLB is small compared to the one tested in DOPE SLBs.

Although ZorE is not predicted to have membrane-interacting domains^84^, ZorE protein interacted with a pure DOPE SLB but not with a pure DOPC SLB (Fig. 6e). As with ZorAB, ZorE interacted more strongly with a DOPE/CL SLB than a pure DOPE SLB (Fig. 6f). To determine the strength of the interaction with the SLBs, we flushed Tris NaCl though the QCMD chamber above the SLBs at the end of the TXTL reaction. We observed that the ZorE frequency change was preserved only when CL was present in the SLB. In the condition with a pure DOPE SLB, it appears that all the synthesized ZorE proteins interacting with the SLB have been removed during flush (Fig. S35). This suggests that ZorE interacts weakly on the surface with a pure DOPE membrane but interacts irreversibly with membranes containing cardiolipin. Finally, the co-synthesis of ZorE with ZorAB did not result in a stronger interaction with a DOPE/CL SLB than ZorAB alone (Fig. 6g).

Overall, our results suggest that the ZorABE proteins likely localize at the poles of *E. coli* where cardiolipin is most dense (Fig. 6h). ZorE likely diffuses along the surface of the inner membrane and associates with the membrane upon finding cardiolipin-rich domains. Considering that Zorya protects *E. coli* from T7 and that T7 preferably binds *E. coli* cells at the poles^88^, it is possible that the ZorA and ZorB proteins disrupt the membrane potential that T7 uses to inject the first segment of its genome into the cell^89^. Alternatively, ZorAB may sense membrane depolarization caused by T7 infection and activate ZorE, which contains a predicted HNH domain (Fig. 6a), as a putative downstream effector to enact immunity.

## Discussion

The QCMD-TXTL approach for characterizing IIMPs’ interactions with lipid bilayers has several advantages compared to the current methods (Table 1). The approach is fast, non-disruptive, and reproducible. It enables assaying single or concurrently in situ synthesized IIMPs on membranes with arbitrarily complex lipid compositions on physical scales that tolerate the formation of large dynamical patterns. The QCMD-TXTL method proves to be useful for unraveling basic underpinning details about how IIMPs interact with different lipids and assemble at or into membranes. This method also provides information for engineering synthetic membranes capable of hosting IIMPs that naturally reside in different lipid environments. Taken altogether, these features make the QCMD-TXTL approach a highly favorable method for characterizing IIMPs membranes interactions. We anticipate that it could be useful for the bottom-up engineering of systems such as synthetic cells^43,90–93^

**Table 1:** Comparison of techniques for cell-free synthesized IIMP characterization

	Techniques for characterizing IIMPs with TXTL				
	SDS PAGE	Microscopy	AFM	SPR	QCM^a^
Label-free	✓	X	✓	✓	✓
Real-time TXTL	X	✓^106^	X	✓^107^	✓
Spatially resolved	X	✓^108^	✓^109^	X^b^ ^110^	X
IIMPs interactions	✓^111^	✓^112^	✓^113^	✓^114^	✓
SLB complexity^c^	✓^31^	✓^31^	✓^31^	✓^31,115^	✓
Sensitivity	100 ng^116^	SM^d^^117^	SM^d^^118^	1 ng^119^	1 ng
Penetration depth	N/A	N/A	10 nm^120^	200 nm^121^	250 nm^122^
Preparation time	2 d^123^	3 h^124^	1 d^23^	1 d^e^ ^125^	3 h

^a^The information for QCMD is from this study.

^b^SPR can have spatial resolution, but this modality is not used for IIMP characterization.

^c^The supported lipid bilayer (SLB) complexity refers to whether any type of lipid compositions can be achieved.

^d^Single molecule (SM).

^e^Potentially, the preparation time of SPR can be reduced to the same time as QCM experiments, but no studies utilizing the SALB-TXTL approach with SPR have been reported.

In the field of *E. coli* cell-free expression systems, it is established that *E. coli* inner membrane proteins integrate into lipid membranes without secretion mechanisms via a process coupled to translation^94,95^. This assumption is supported by the fact that IIMPs also integrate into lipid membranes when they are synthesized with the PURE system deprived of any secretion components^96^. In lysate-based TXTL systems, it is not clear whether soluble parts of the secretion mechanism present in the lysate (e.g., SecA is present in *E. coli* lysates^35^) help IIMPs integrate into membranes. In this work, we used an *E. coli* TXTL system, commercially available under the name of myTXTL (Arbor Biosciences). Other TXTL systems could be used if protein synthesis yields are large enough, which should be on the order of at least 100 nM based on our measurements and estimations (Fig. S11). We anticipate that most of the IIMPs from bacteria could be assayed using TXTL systems from *E. coli* or other bacteria, many of which have been reported in the literature^97^. Studying IIMPs from higher organisms with the QCMD-TXTL method remains to be investigated and is likely to be dependent on the complexity of the post-translation modifications of each IIMP. The scope of the IIMPs assayed in this work was chosen to demonstrate (i) that the QCMD-TXTL method corroborates many observations previously reported, (ii) and that novel insights and discoveries into the IIMPs-membranes’ interactions can be rapidly obtained. For instance, deciphering the Zorya system supports that many other novel defense systems could be deciphered using this approach.

The QCMD provides two signals, the change in frequency and the dissipation. In our work, we only used the change in frequency change ∆f as it is proportional to the mass added to the SLB. The dissipation term gives information about the viscoelastic changes of the lipid bilayer. As opposed to the frequency change, however, the dissipation term is much more difficult to understand and has not been subject to a comprehensive characterization in the literature. Extensive studies based on model systems would be necessary to make dissipation a truly interpretable term.

The preparation of the SLBs was performed using SALB in all of our experiments. Other works have used standard characterization techniques to demonstrate that SALB results in homogenous and fluid SLBs without any pervasive defects, such as the abundant micelles on the sensor surface instead of an SLB^29,31,98,99^. It is critical that the concentration of the lipids used during SALB is above the minimal concentration for complete coverage for TXTL. Using a SALB concentration two-fold larger than the minimal concentration does not appear to affect the kinetics of the TXTL experiments (Fig. 2c–e). However, previous works have shown that using lipid concentrations that are 16-fold larger than the minimum results in the formation of lower-quality SLBs likely due to the aggregation of micelles^98^, which accentuates the importance of determining the minimal concentration for each lipid type used in SALB. We demonstrated that blank TXTL reactions (no DNA added) resulted in replicable kinetics that could be routinely used as negative controls for lipid-protein interaction experiments. It would be of interest to also understand what exactly occurs between the biomolecules in the TXTL reaction and the lipid bilayer. We have demonstrated that the lipids are not being expelled from the SLB at a noticeable rate during TXTL incubation (Fig. S13), but additional measurements that could determine the packing and curvature of the SLB would assist in understanding how lipid bilayers respond to insertion or binding of TXTL biomolecules and TXTL-synthesized IIMPs. Also, the characterization of the electrostatic interactions between TXTL and SLBs is of significance, considering that the addition of charged lipids changed the kinetics of blank TXTL reactions in the QCMD.

We focused our work on using planar SLBs formed on the QCMD sensor. We showed that this configuration enables probing IIMPs’ interactions with membranes over a wide variety of lipids, including lipids localized in vivo, like cardiolipin. This configuration does not enable looking at specific variables found in living systems, such as membrane curvature for example. Instead of making flat SLBs, vesicles could be attached to the sensor, which has been done previously without TXTL^100^. We anticipate that dynamical patterns, such as the Min system, would be harder to achieve in this configuration, which would also require passivating the sensor to prevent nonspecific adsorption. Alternatively, SLBs can be fabricated on top of silica nanoparticles of controlled radii to mimic membrane curvature^101^.

## Methods

### Reagents

All the phospholipids were obtained from Avanti Polar Lipids in powder form. The phospholipids were dissolved in IPA (isopropyl alcohol, ThermoFisher Scientific, A416S-4) at the following stock concentrations: EggPC—100 mg/mL (840051 P), DOPC (1,2-dioleoyl-sn-glycero-3-phosphocholine)—100 mg/mL (850375 P), ECL (100500 P)—20 mg/mL, DOPE (1,2-dioleoyl-sn-glycero-3-phosphoethanolamine)—25 mg/mL (850725 P), DOPG (1,2-dioleoyl-sn-glycero-3-phospho-(1’-rac-glycerol))—10 mg/mL (840475 P), CL (1’,3’-bis[1,2-distearoyl-sn-glycero-3-phospho]-glycerol)—5 mg/mL (710334 P), DOTAP (1,2-dioleoyl-3-trimethylammonium-propane)—10 mg/mL (890890 P), DPPS (1,2-distearoyl-sn-glycero-3-phospho-L-serine) - 50 mg/mL (840029 P), PEG5000-PE (1,2-dipalmitoyl-sn-glycero-3-phosphoethanolamine-N-[methoxy(polyethylene glycol)-5000])—0.5 mg/mL (880200 P). To prepare the working IPA-lipid mixes, an appropriate volume of the stock phospholipids was diluted in IPA to the desired concentration, vortexed at medium-high speed for ten seconds, and immediately used for SALB.

### Oligos

All of the following oligos have been ordered from IDT.

MinC-s1: CTTTAAGAAGGAGAGGTACCAatgTCAAACACGCCAATCGAG

MinC-as1: CGGCGGGCTTTGCTCGAGTTTGACCGTTCAACCGTTAAATtga

MinD-s1: CTTTAAGAAGGAGAGGTACCAatgGCACGCATTATTGTTGTTACTTCG

MinD-as1: CGGCGGGCTTTGCTCGAGttaTCCTCCGAACAAGCGTTTGA

MinE-s1: CTTTAAGAAGGAGAGGTACCAatgGCATTACTCGATTTCTTTCTCTCG

MinE-as1: CGGCGGGCTTTGCTCGAGttaTTTCAGCTCTTCTGCTTCCGG

ZorA-s1: CTTTAAGAAGGAGAGGTACCAatgttagcgcagctttttgagc

ZorA-as1: CGGCGGGCTTTGCTCGAGgatcgattaccctcgatgtttattagcag

ZorB-s1: CTTTAAGAAGGAGAGGTACCAatggataagattatagggaaacaattaccta

ZorB-as1: CGGCGGGCTTTGCTCGAGttactcctgaataatctttctaatctgcaactcg

ZorE-s1: CTTTAAGAAGGAGAGGTACCAatgaaattatctatcgacatttcagaacttattcaa

ZorE-as1: CGGCGGGCTTTGCTCGAGttacaattttgctggcgtaaaggct

T7p14-UTR1-s1: GATCGAGATCTCGATCCCGCGAAATTAATACGACTCACTATAGGGAGACCACAACG

GTTTCCCTCTAGAAATAATTTTGTTTAACTTTAAGAAGGAGAGGTACCAATG

P70a-UTR1-s1: GTTCCGCTGGGCATGCTGAGCTAACACCGTGCGTGTTGACAATTTTACCTCTGGCGG

TGATAATGGTTGCAGCTAGCAATAATTTTGTTTAACTTTAAGAAGGAGAGGTACCA

ATG

T500-as1: GTCGACACAGAAAAGCCCGCCTTTCGGCGGGCTTTGCTCGAG

### Cell-free transcription-translation

Cell-free gene expression was carried out using an *E. coli* TXTL system described previously^27^, with one modification. We used the strain BL21-∆*recBCD* Rosetta2 in which the *recBCD* gene set is knocked out to prevent the degradation of linear DNA^102^. The preparation and usage of the TXTL system were the same as reported before^27,103^. Briefly, *E. coli* cells were grown in a 2xYT medium supplemented with phosphates. Cells were pelleted, washed, and lysed with a cell press. After centrifugation, the supernatant was recovered and preincubated at 37 °C for 80 min. After a second centrifugation step, the supernatant was dialyzed for 3 h at 4 °C. After final centrifugation; the supernatant was aliquoted and stored at −80 °C. The TXTL reactions comprised the cell lysate, the energy and amino acid mixtures, maltodextrin (30 mM) and ribose (30 mM), magnesium (2–5 mM) and potassium (50–100 mM), PEG8000 (1–2 wt%), water and the DNA to be expressed. The TXTL reactions were incubated at 30 °C in QCMD chambers (40 µL reactions). The TXTL reactions were incubated at 30 °C on 96 well plates (2 µL reactions) for the measurement of the kinetics of deGFP synthesis.

### Reporter protein quantification

deGFP, AH-eGFP, and LactC2-eGFP were quantified on an Agilent Biotek H1M plate reader using a calibration curve. The calibration curve was determined using pure eGFP (Cell Biolabs, STA-201) diluted with an empty TXTL reaction at various concentrations.

### DNA preparation

All the DNA sequences are reported in the Supplementary Data 2. We either used plasmids or linear DNAs. DNAs were obtained either by PCR or purchased (Twist Biosciences, IDT). DNA stock solutions were quantified with a spectrophotometer (ThermoFisher Scientific, NanoDrop 2000). Genes were expressed either from the strong *E. coli* promoter P70a or the T7 transcriptional activation cascade^25–27^.

### QCMD sensors and modules preparation

A QSense Analyzer (Biolin Scientific, Gothenburg, Sweden) was used for the experiments. The QSense Analyzer has four channels that can be used independently and concurrently. The preparation of the instrument for an experiment consisted of a 400 µL/min flush of at least 20 mL of a 1% wt SDS solution, then 20 mL of autoclaved deionized water, and then air until the tubing was empty. After the four QCMD modules were dried, they were disconnected from the QSense Analyzer mount and opened. After removing the sensors (Biolin Scientific, QSX303), the QCMD modules were dried with nitrogen. The sensors were dried with nitrogen as well and plasma-cleaned (Harrick Plasma, PDG-32) at low RF power for 10 minutes. The plasma-cleaned sensors were then reinserted into the dry QCMD modules. The QCMD modules were closed and connected to the QSense Analyzer mount. Using the QSoft software, the bare sensors were calibrated and the QCMD modules were ready.

### QCMD SLB preparation and TXTL reaction

The QCMD modules were maintained at 29 °C throughout the whole experiment. First, a Tris NaCl Buffer (10 mM Tris, 150 mM NaCl, 7.5 pH) was flushed at 100 µL/min through all the modules until the resonance frequency of the sensors stabilized, which took 40–60 minutes. Next, IPA was flushed until signal stabilization, which takes around 20 minutes and produces a change in frequency of ~ −75 to −80 Hz. We then flushed the IPA-lipid mixes at 100 µL/min for 20 minutes. After stabilization, the signal varies by −5 to −10 Hz, depending on the lipids used, usually within 5–10 minutes, except for ECL which requires the full duration of the flush. Next, we flushed the Tris NaCl buffer at 100 µL/min until signal stabilization to complete the formation of the SLB. This step takes up to 40 minutes and produces a change of frequency of the order of 30–50 Hz, depending on the lipids used. Finally, we pushed the TXTL reactions (up to four different conditions, one per module) at 25 µL/min. The pumps were stopped and the TXTL reactions were incubated in contact with the SLB-sensor system for 3–20 h.

### QCMD data analysis

The analysis of QSense Analyzer data was performed on the 7th overtone of the resonance of the sensor due to its lowest sensitivity to variations in the mounting of the sensor, following the manufacturer’s recommendation. The frequency at the end of the second Tris NaCl flush during the SLB preparation was reset as Δ*f* = 0. The frequency output of the seventh harmonic was divided by 7 to reflect the effect the TXTL reactions had on the fundamental frequency changes of the sensor. Example python code: https://zenodo.org/records/12752763.

### Zorya knockout mutants

Zorya genes *zorABE* were deleted from the genome of *Escherichia coli* DSM 1576 (ATCC 8739) using lambda red recombination as described^104^. The genes were deleted in the region of the chromosome between 4,265,744 and 4,269,528 bp (NCBI Ref. CP000946).

### Plaque assay

Double layer plaque assay was used to determine phage titers. *E. coli* were cultivated in LB medium until the OD_600_ of 0.55 before centrifugation. The pellet was resuspended in cold LB, mixed with soft agar, and the appropriate phage dilution. This mixture was added to an agar plate and solidified. Subsequently, phage dilutions were directly spotted on the plate. The plates were incubated at 37 °C until visible plaques developed within 4 to 18 hours.

### Statistics and reproducibility

The QCMD collects four measurements at a time. Because of this speed of new data collection, we prioritized the replication of the most critical experiments. For these experiments, we collected at least three independent replicates and either showed each data point and, in some cases, also the mean and standard deviation of the replicates. For the experiments that served to assist the main message we sometimes did not collect replicates due to the throughput of the QCMD. However, those experiments are meant to identify trends and thus are always presented as comparisons to data for which replicates have been collected (example: Fig. 4b).

### Reporting summary

Further information on research design is available in the Nature Portfolio Reporting Summary linked to this article.

### Supplementary information

Supplementary Information

Description of Additional Supplementary File

Supplementary Data 1

Supplementary Data 2

Reporting Summary

## Data Availability

The data used in Figs. 2–6 and Supplementary Figs. S1–S35 are provided as source data files in Supplementary Data 1. Plasmids and strains used in this study are listed in Supplementary Data 2. All experimental data are stored on UMN DRUM and can be accessed here: https://hdl.handle.net/11299/264081.

## Code availability

The data analysis of the QCMD data in this study were performed with Python. The analysis is the same for all QCMD files and an example of code can be accessed on Zenodo (https://zenodo.org/records/12752763)^105^.
