# Supplementary material for: Cell-free expression with a quartz crystal microbalance enables rapid, dynamic, and label-free characterization of membrane-interacting proteins
Source: Commun Biol. 2024 Aug 17;7:1005. doi: 10.1038/s42003-024-06690-9 (PMC11329788; doi:10.1038/s42003-024-06690-9)
Supplement: Supplementary file 1 — Supplementary Information [file 42003_2024_6690_MOESM1_ESM.pdf]

Cell-free expression with a quartz crystal microbalance enables rapid, dynamic, and label-free characterization of membrane-interacting proteins

Aset Khakimzhan[1], Ziane Izri[1], Seth Thompson[1], Oleg Dmytrenko[2], Patrick Fischer[2], Chase Beisel[2,3], Vincent Noireaux[1,*]

[1]School of Physics and Astronomy, University of Minnesota, Minneapolis, MN 55455, USA
[2]Helmholtz Institute for RNA-based Infection Research (HIRI), Helmholtz-Centre for Infection Research (HZI), 97080 Würzburg, Germany
[3]Medical Faculty, University of Würzburg, 97080 Würzburg, Germany

[*]**Corresponding author:** Email: noireaux@umn.edu

Item	Title	Pages
Figure S1	TXTL lysate does not contain living E. coli cells	2
Figure S2	Synthesis of deGFP from linear templates with a P70a promoter or a T7 cascade	3
Figure S3	SALB procedure with and without lipids during step III	4
Figure S4	Nonspecific TXTL adsorption for model SLBs	5
Figure S5	Measuring the interaction of blank TXTL reactions with SLBs	6
Figure S6	MscL TXTL adsorption replicates with ECL SLB	7
Figure S7	AH-eGFP TXTL adsorption replicates with EggPC SLB	8
Figure S8	Specific adsorption of MscL and AH-eGFP into DOPC and DOPE SLBs	9
Figure S9	Pre-synthesized MscL does not integrate into SLBs	10
Figure S10	Pre-synthesized AH-eGFP does integrate into SLBs	11
Figure S11	Post-QCMD fluorescence measurements	12
Figure S12	Mass sensitivity of the QCMD	13
Figure S13	Adsorption of blank, AH-eGFP, and MscL TXTL reactions onto ECL-EggPC SLBs	14
Figure S14	Adsorption of blank and MscL TXTL reactions into DOPE-DOPC SLBs	15
Figure S15	Quantification of lipid expulsion caused by TXTL reactions.	16
Figure S16	Nonspecific TXTL adsorption into DOPG-DOPE SLBs	17
Figure S17	Nonspecific TXTL adsorption into CL-DOPG-DOPC SLBs	18
Figure S18	Nonspecific TXTL adsorption into CL-DOPE SLBs	19
Figure S19	eGFP-LactC2 adsorption kinetics and approximate endpoint concentrations	20
Figure S20	Modes and wavelengths of oscillations produced by MinD	21
Figure S21	MinD adsorption kinetics in DOPG-DOPC SLBs	22
Figure S22	MinD adsorption kinetics in DOPG-DOPE SLBs	23
Figure S23	MinD adsorption kinetics in CL-DOPC SLBs	24
Figure S24	MinD adsorption kinetics in CL-DOPE SLBs	25
Figure S25	Effect of DOTAP on MinD adsorption kinetics on DOPC and DOPG-DOPC SLBs	26
Figure S26	Effect of DOTAP on MinD adsorption kinetics in DOPE and DOPG-DOPE SLBs	27
Figure S27	Number of oscillations in the first 2h in SLBs with added DOTAP	28
Figure S28	Effect of P70a-*minE* concentration on MinDE adsorption kinetics on a DOPG-DOPE SLB	29
Figure S29	Effect of P70a-*minE* concentration on MinDE adsorption kinetics on a DOPG-DOPC SLB	30
Figure S30	Effect of P70a-*minE* concentration on MinDE adsorption kinetics on an ECL SLB	31
Figure S31	Tagging of Zorya proteins disrupts *E. coli* defense against phages	32
Figure S32	Effect of DOPG on ZorA TXTL adsorption kinetics on a DOPE SLB	33
Figure S33	Comparison of ZorA adsorption kinetics with a DOPC and a DOPE SLB	34
Figure S34	ZorAB TXTL adsorption kinetics with an ECL SLB	35
Figure S35	ZorE adsorption kinetics with post-incubation Tris NaCl flush included in the graph	36
Table S1	Working lipid concentrations during step III of SALB	37

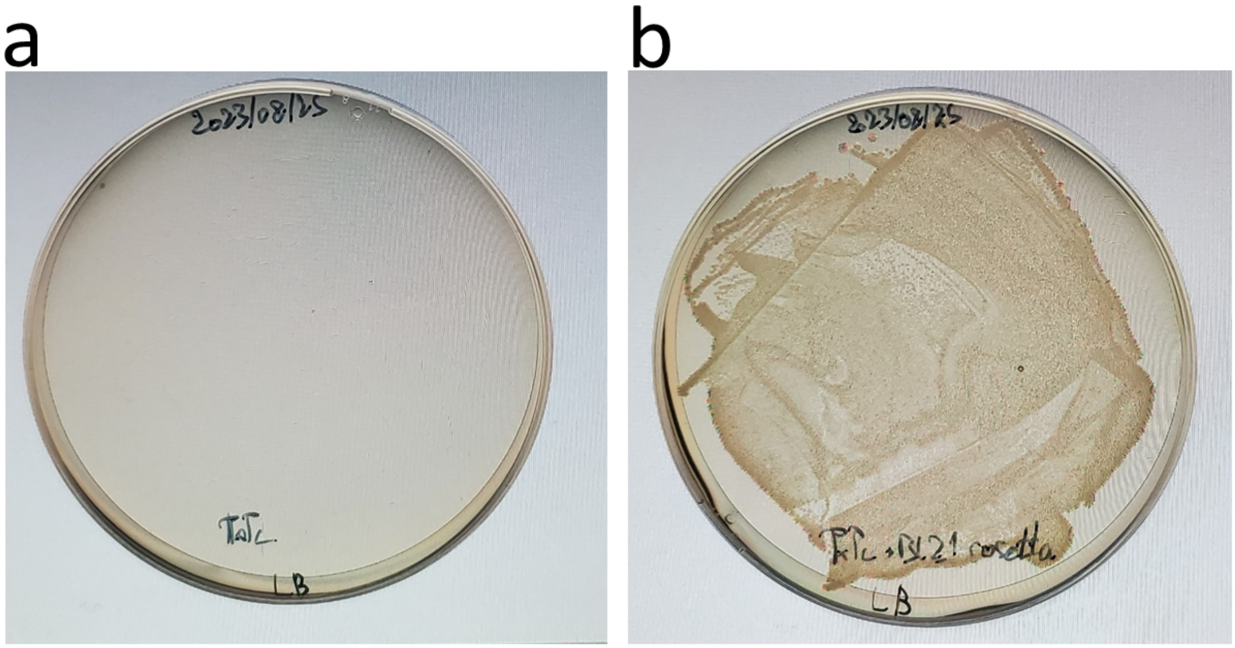

Figure S1. (a) Plating 45 µl of a TXTL reaction onto an agar plate with no antibiotic shows no *E. coli* colonies. **(b)** Adding 5 µl of BL21 Rosetta *E. coli* to 40 µl of a TXTL reaction and then plating the mix onto an agar plate with no antibiotic shows many *E. coli* colonies.

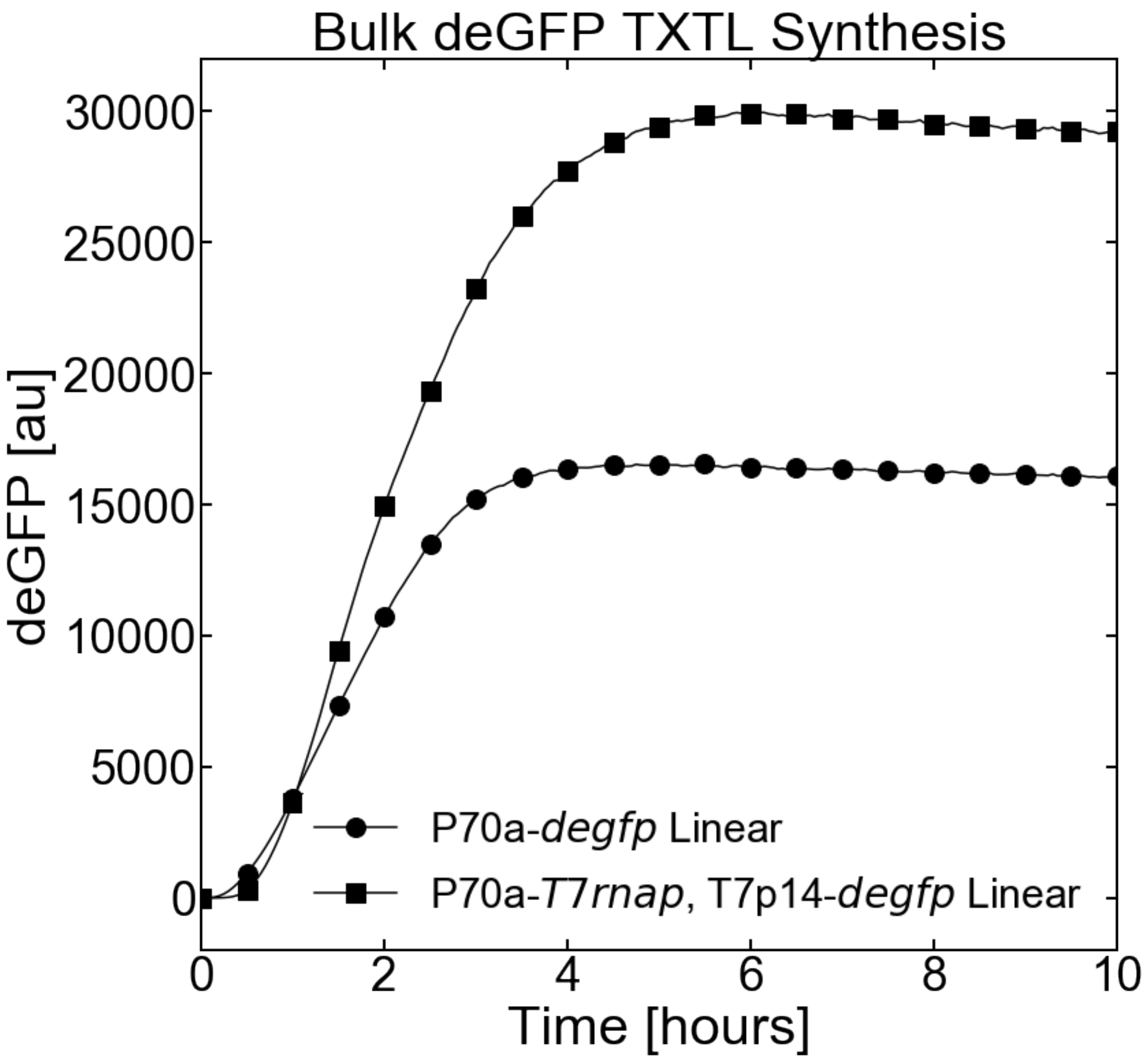

Figure S2. Batch mode TXTL of deGFP with the P70a promoter (P70a-*degfp*, 5nM) and with the T7 cascade (P70a-*T7rnap*, 0.15nM, T7p14-*degfp*, 5nM).

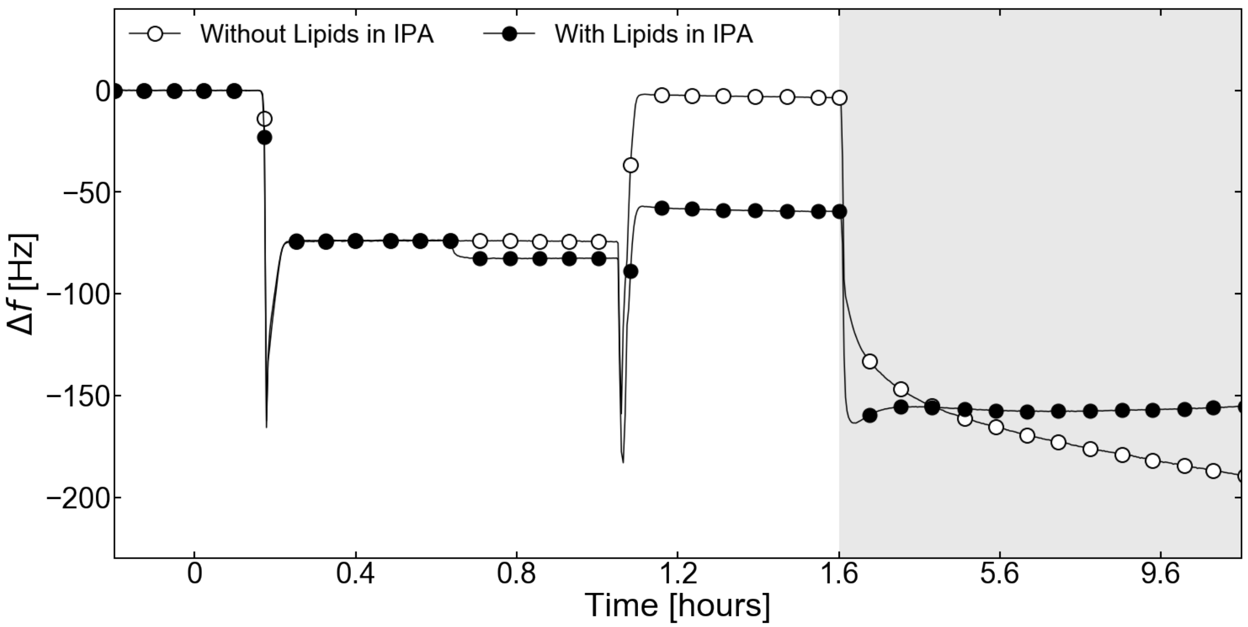

Figure S3. SALB protocol completed with ECL or without lipids added to the IPA-Lipid mix. Step III shows a return to Δf = 0 Hz, which indicates no SLB has been formed.

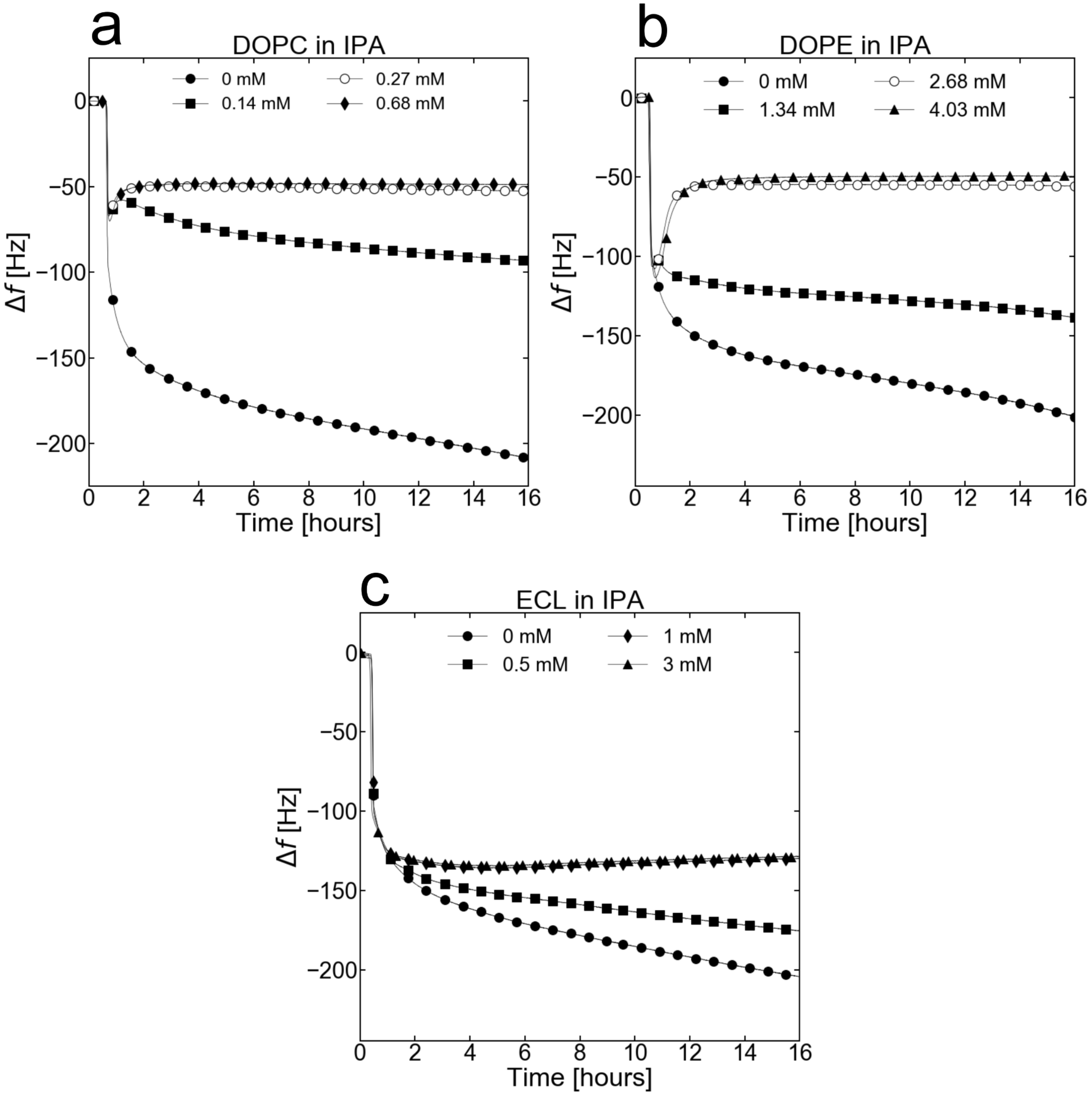

Figure S4. Adsorption kinetics of a blank TXTL reaction (P70a-*T7rnap*, 0.15 nM) on SLBs depending on the DOPC **(a)**, DOPE **(b)**, and ECL **(c)** phospholipids concentration in IPA during Step II of SALB.

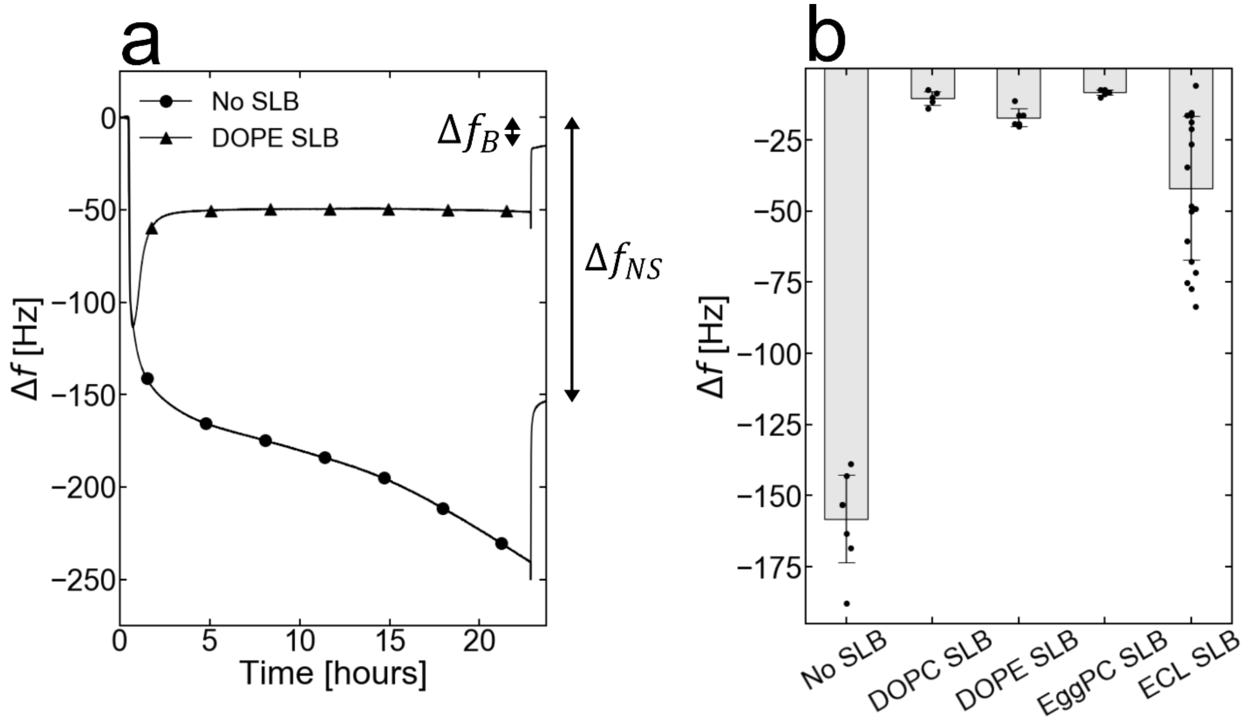

Figure S5. Measuring the interactions of blank TXTL reactions with SLBs. **(a)** Adsorption kinetics of blank TXTL reactions (P70a-*T7rnap*, 0.15 nM) in contact with either a bare sensor or with a DOPE SLB. The Tris NaCl buffer is flushed into the modules after the TXTL incubated for 22 hours. The difference in the frequency shift (Δf_B) between the Tris NaCl flush before TXTL and after the TXTL reaction quantifies the interaction strength of a blank TXTL reaction with the SLB. This value is compared to the difference obtained from nonspecific adsorption of TXTL to the bare sensor (Δf_{NS}). **(b)** Comparison of the mean Δf_B levels for the DOPC, DOPE, EggPC, and ECL SLBs to the Δf_{NS} levels obtained from bare sensors. The mean and the standard deviation are calculated from at least 5 replicates.

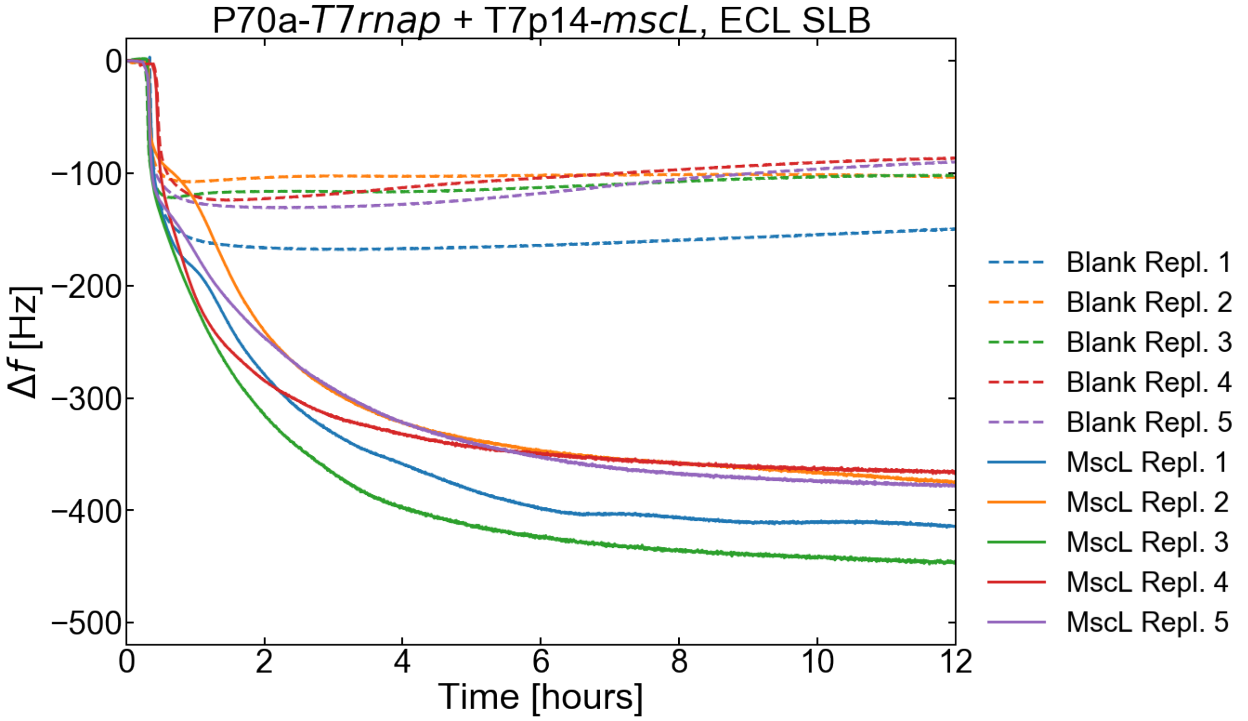

Figure S6. Replicates of the T7p14-*mscL* TXTL reaction (P70a-*T7rnap*, 0.15 nM, T7p14-*mscL,* 5 nM) incubated on an ECL SLB.

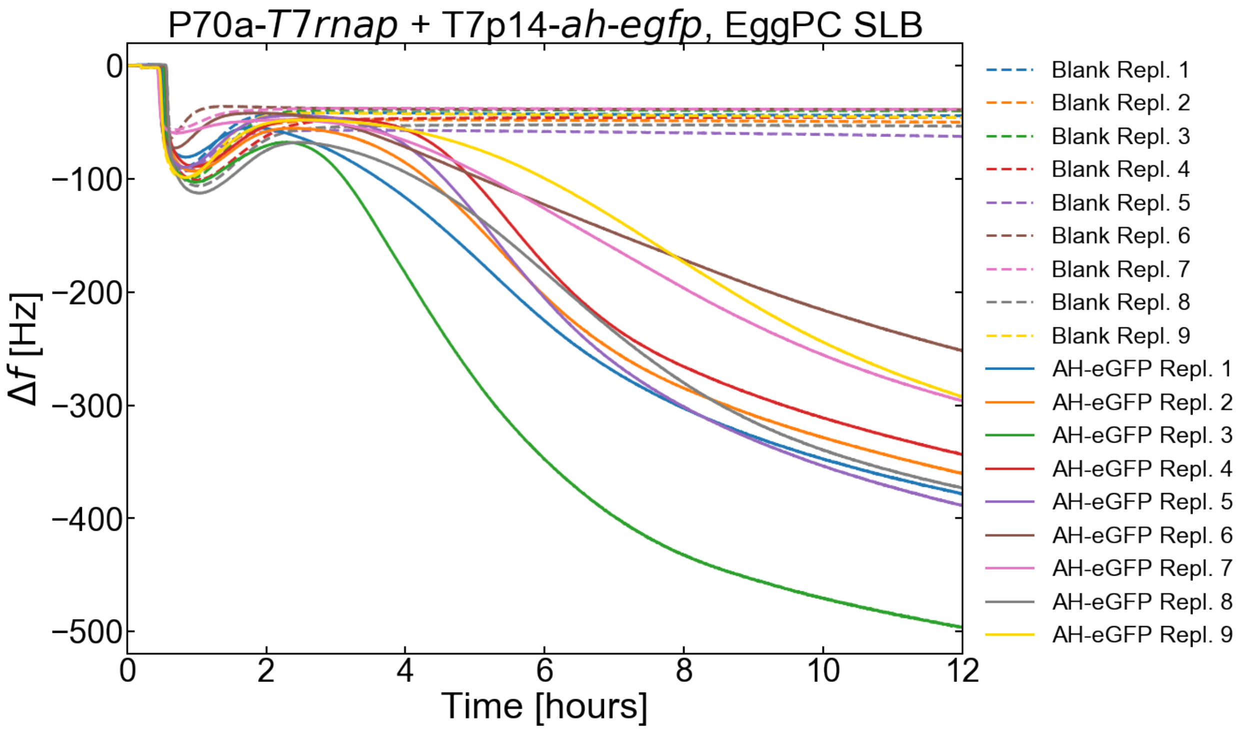

Figure S7. Replicates of the T7p14-*ah-eGFP* TXTL reaction (P70a-*T7rnap*, 0.15 nM, T7p14-*ah-egfp,* 5 nM) incubated on an EggPC SLB.

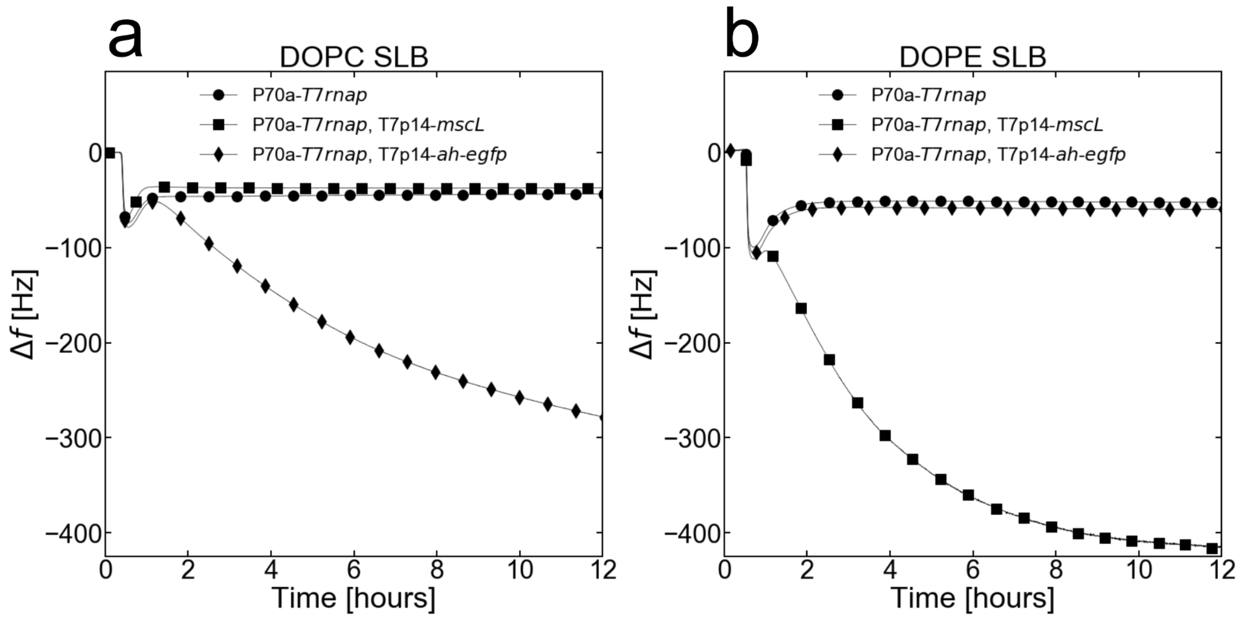

Figure S8. (a) and (b) Adsorption kinetics of either blank (P70a-*T7rnap*, 0.15 nM), AH-eGFP (P70a-*T7rnap*, 0.15 nM, T7p14-*ah-egfp,* 5 nM), or MscL (P70a-*T7rnap*, 0.15 nM, T7p14-*mscL,* 5 nM) TXTL reactions into a DOPC SLB and a DOPE SLB respectively.

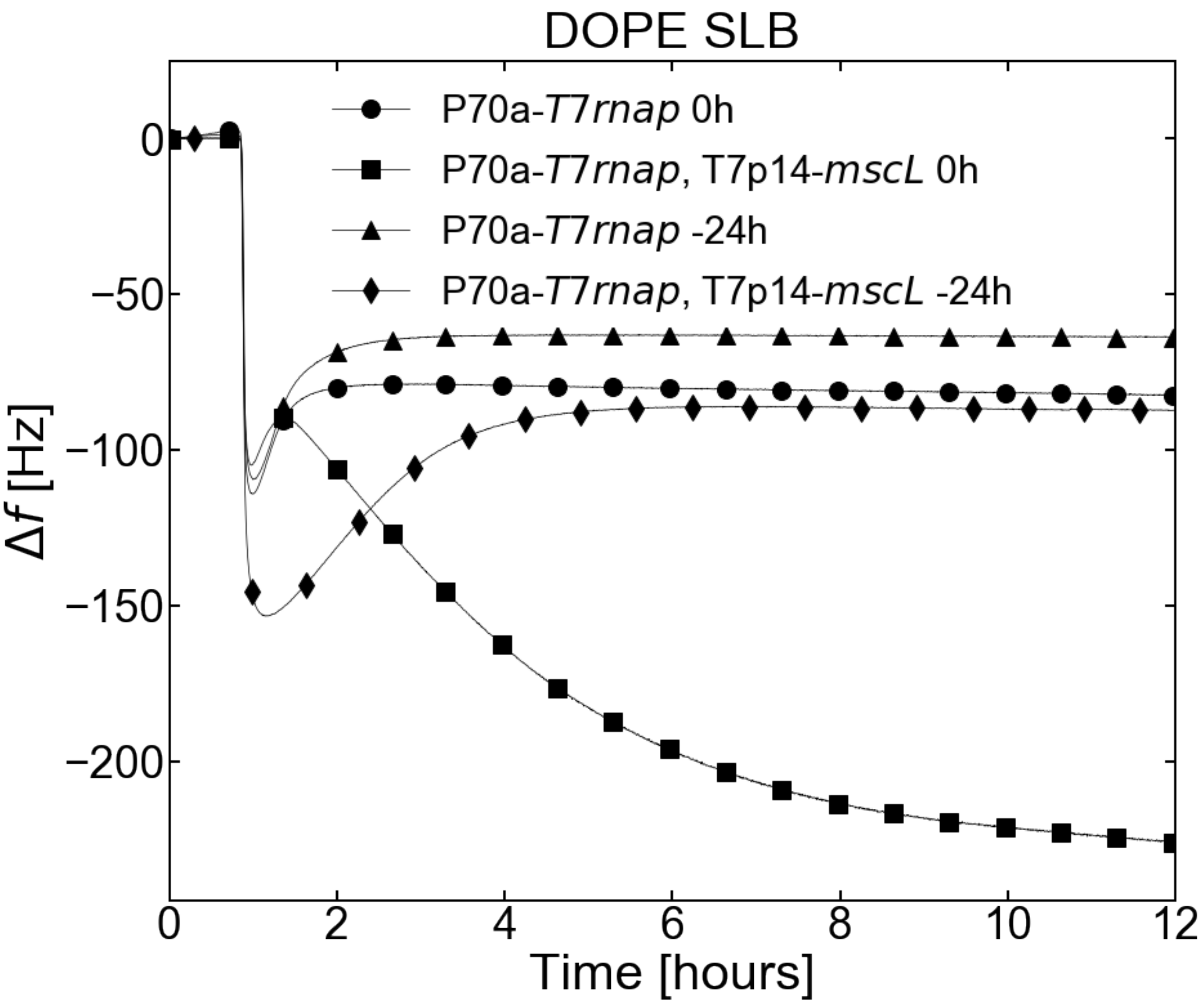

Figure S9. Adsorption kinetics into a pure DOPE SLB of a blank TXTL (P70a-*T7rnap*, 0.15 nM) and an MscL TXTL (P70a-*T7rnap*, 0.15 nM, T7p14-*mscL,* 5 nM) reactions that have either been freshly mixed (0 h) or that have been pre-incubated for 24 h (-24 h) before being flushed into the QCMD module.

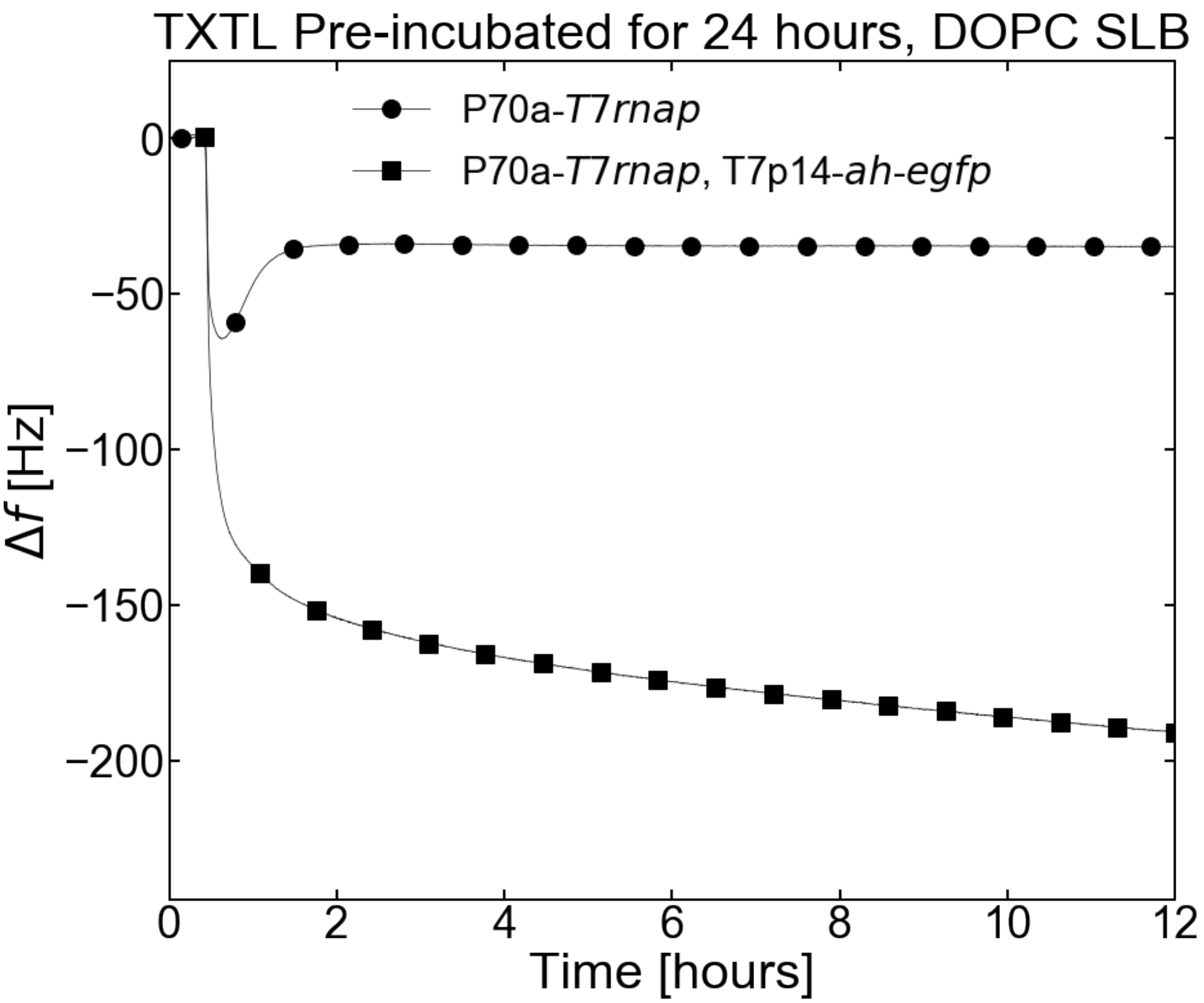

Figure S10. Adsorption kinetics onto a pure DOPC SLB of a blank TXTL (P70a-*T7rnap*, 0.15 nM) and an AH-eGFP TXTL (P70a-*T7rnap*, 0.15 nM, T7p14-*ah-egfp,* 5 nM), reactions that have been pre-incubated for 24 h before being flushed into the QCMD module.

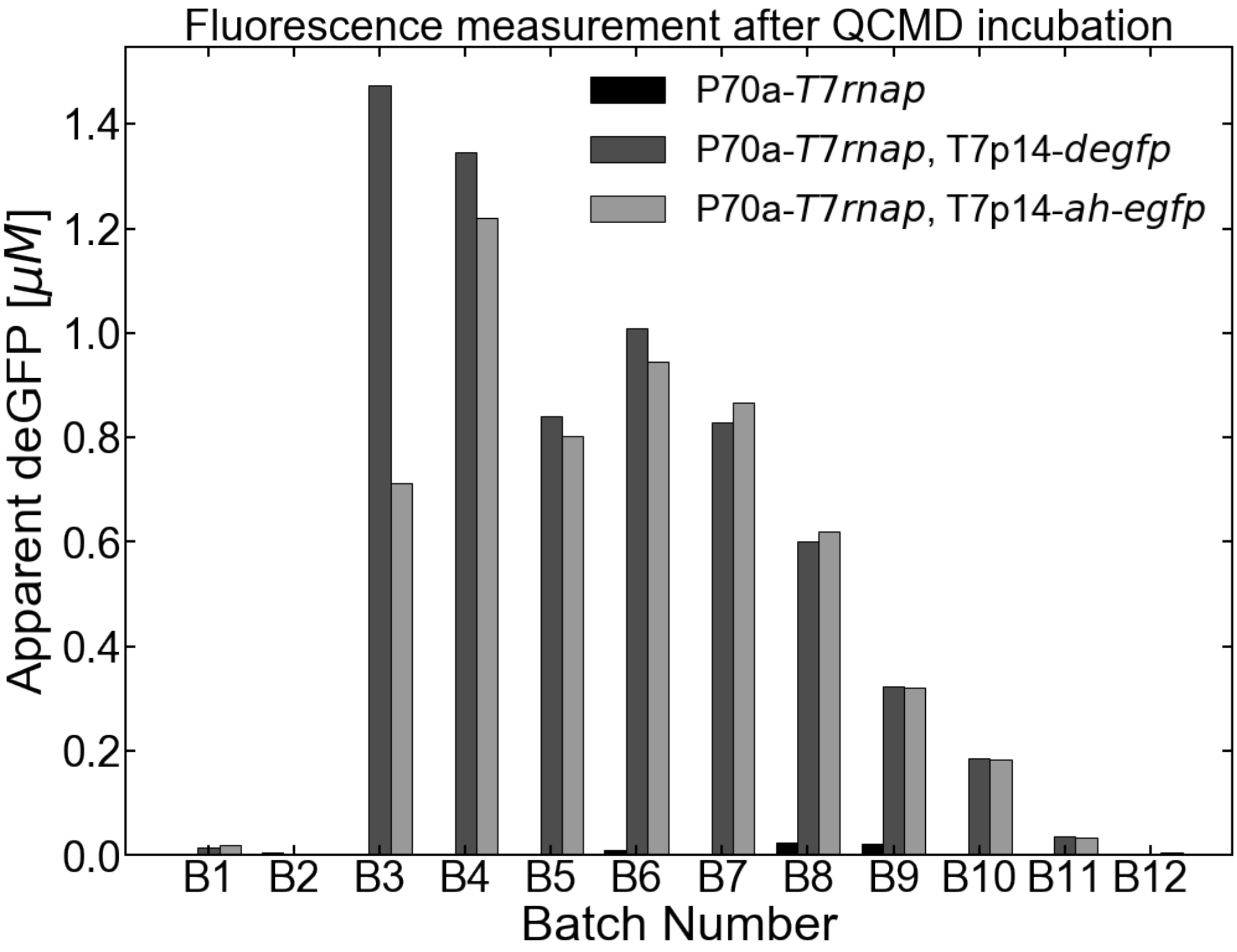

.

Figure S11. End-point measurement of either blank (P70a-*T7rnap*, 0.15 nM), AH-eGFP (P70a-*T7rnap*, 0.15 nM, T7p14-*ah-egfp,* 5 nM), or MscL (P70a-*T7rnap*, 0.15 nM, T7p14-*mscL,* 5 nM) TXTL reactions in the QCMD modules overnight. The TXTL is flushed out of the module in batches of 100 µl and the x-axis corresponds to the number of the batch.

Sensor dimensions
- Diameter 1.2 cm.<br>- Surface area = 1.131 10^{-4} m^2.
**MscL dimensions**
- MscL assembles into a pentamer.<br>- Molar mass of MscL monomer = 14957.2 g/mol.<br>- Mass of one monomer = 2.48 10^{-20} g.<br>- Mass of one pentamer = 1.24 10^{-19} g.<br>- Surface area of one pentamer: 140 nm^2 [1].
**Mass of closely packed MscL pentamers on the SLB**
Hypothesis: close packing of circular MscL pentamers on SLB:<br>- The size ratio of closely packed circles is about 0.6.<br>- The number of MscL pentamers on the SLB is 0.6 * 1.131 10^{-4} / (1.4 10^{-16}) = 4.85 10^{11}.<br>- The maximum total mass of MscL on the SLB is 60.1 ng.<br>- For a 40-µl reaction (volume of each QCMD chamber), this corresponds to a concentration of 1.5 µg/ml or 0.1 µM of MscL proteins. Therefore, based on our deGFP and AH-eGFP quantifications (1-2 µM produced in QCMD chambers), about 20 times more proteins are produced in the QCMD chamber with respect to the membrane capacity.<br>- We assume a resolution of 5 Hz from the QCMD frequency signal.<br>- For MscL the drop is of about 300 Hz.<br>- We can detect 60 times less MscL, which corresponds to about 1 ng, based on maximum membrane coverage with MscL.<br>- It is unlikely that MscL reaches this level of packing on our SLBs, so this mass sensitivity is likely to be underestimated.

Figure S12. Estimation of the mass sensitivity of the QCMD.

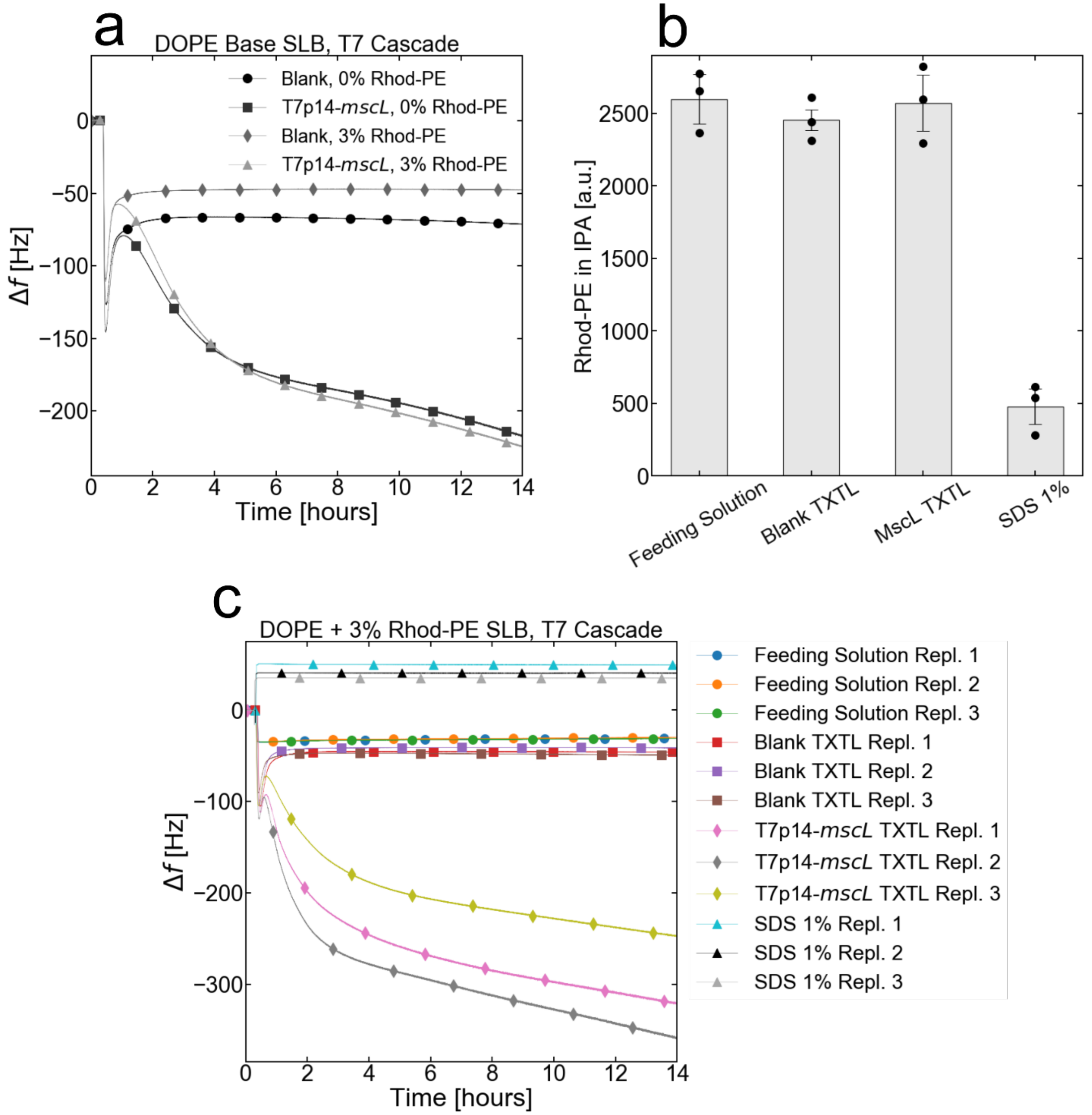

Figure S13. Quantification of lipid expulsion caused by TXTL reactions. **(a)** Adsorption kinetics of blank (P70a-*T7rnap*, 0.15 nM) and MscL (P70a-*T7rnap*, 0.15 nM, T7p14-*mscL*, 5 nM) TXTL reactions into DOPE and DOPE + Rhod-PE SLBs. **(b)** Quantification of IPA recovered Rhod-PE after overnight incubation of either a feeding solution (TXTL reaction without lysate), a blank TXTL reaction (P70a-*T7rnap*, 0.15 nM), an MscL TXTL reaction (P70a-*T7rnap*, 0.15 nM, T7p14-*mscL*, 5 nM), or 1% SDS. The mean and standard deviation are calculated from 3 replicates. **(c)** QCMD adsorption kinetics of the conditions from **(b)**.

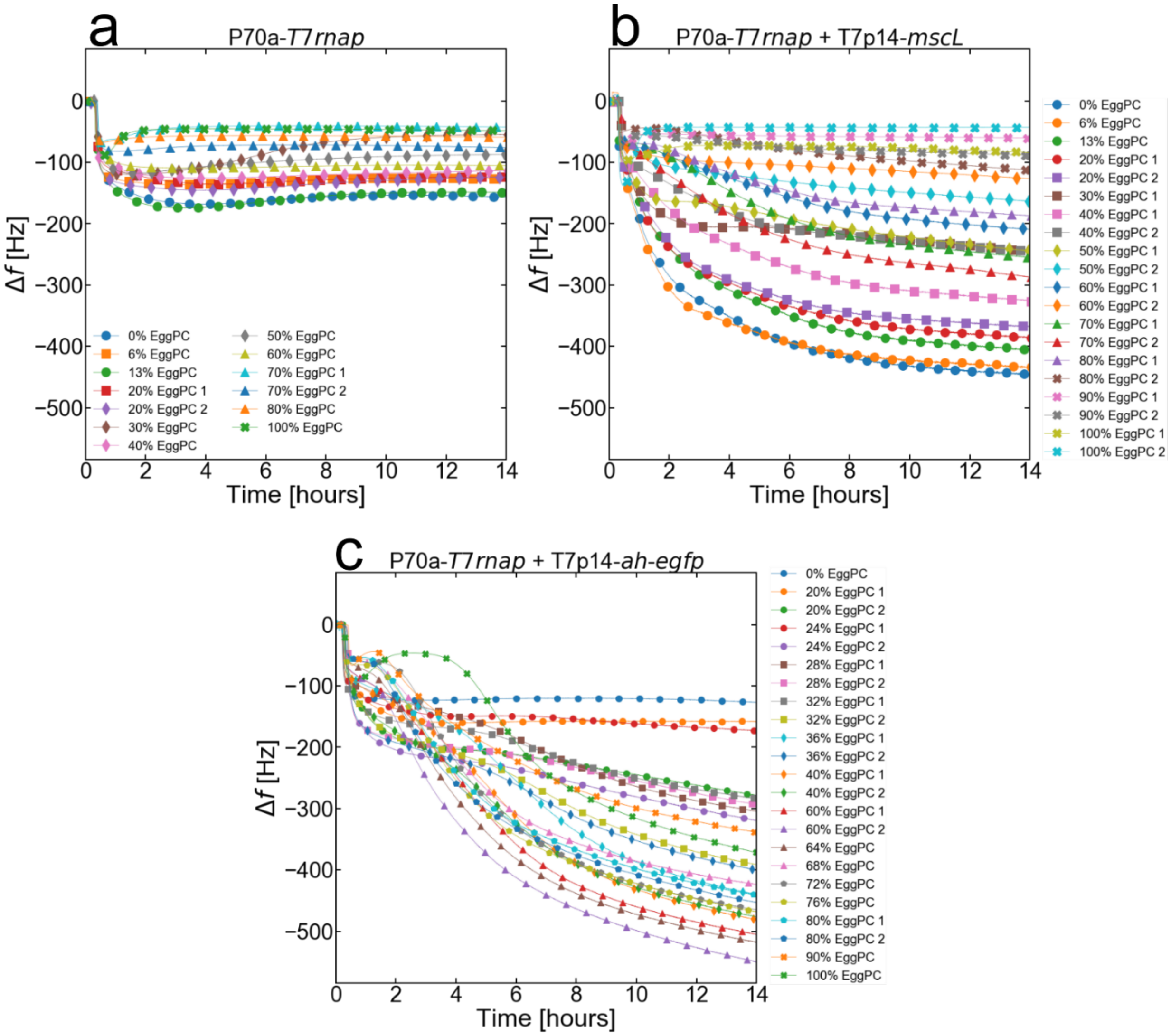

Figure S14. Adsorption kinetics of **(a)** a blank (P70a-*T7rnap*, 0.15 nM), **(b)** an MscL (P70a-*T7rnap*, 0.15 nM, T7p14-*mscL*, 5 nM), and **(c)** an AH-eGFP (P70a-*T7rnap*, 0.15 nM, T7p14-*ah-egfp*, 5 nM) TXTL reactions respectively into ECL – EggPC hybrid SLBs.

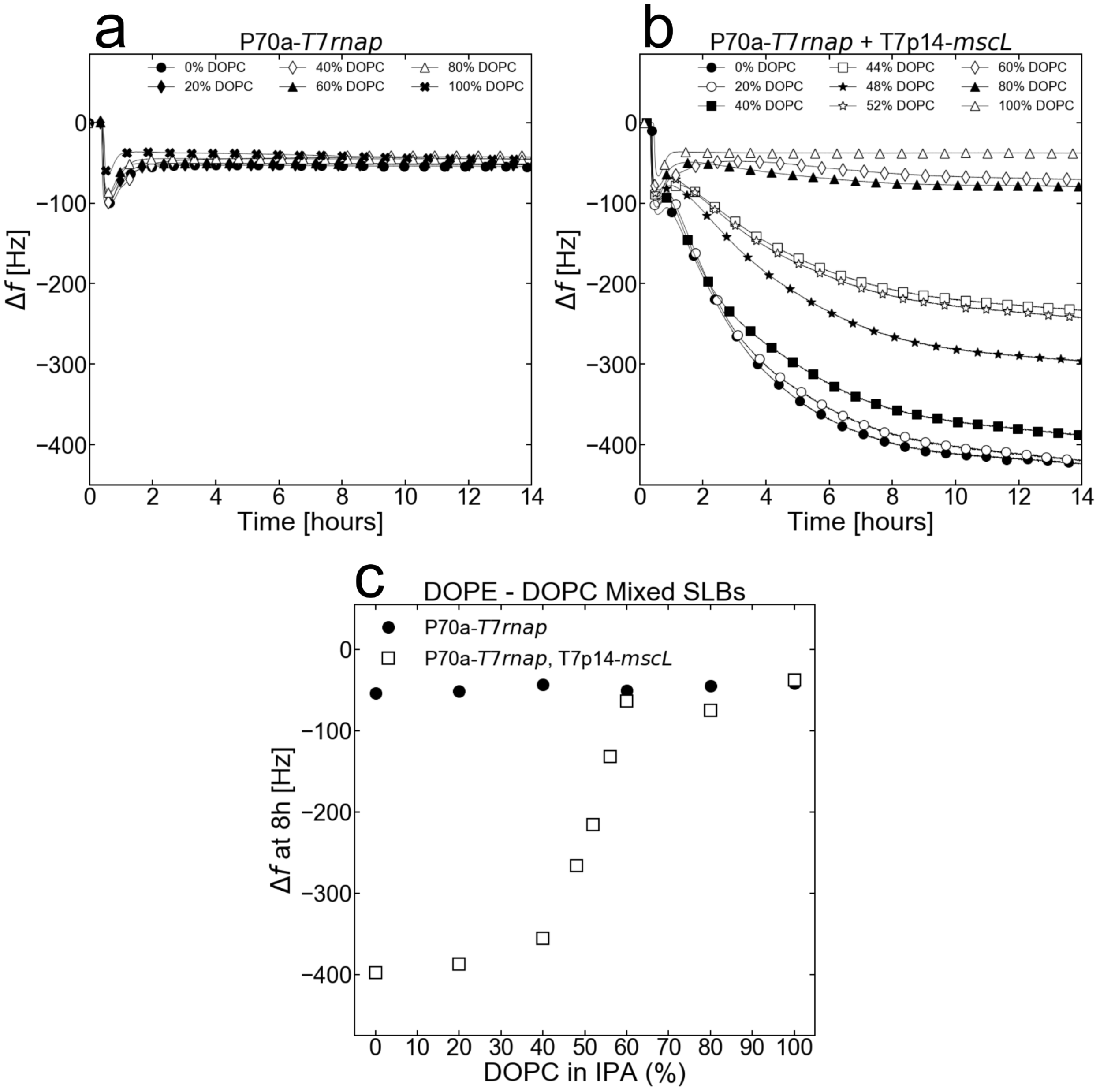

Figure S15. (a) and (b) Adsorption kinetics of blank (P70a-*T7rnap*, 0.15 nM) and MscL (P70a-*T7rnap*, 0.15 nM, T7p14-*mscL*, 5 nM) TXTL reactions respectively into DOPE – DOPC Mixed SLBs. **(c)** The frequency changes after 8 h of incubating either blank (P70a-*T7rnap*, 0.15 nM) or MscL (P70a-*T7rnap*, 0.15 nM, T7p14-*mscL*, 5 nM) TXTL reactions into DOPE – DOPC Mixed SLBs.

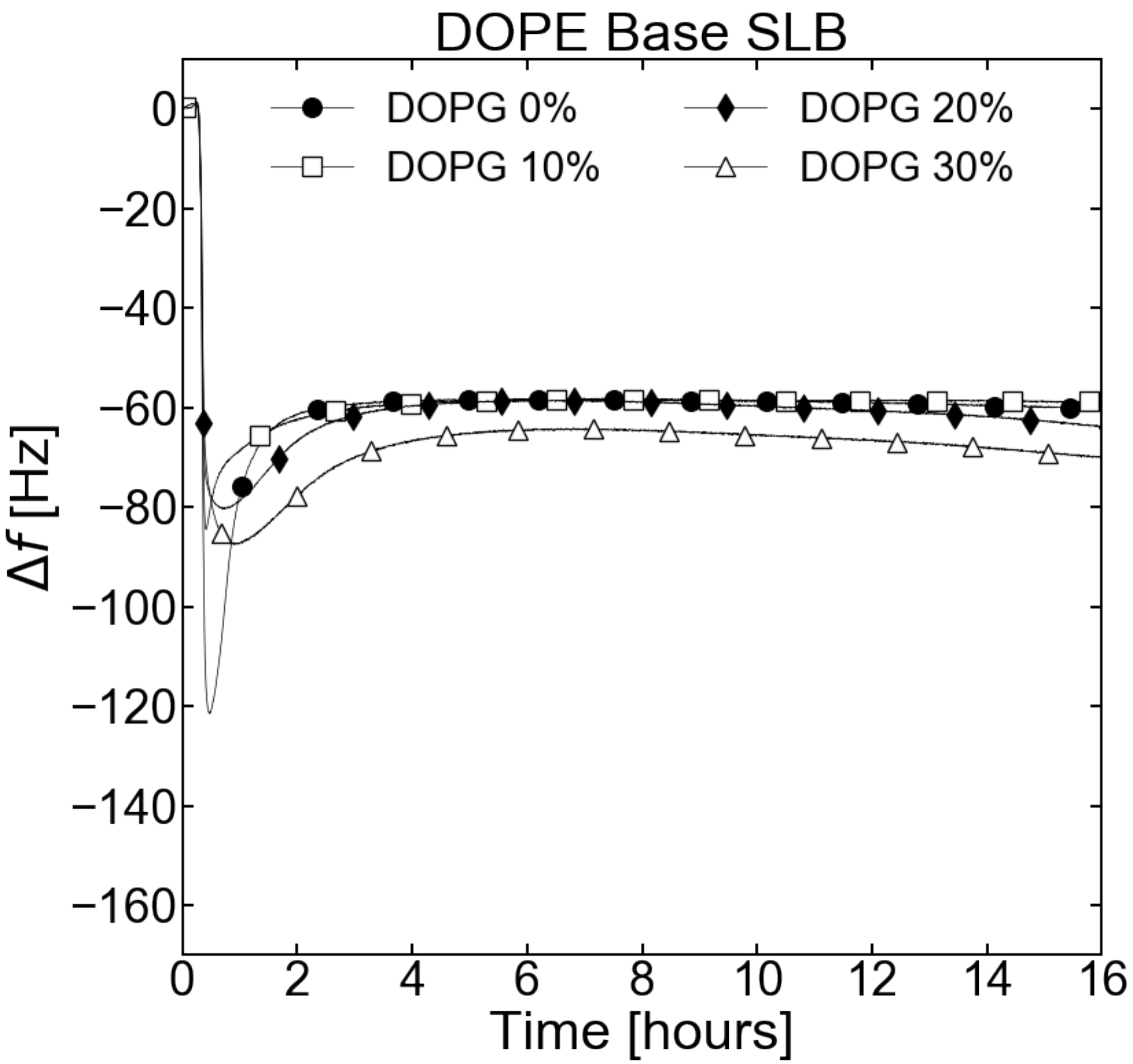

Figure S16. Adsorption kinetics of a blank TXTL reaction (P70a-*T7rnap*, 0.15 nM) onto 0, 10, 20, and 30% DOPG/DOPE SLBs

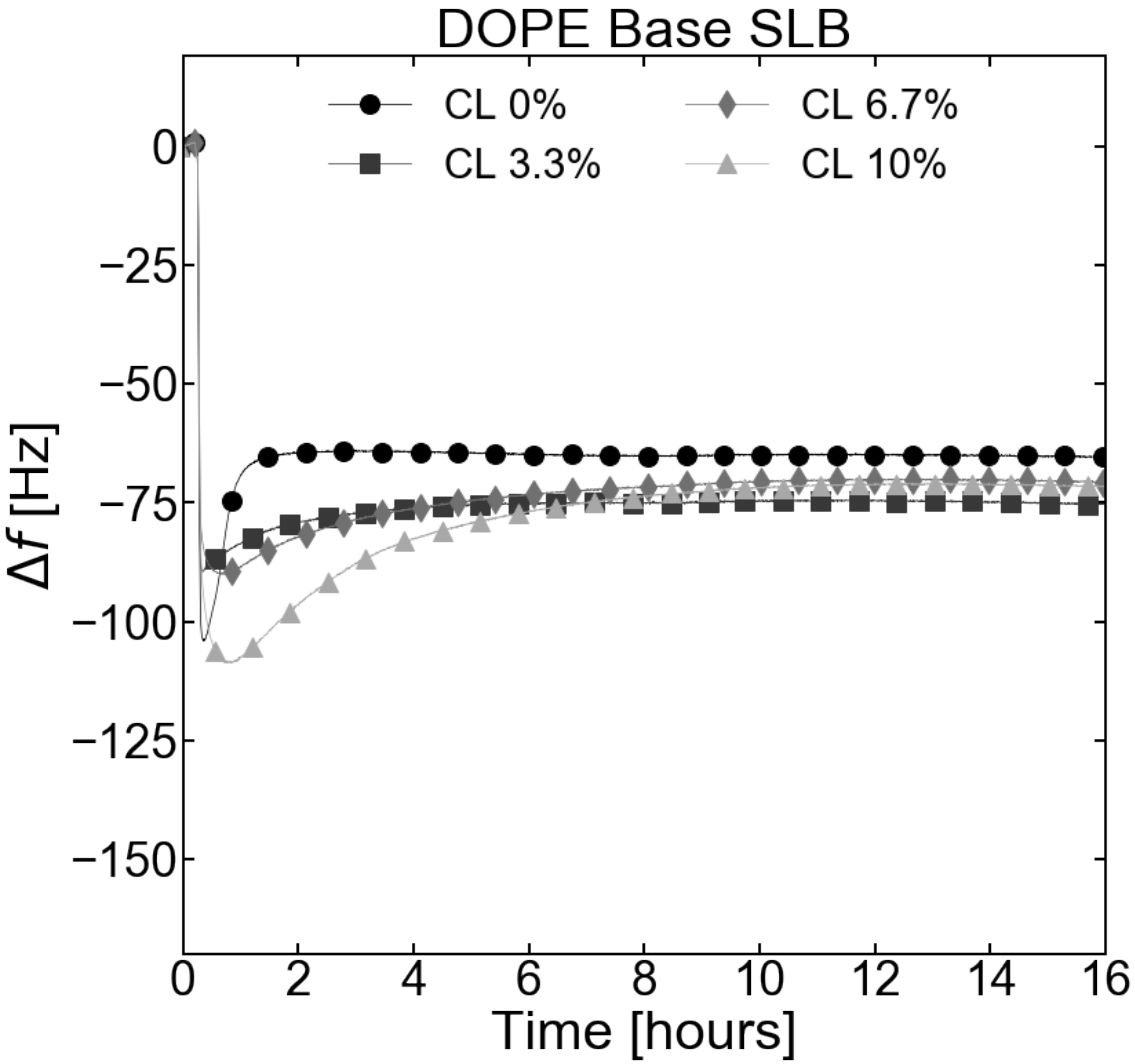

Figure S17. Adsorption kinetics of a blank TXTL reaction (P70a-*T7rnap*, 0.15 nM) onto 0, 3.3, 6.7, and 10% CL into a DOPE SLBs.

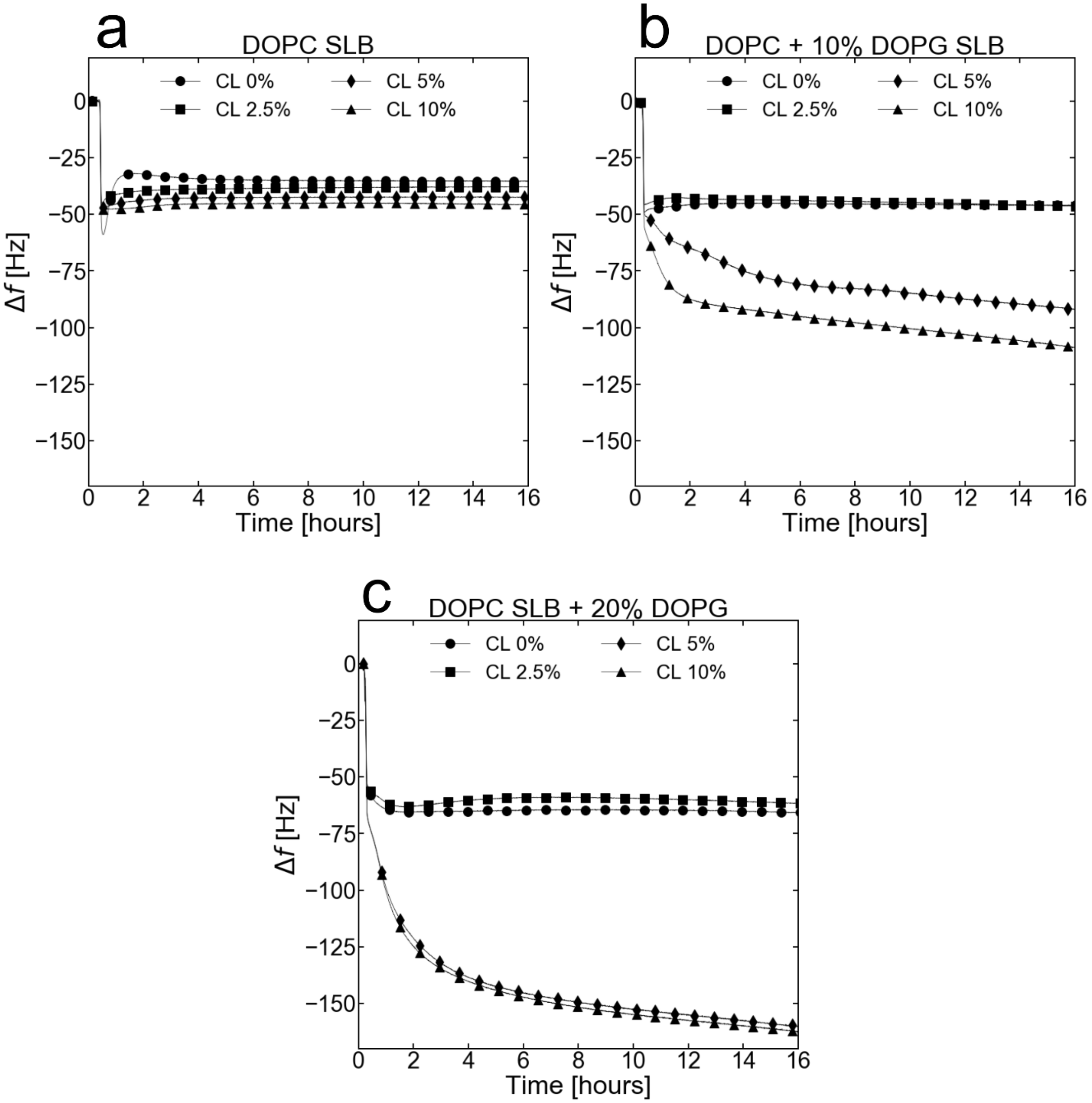

Figure S18. (a), (b), and (c) Adsorption kinetics of a blank TXTL reaction (P70a-*T7rnap*, 0.15 nM) onto a DOPC Base SLB as for different relative CL concentrations for a 0, 10, and 20% DOPG/DOPC mol. Ratio SLBs respectively.

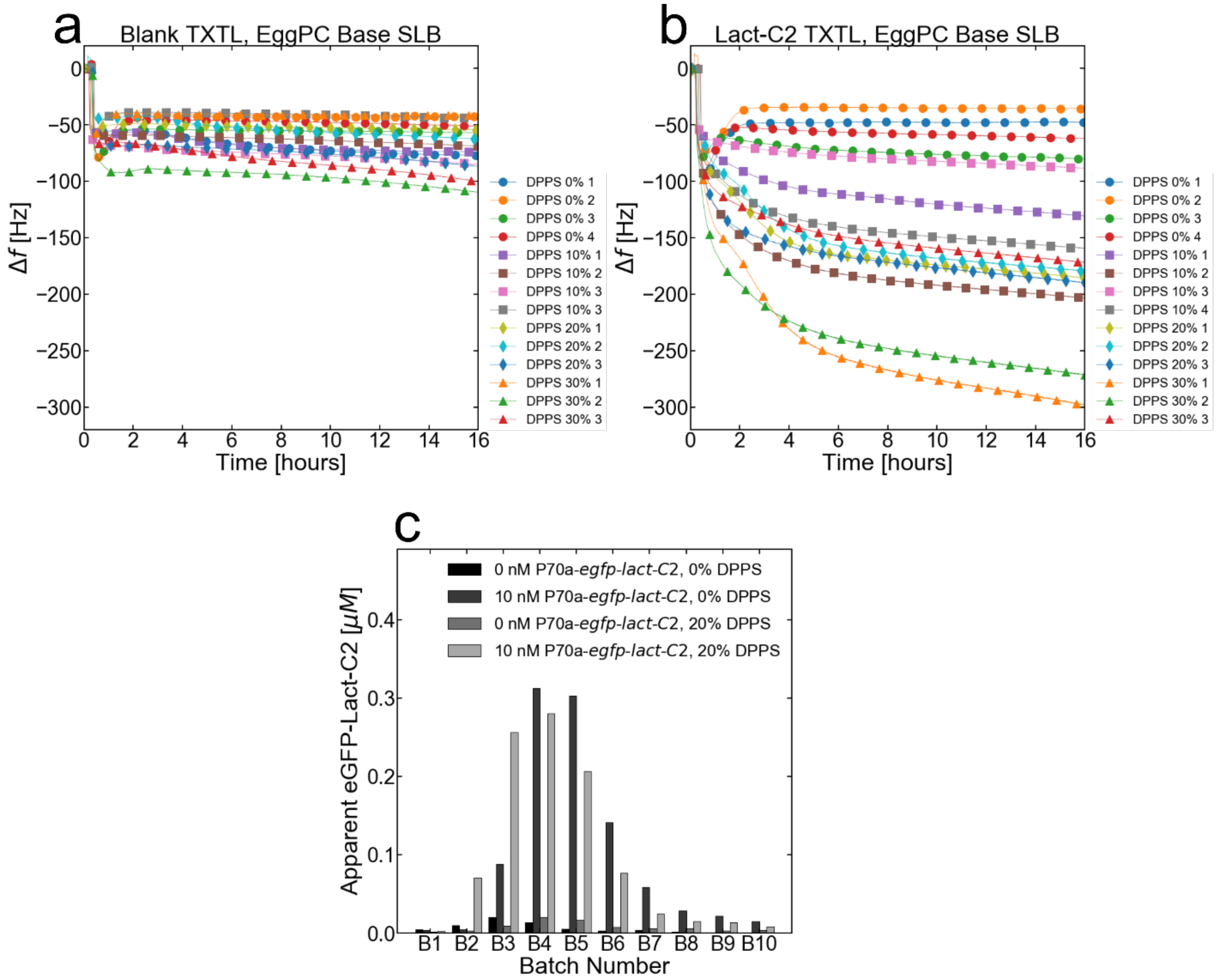

Figure S19. (a) and **(b)** Adsorption kinetics of either a blank (no DNA) and eGFP-Lact-C2 (P70a-*egfp-lact-C2*) TXTL reactions respectively incubated onto either a pure EggPC SLB or an EggPC SLB mixed with DPPS. **(c)** End-point measurement of TXTL expression with and without 20% DPPS in an EggPC SLB.

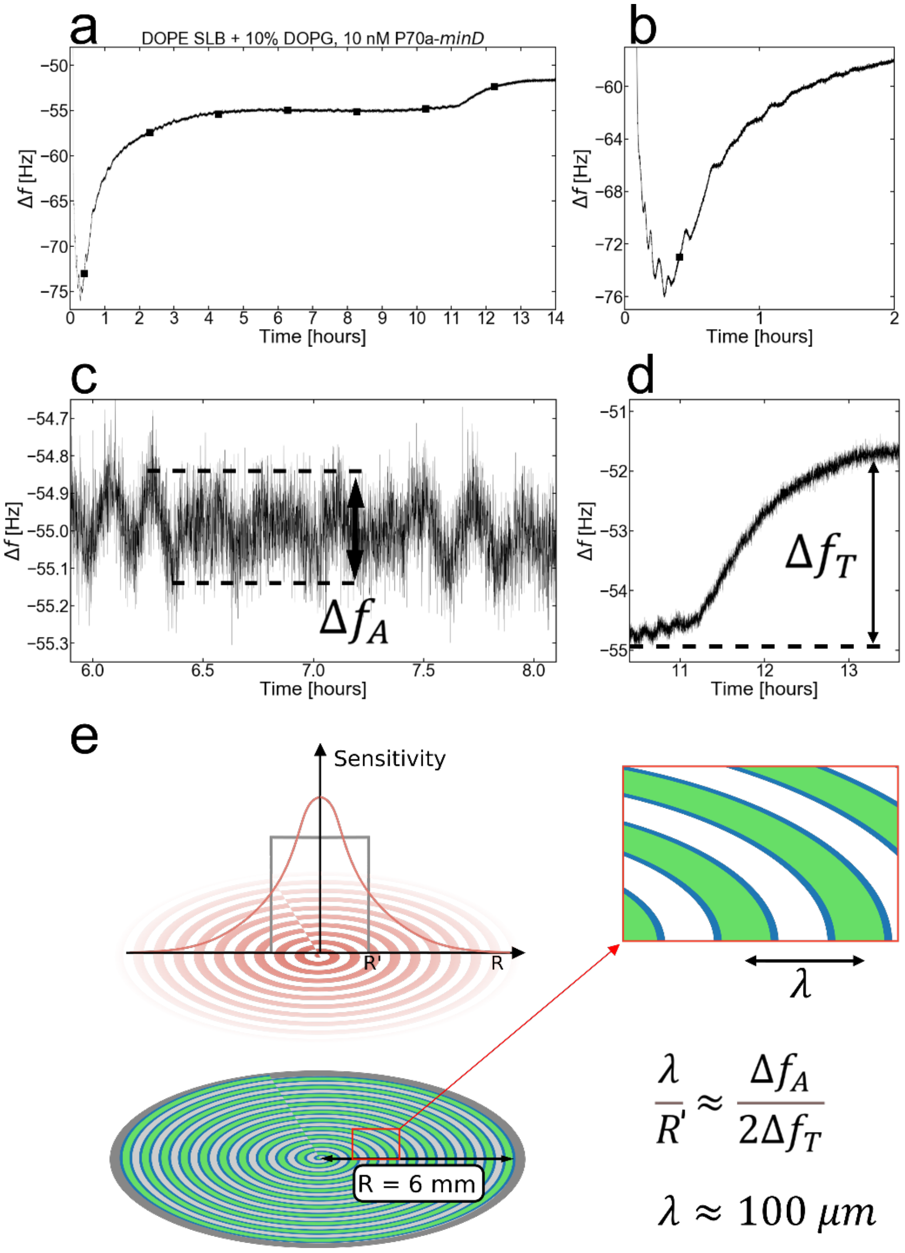

Fig. S20. (a) Adsorption kinetics of a MinD TXTL (P70a-*minD*, 10 nM) reaction incubated onto DOPE + 10% DOPG SLB. **(b)** Large amplitude oscillations during the first 2 hours of incubation. **(c)** Small-amplitude oscillations during the middle of the incubation. The amplitude of a single oscillation is labeled as Δf_A. **(d)** The increase in frequency at the end of the reaction due to ATP depletion is labeled as Δf_T. **(e)** Assuming that the Min patterns are radially symmetric fronts moving from the center of the sensor-SLB system to the edge, then Δf_A is proportional to the mass of the outermost ring escaping the boundaries of the most sensitive area of the sensor (grey box, $r < R' \approx R/3)$ [2], while Δf_T is proportional to the mass of all rings combined within the most sensitive area of the sensor. Since the escaping ring is twice the mass of the average ring within the box, then we can approximate the wavelength as in the equation in the panel. This approximation yields a wavelength of approximately 100 μm from the parameters obtained in **(c)** and **(d)**.

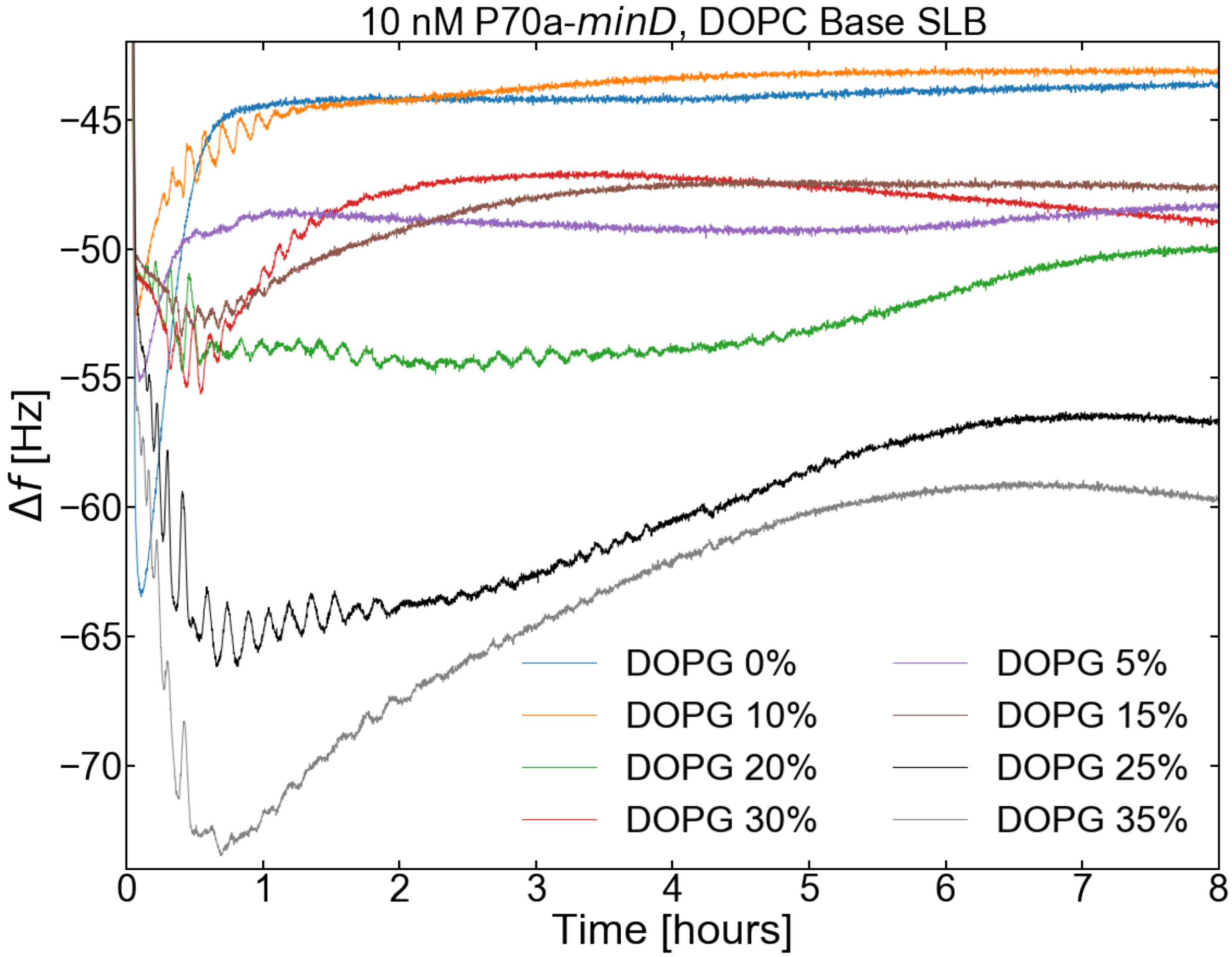

Figure S21. Adsorption kinetics of a MinD TXTL (P70a-*minD*, 10 nM) reaction for a range of different DOPG/DOPC composed SLBs.

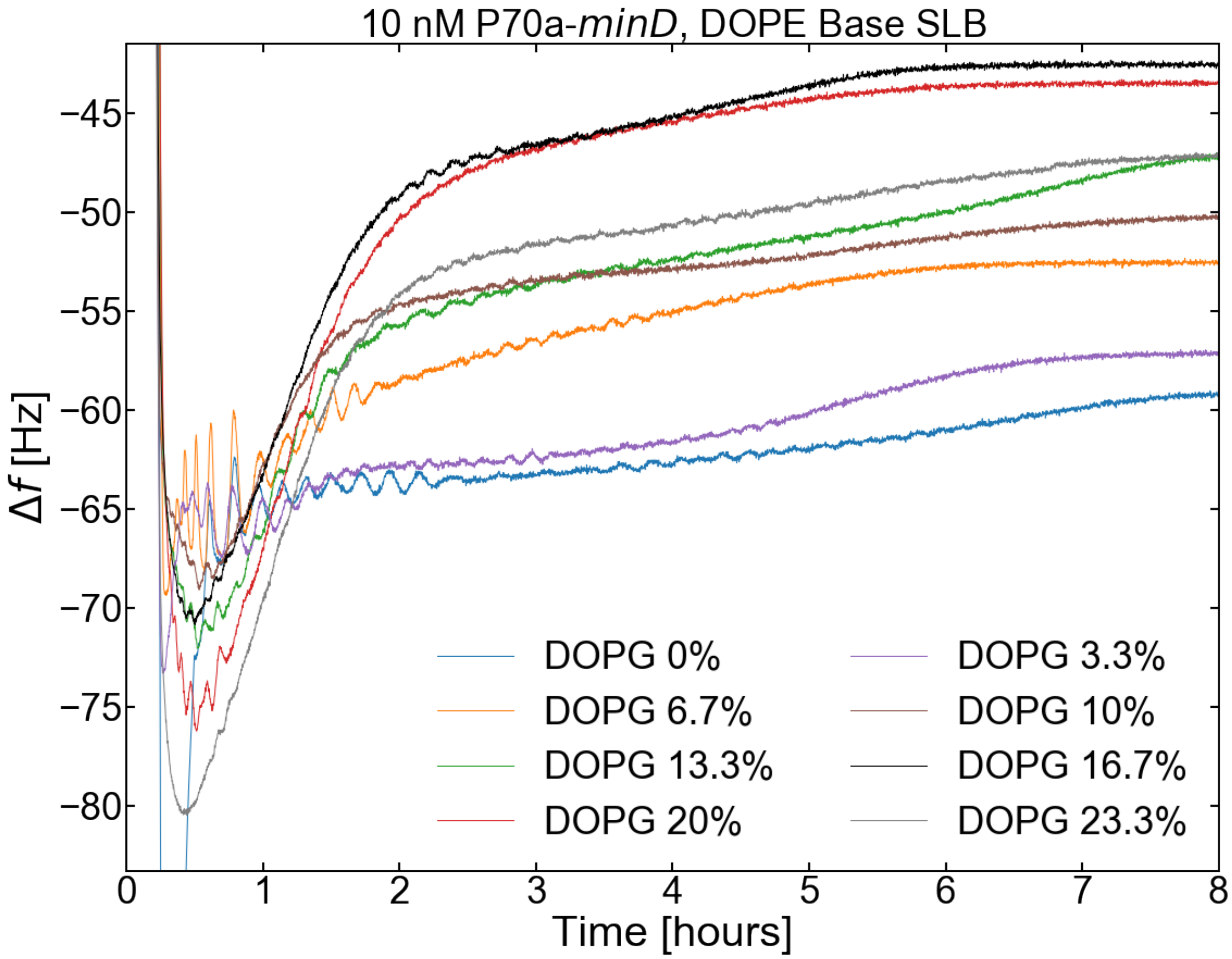

Figure S22. (a) Adsorption kinetics of a MinD TXTL (P70a-*minD*, 10 nM) reaction for a range of different DOPG/DOPE composed SLBs.

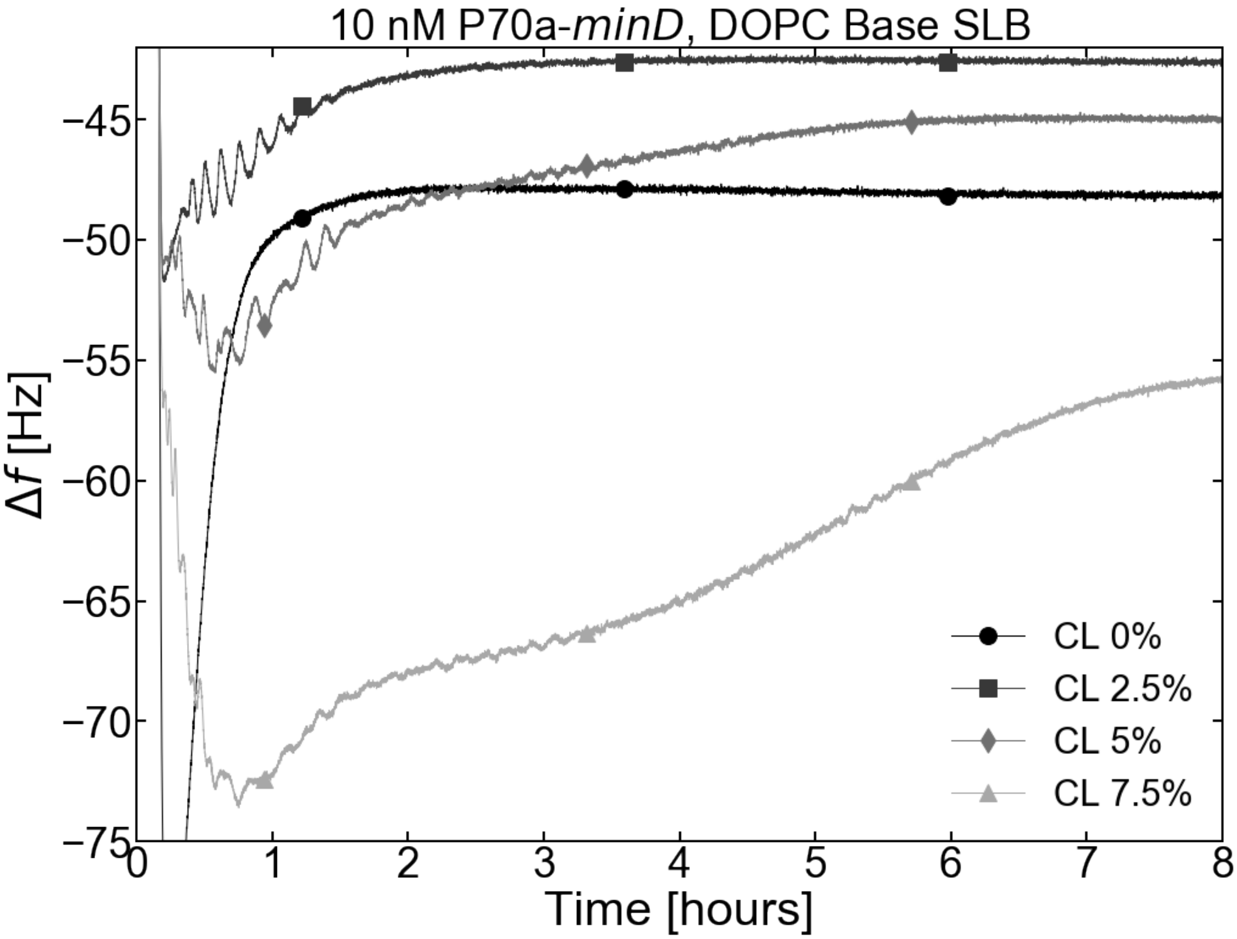

Figure S23. Adsorption kinetics of a MinD TXTL (P70a-*minD*, 10 nM) reaction for a range of different CL/DOPC composed SLBs.

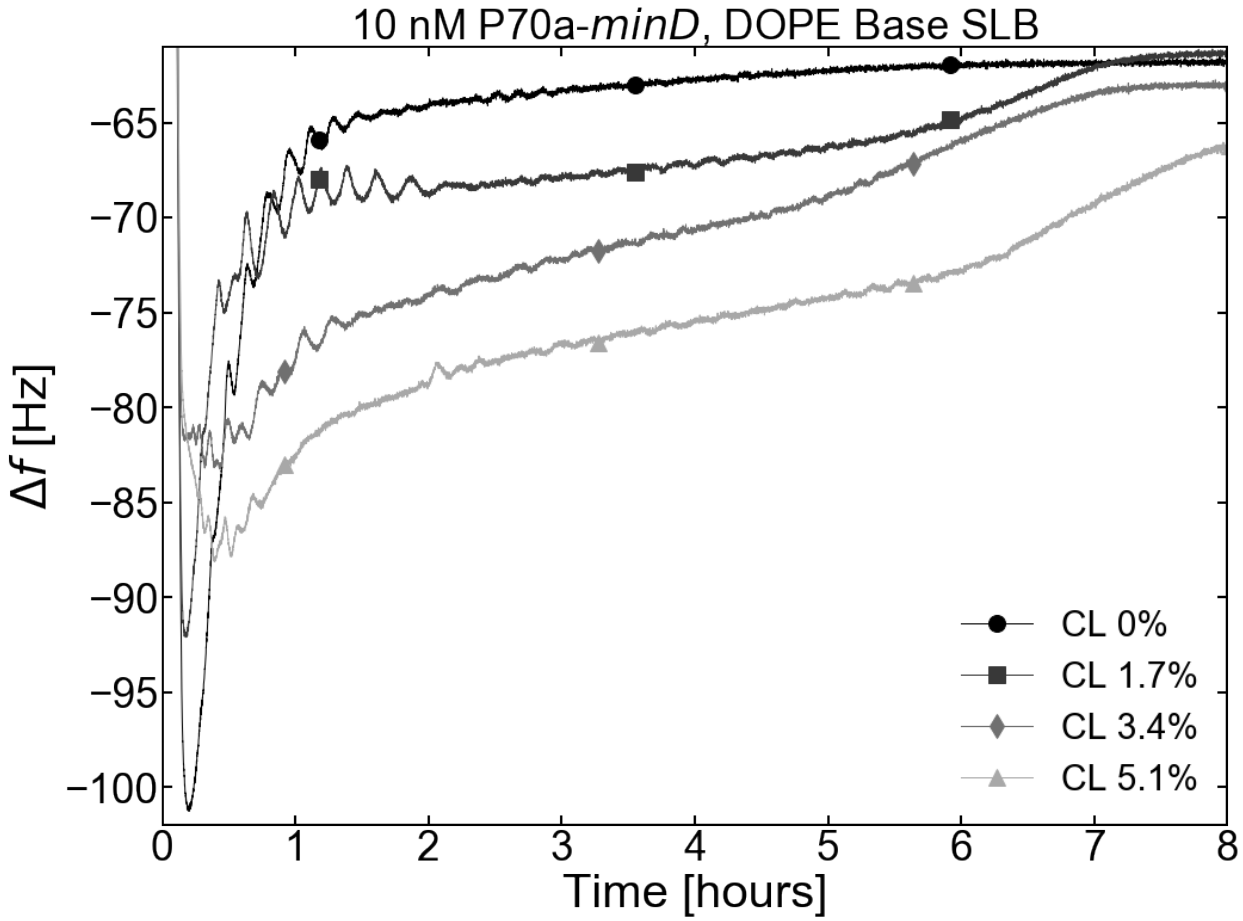

Figure S24. Adsorption kinetics of a MinD TXTL reaction (P70a-*minD*, 10 nM) for a range of different CL/DOPE composed SLBs.

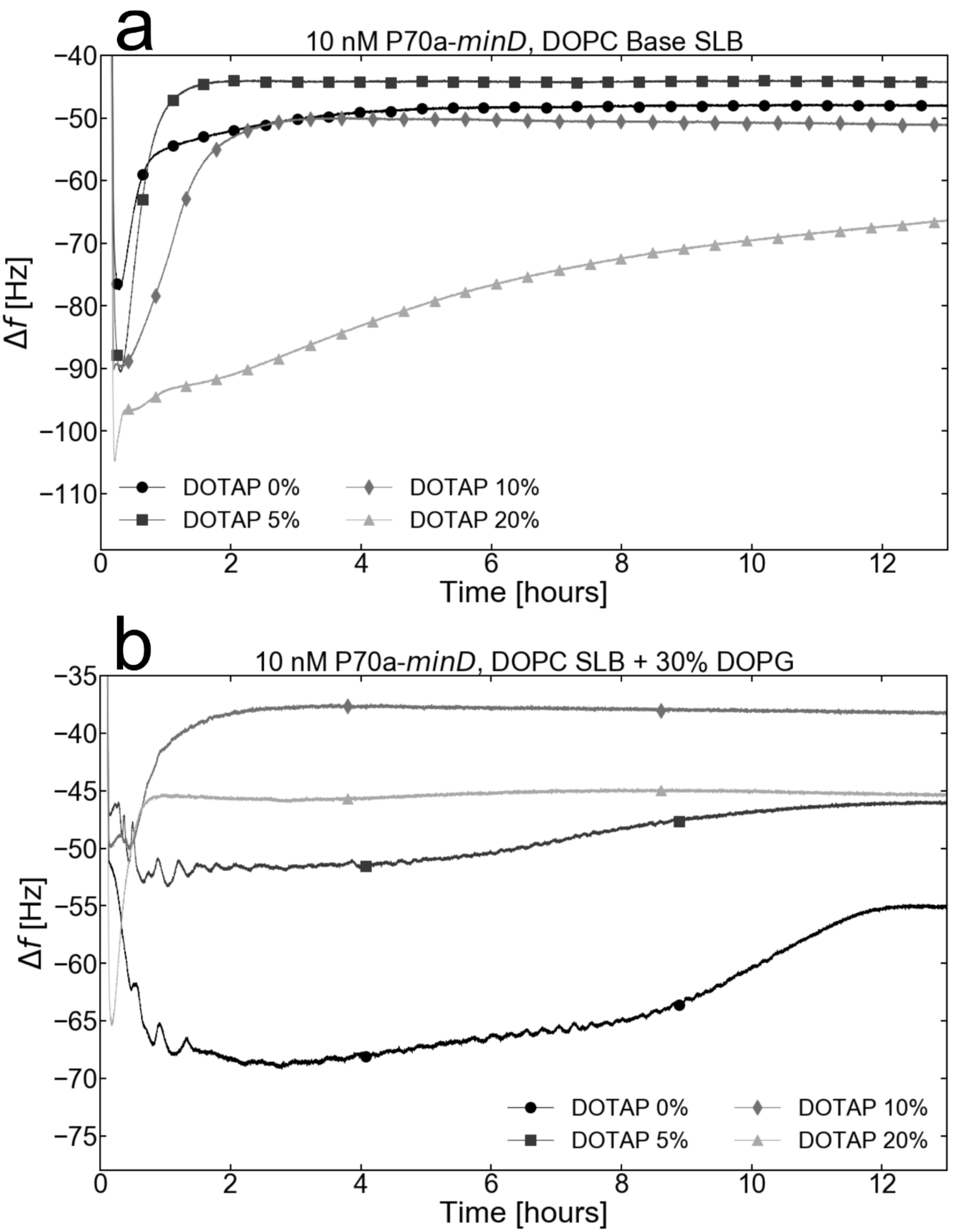

Figure S25. (a) and (b) Adsorption kinetics of a MinD TXTL (P70a-*minD*, 10 nM) reaction for a range of different DOTAP SALB concentrations for a pure DOPC SLB and a DOPG/DOPC SLB respectively.

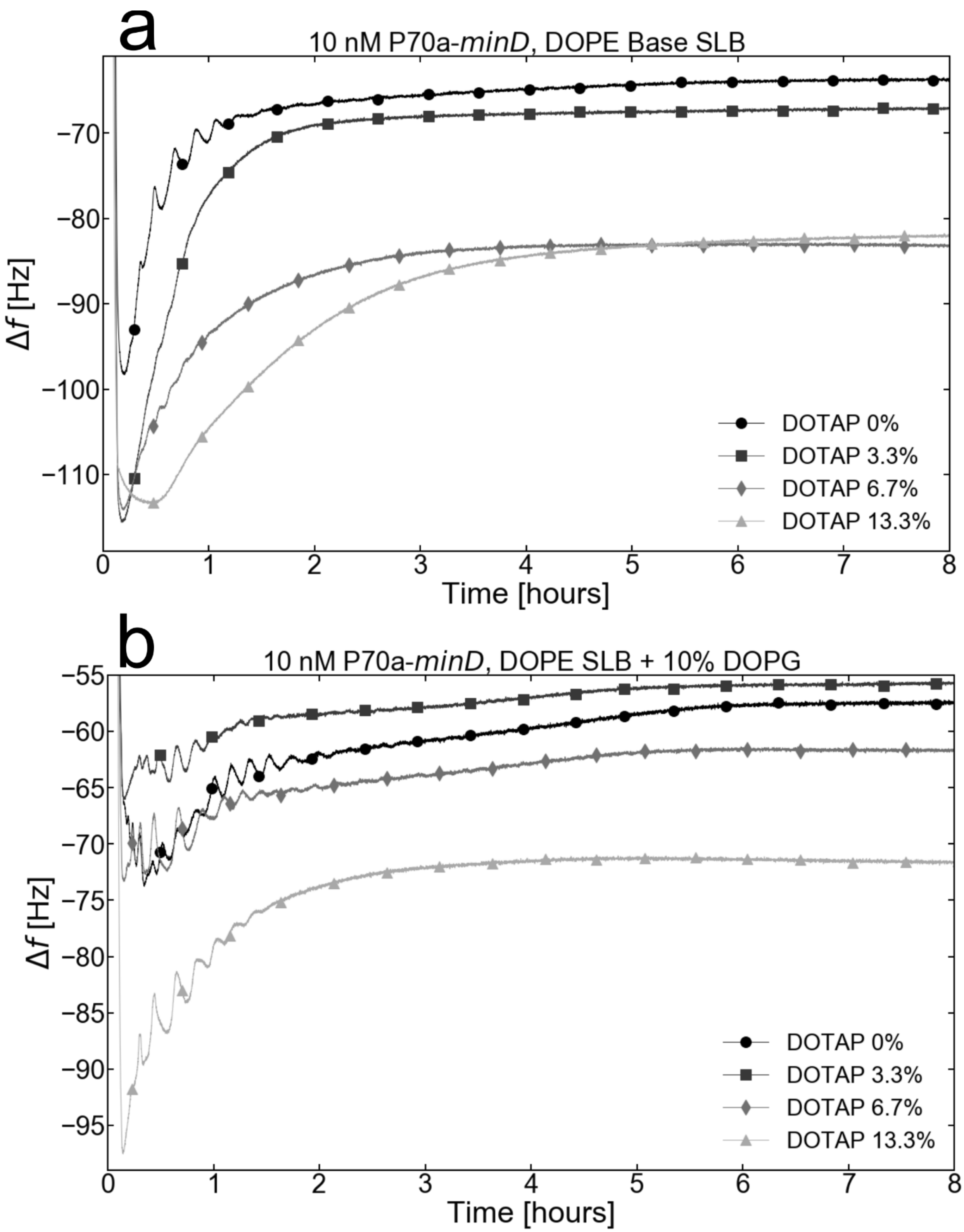

Figure S26. (a) and (b) Adsorption kinetics of a MinD TXTL (P70a-*minD*, 10 nM) reaction for a range of different DOTAP SALB concentrations for a pure DOPE SLB and a DOPG/DOPE SLB respectively.

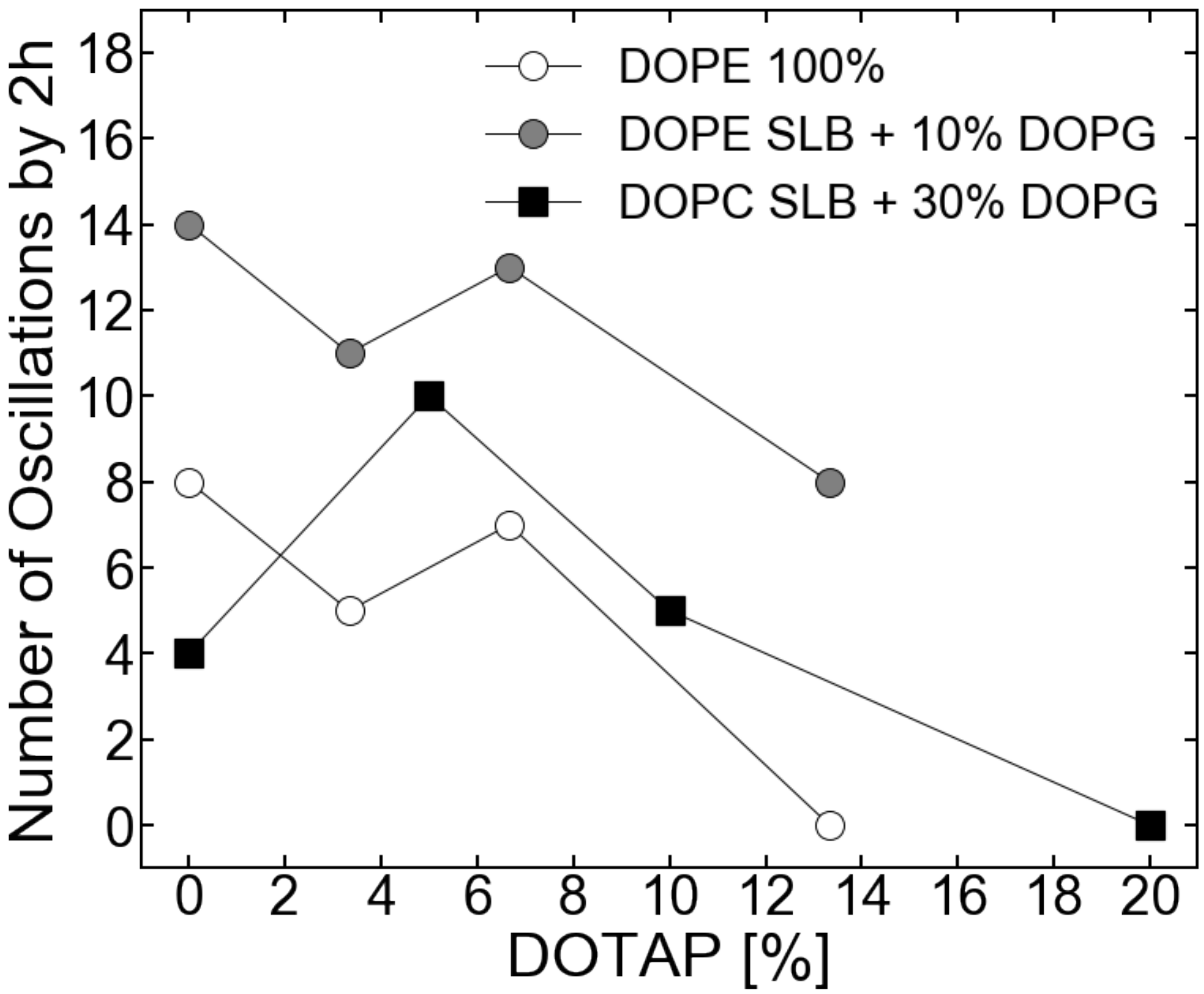

Figure S27. The number of oscillation peaks within the first 2 hours as a function of the relative DOTAP concentration during SALB for either a DOPE SLB, a DOPG/DOPE SLB, or a DOPG/DOPC SLB (P70a-*minD*, 10 nM).

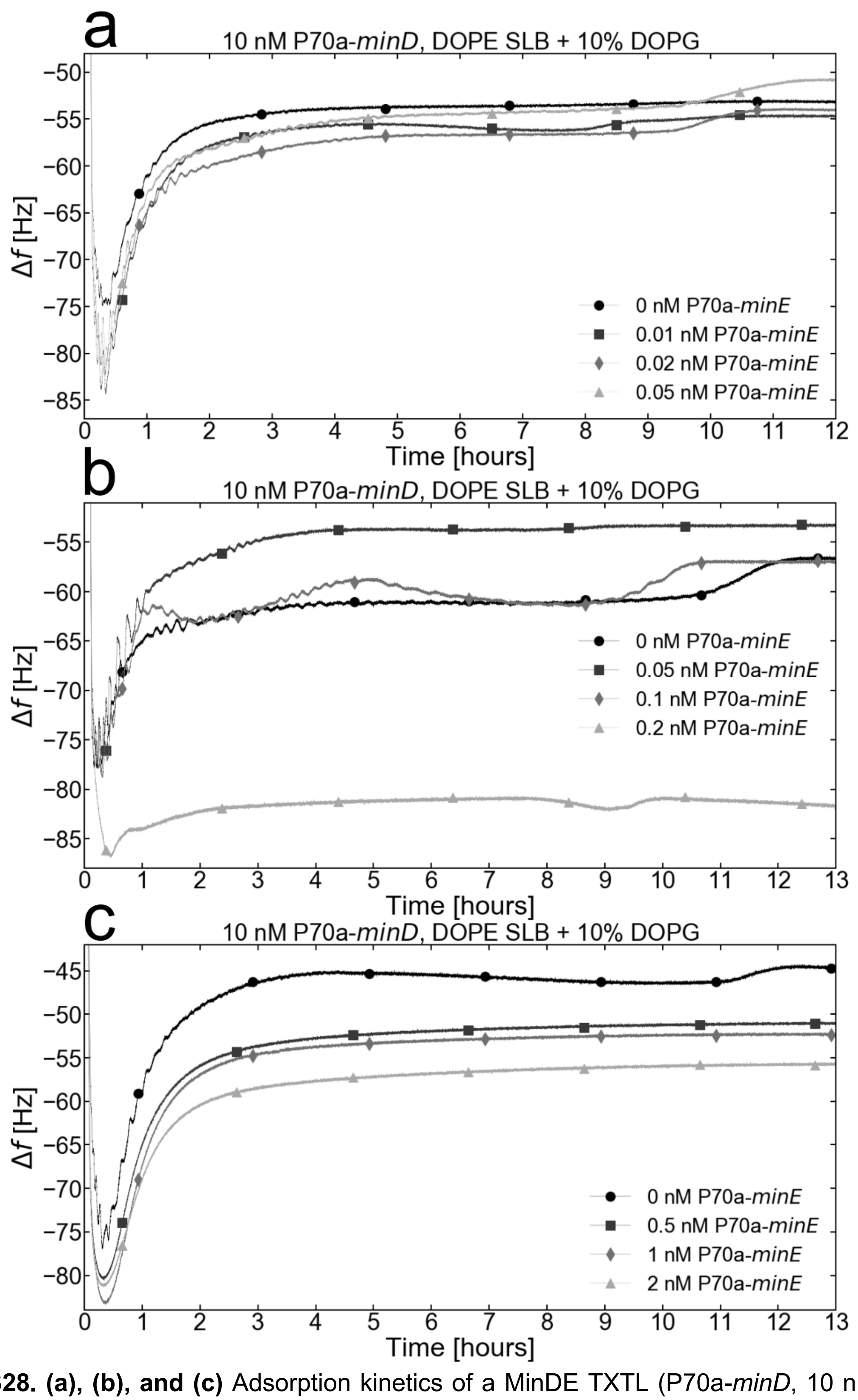

Figure S28. (a), (b), and (c) Adsorption kinetics of a MinDE TXTL (P70a-*minD*, 10 nM, P70a-*minE*, varied) reaction for different P70a-*minE* concentrations into a DOPG/DOPE 10% mol. Ratio SLB.

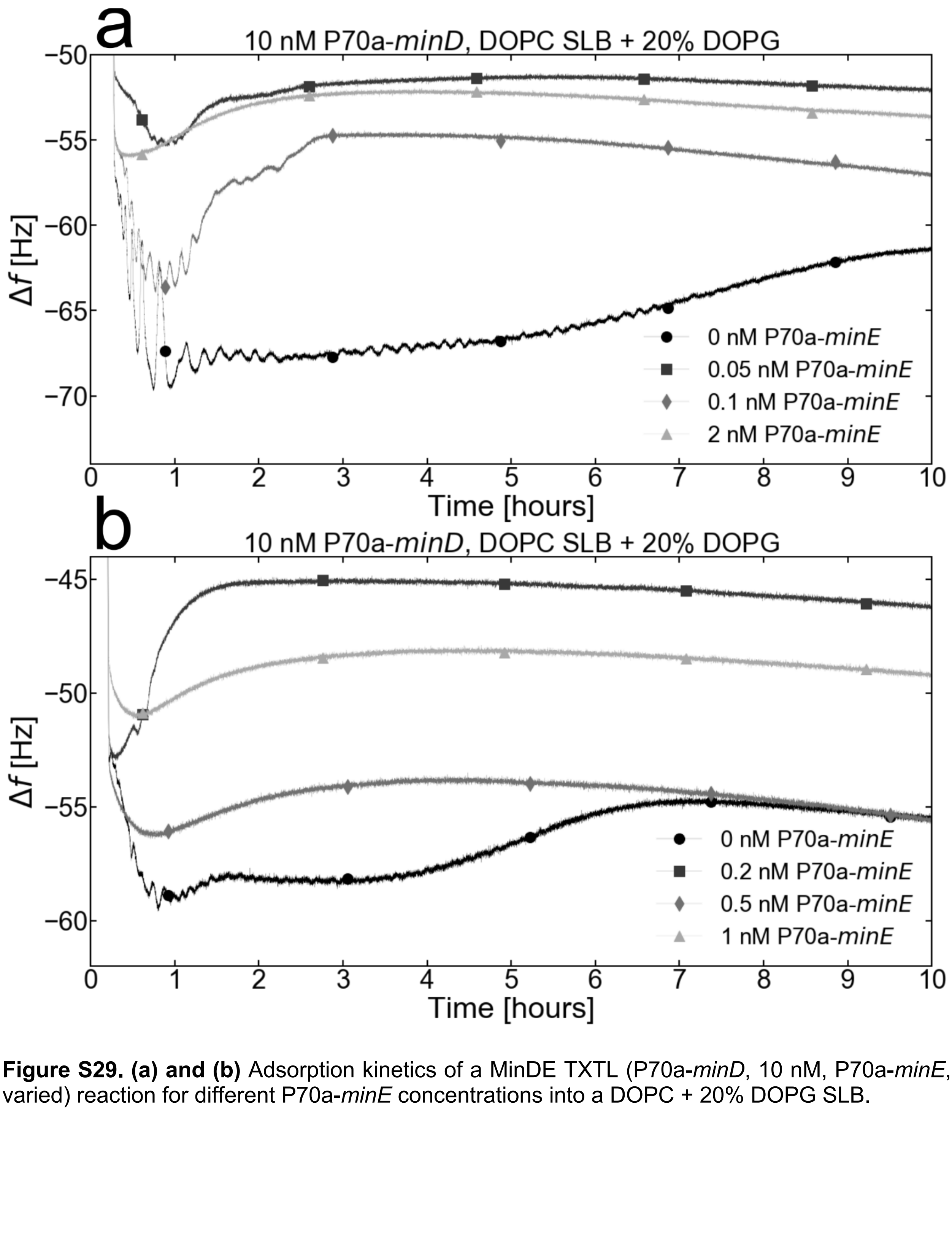

Figure S29. (a) and (b) Adsorption kinetics of a MinDE TXTL (P70a-*minD*, 10 nM, P70a-*minE*, varied) reaction for different P70a-*minE* concentrations into a DOPC + 20% DOPG SLB.

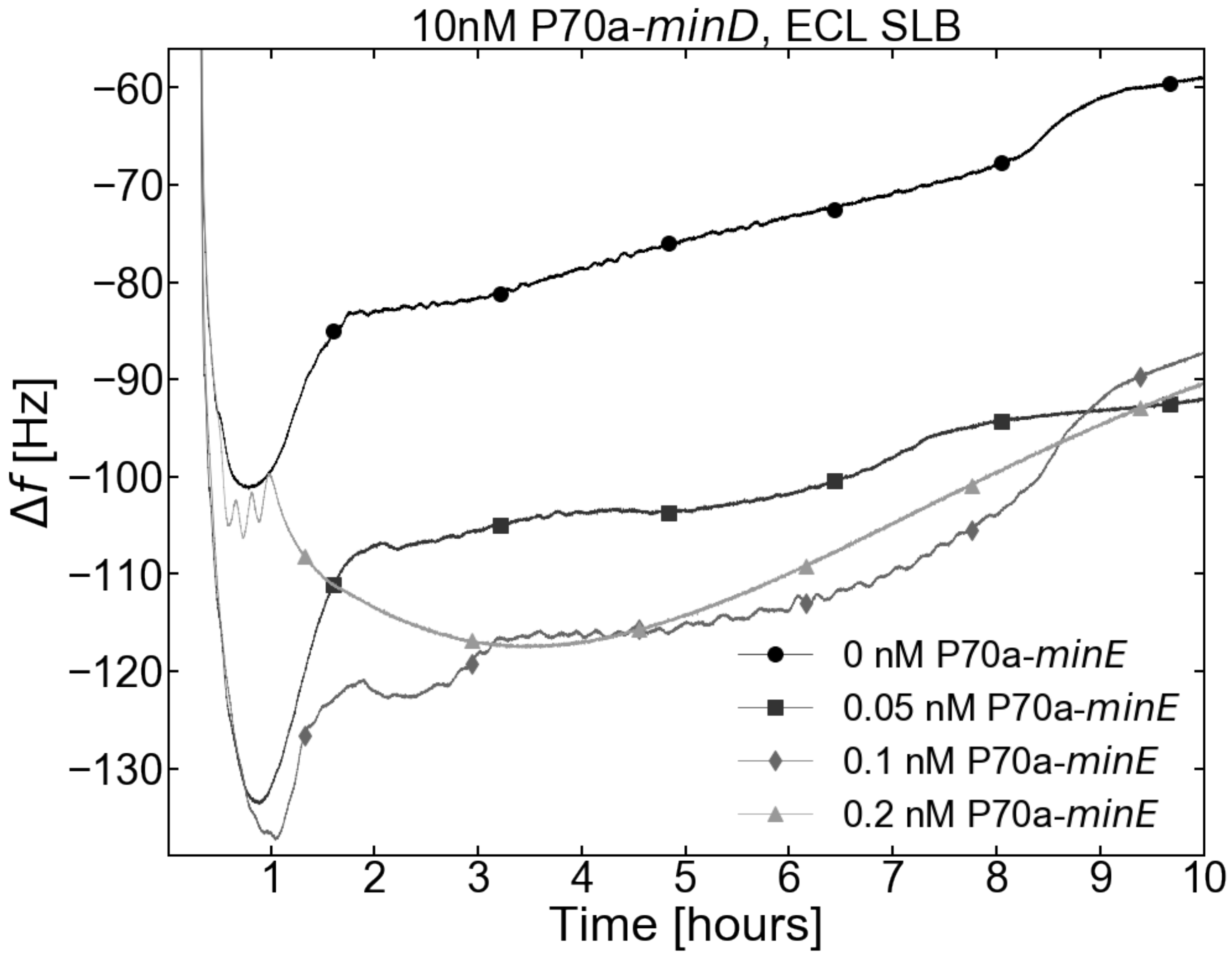

Figure S30. Adsorption kinetics of a MinDE TXTL (P70a-*minD*, 10 nM, P70a-*minE*, varied) reaction for different P70a-*minE* concentrations into a ECL SLB.

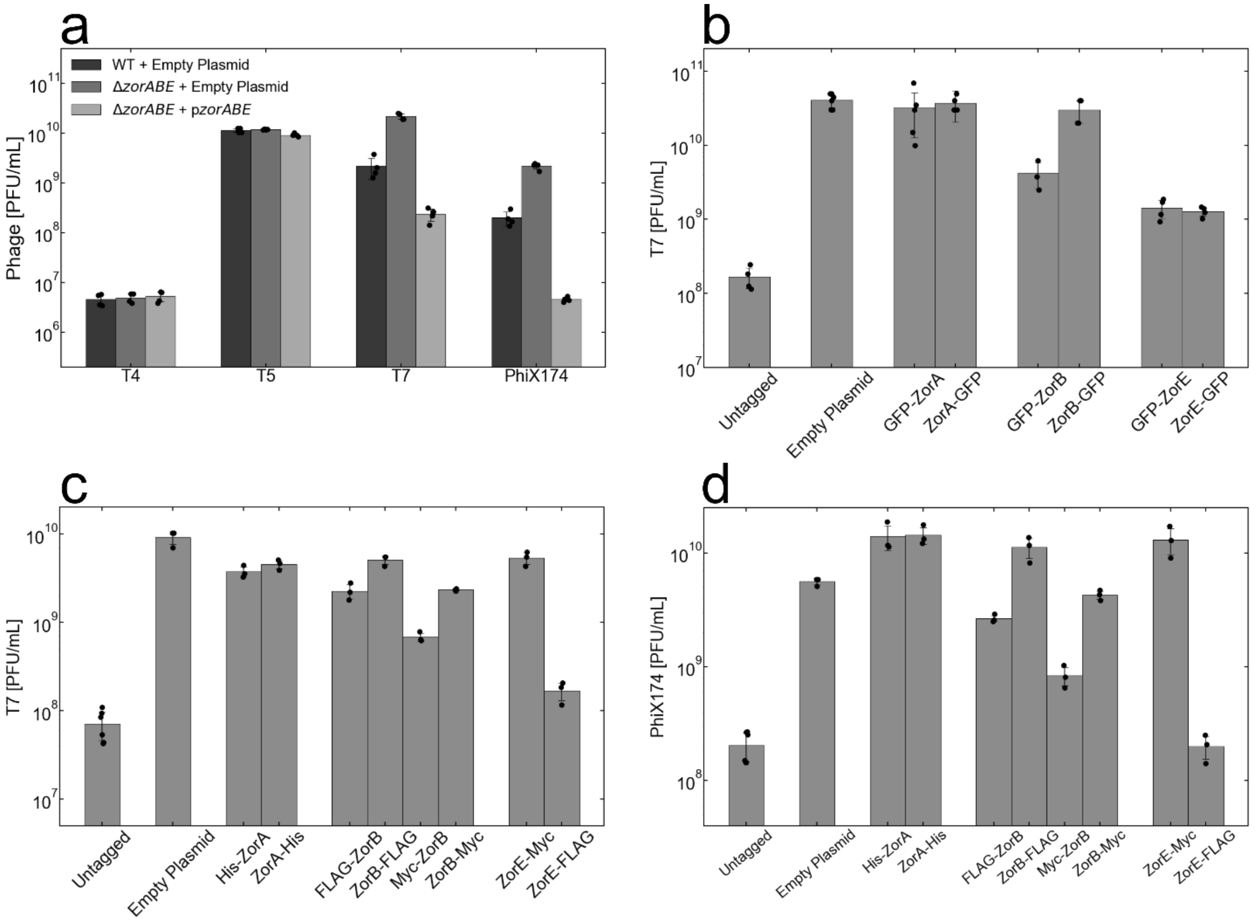

Figure S31. Tagging of Zorya proteins disrupts defense against phages. (a) Reduction in plaque forming units in the presence of Zorya. **(b)** Reduction in T7 phage plaque forming units in the Δ*zorABE* knockout mutant. Zorya was supplemented on plasmids containing different combinations of the GFP tag at the termini of the respective proteins. **(c)** Reduction in the T7 plaque forming units in the Δ*zorABE* knockout mutant. Zorya was supplemented on plasmids containing different combinations of His, Myc, and FLAG tags at the termini of the respective proteins. **(d)** Reduction in the phiX174 plaque forming units in the Δ*zorABE* knockout mutant. Zorya was supplemented on plasmids containing different combinations of His, Myc, and FLAG tags at the termini of the respective proteins. The value and the uncertainty of the bars are the mean and standard deviation of at at least 3 replicates.

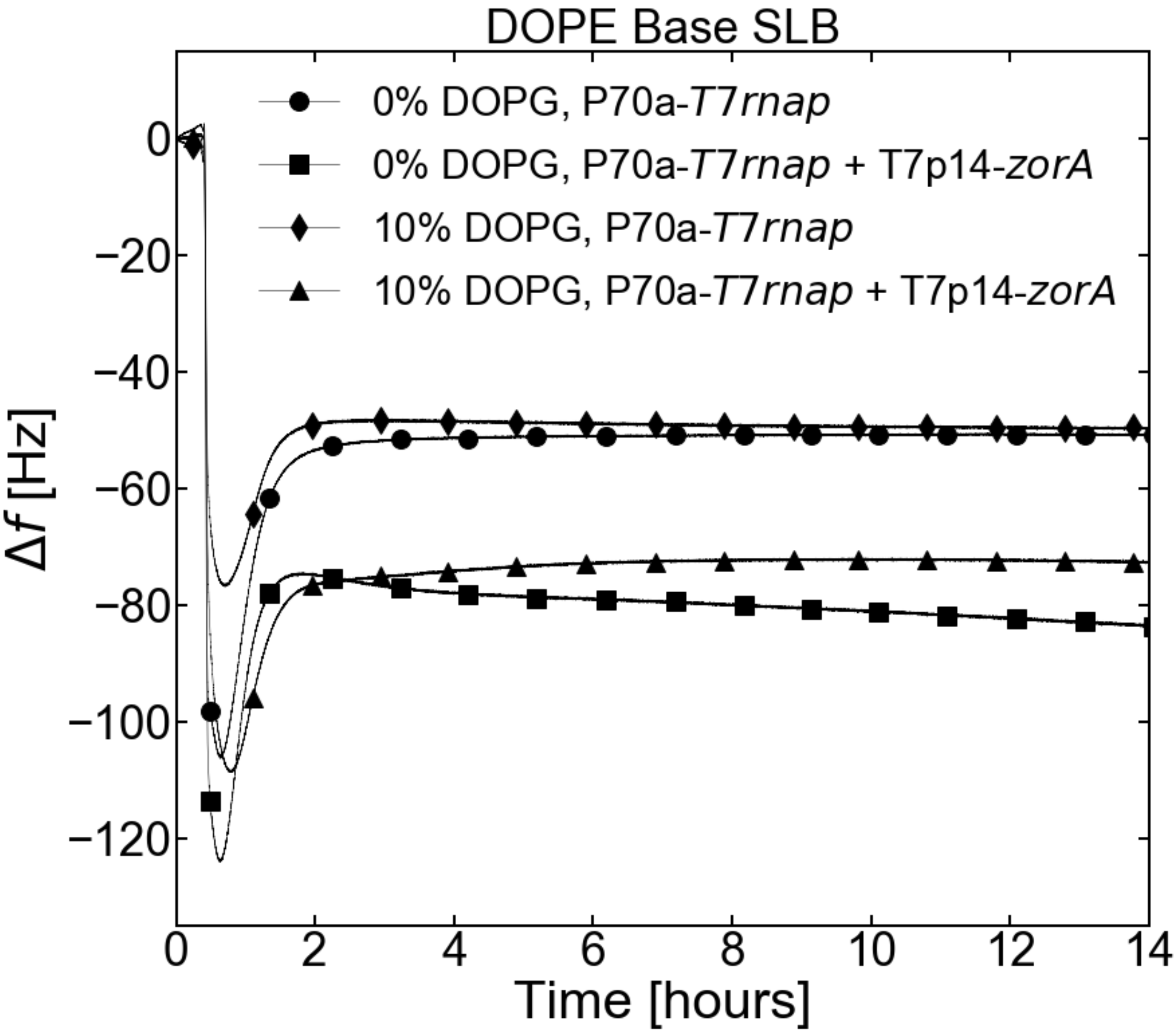

Figure S32. The adsorption kinetics of the control (P70a-*T7rnap*, 0.2 nM) and ZorA (P70a-*T7rnap*, 0.2 nM, T7p14-*zorA*, 10 nM) conditions with either a pure DOPE or DOPE + 10% DOPG SLBs.

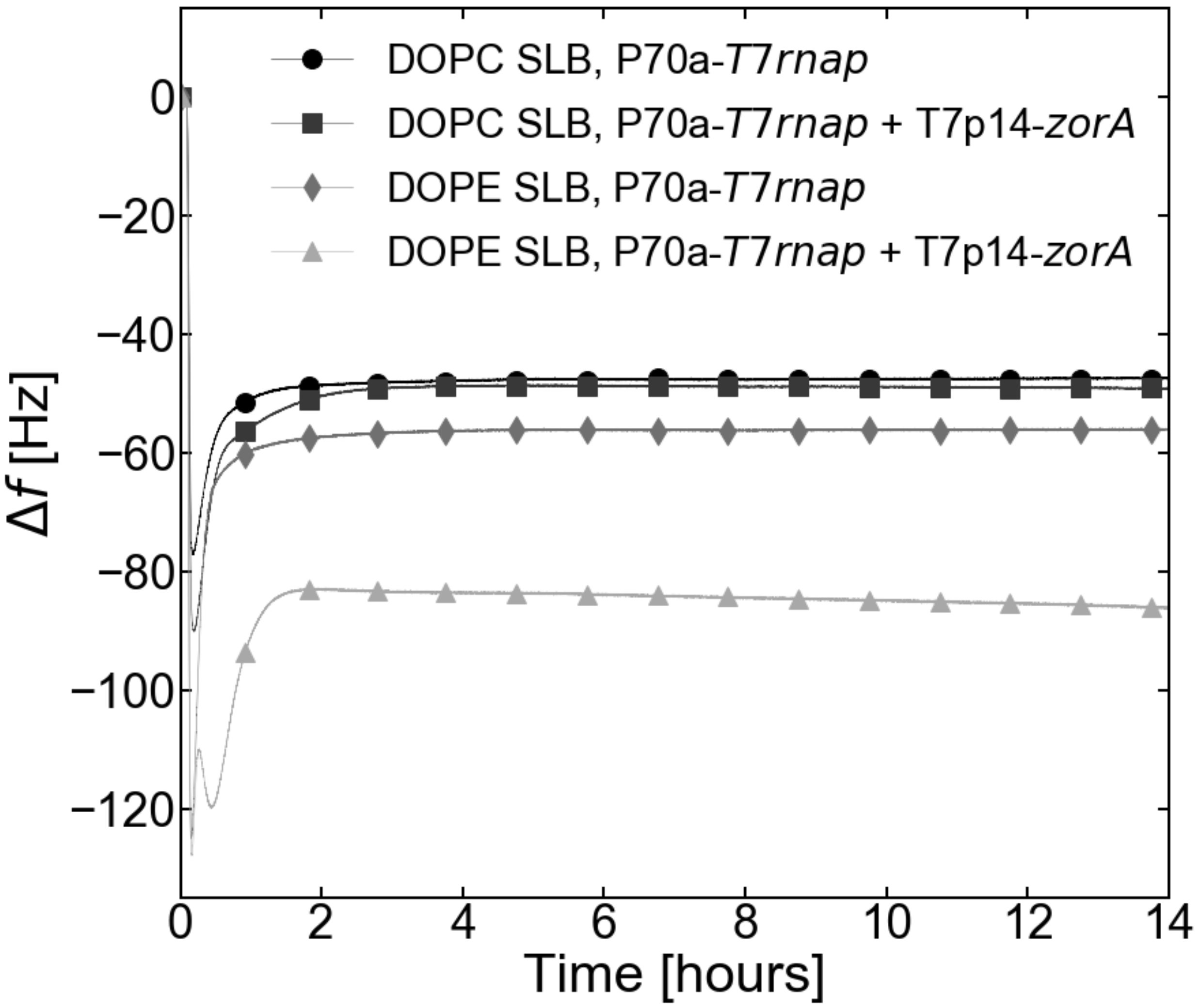

Figure S33. The adsorption kinetics of the control (P70a-*T7rnap*, 0.2 nM) and ZorA (P70a-*T7rnap*, 0.2 nM, T7p14-*zorA*, 10 nM) conditions with either a pure DOPC or pure DOPE SLBs.

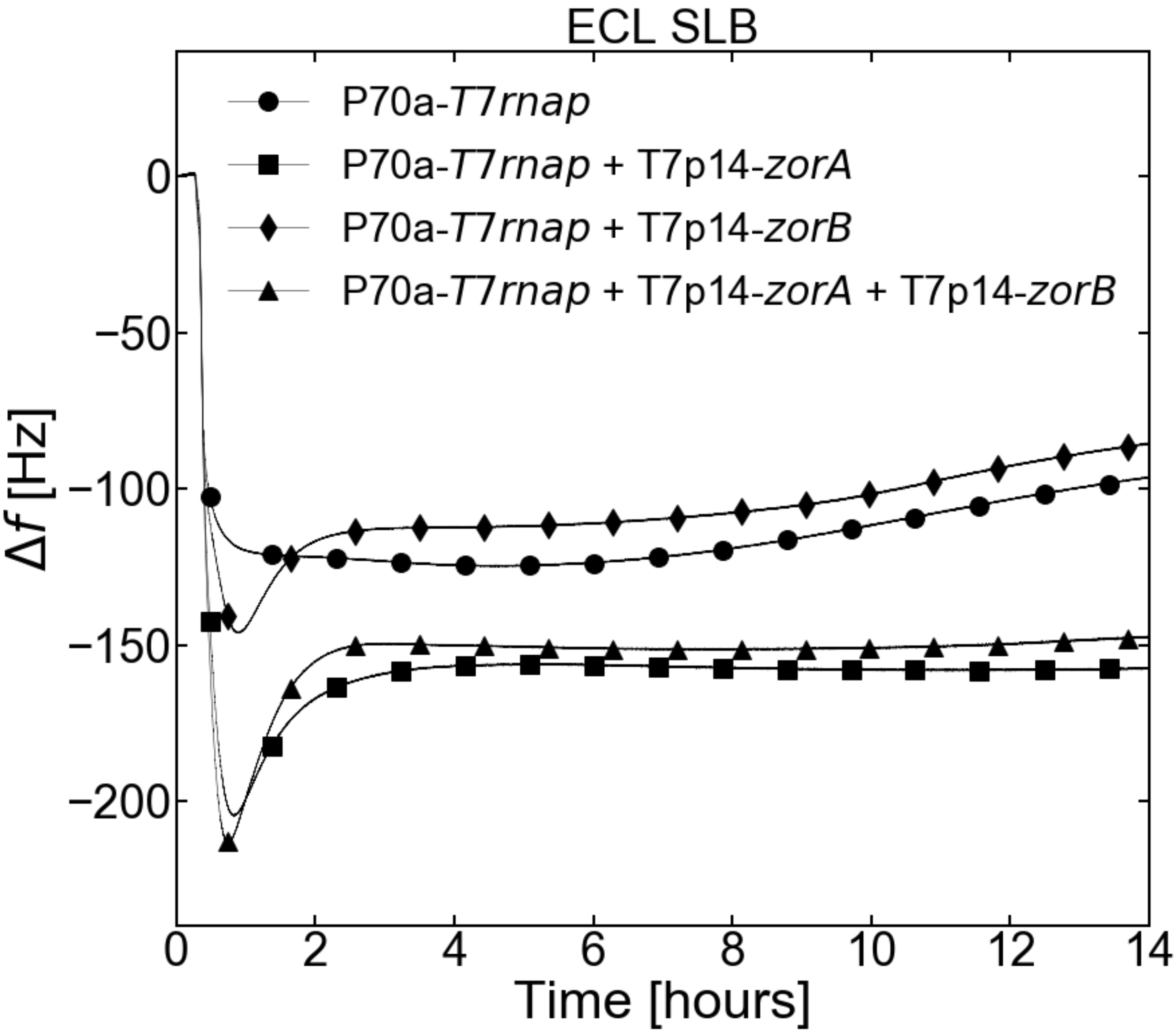

Figure S34. The adsorption kinetics of the control (P70a-*T7rnap*, 0.2 nM), ZorA alone (P70a-*T7rnap*, 0.2 nM, T7p14-*zorA*, 10 nM), ZorB alone (P70a-*T7rnap*, 0.2 nM, T7p14-*zorB*, 10 nM), and ZorA and ZorB together (P70a-*T7rnap*, 0.2 nM, T7p14-*zorA*, 10 nM, and T7p14-*zorB*, 10 nM) with an ECL SLB.

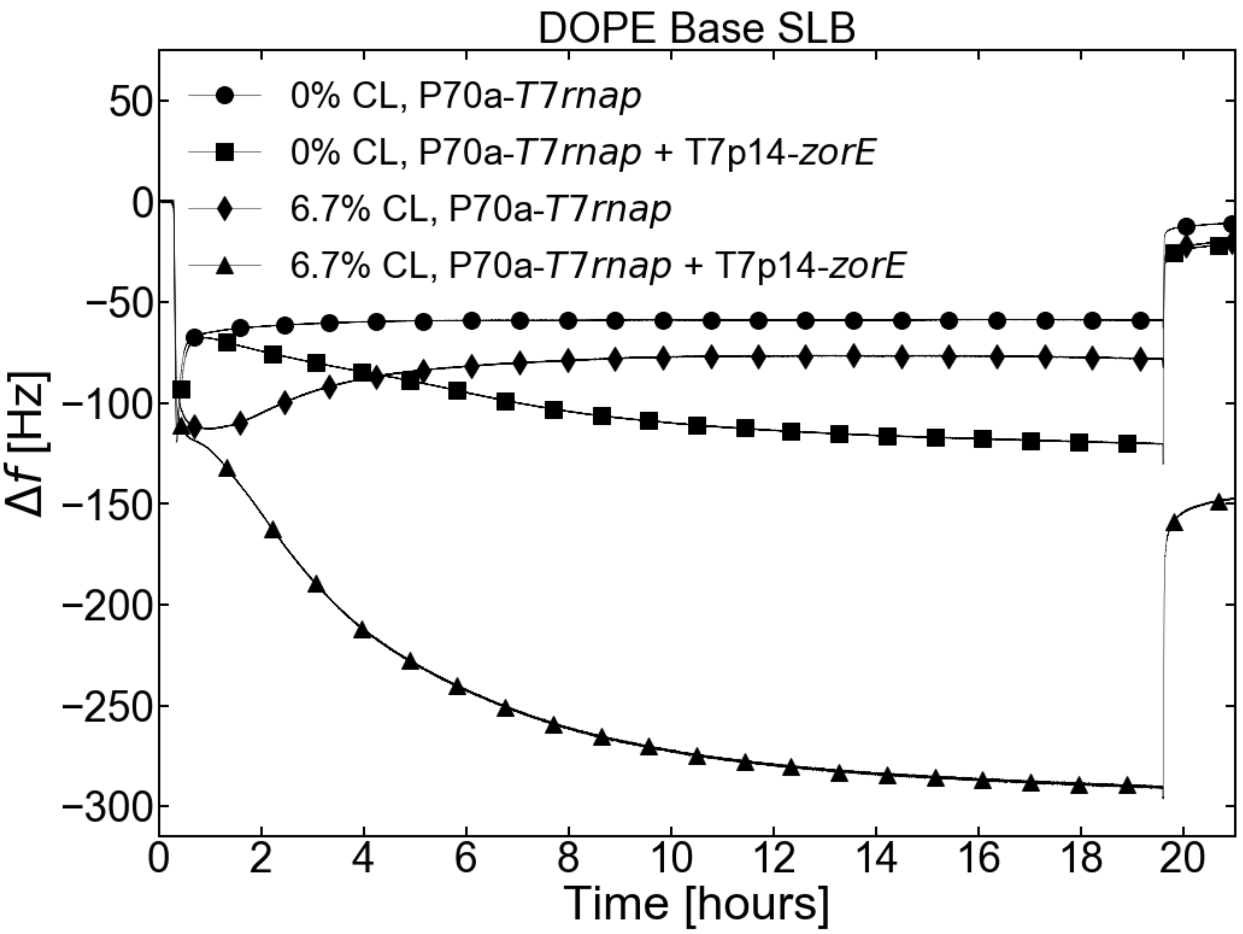

Figure S35. Same as in **Fig. 6f** but with the measurement extended to t = 21 h after TXTL incubation start. The 1-h Tris NaCl flush starts at t = 19.6 h.

SLB composition	concentration (mM)	mass density (mg/mL)
DOPC	1.3 mM	1 mg/mL
EggPC	1.3 mM	1 mg/mL
DOPE	1.95 mM	1.5 mg/mL
E. coli lipids (ECL)	3 mM (approximated)	3 mg/mL

Supplementary Table S1. Concentrations of phospholipids in IPA during SALB formation to obtain full coverage of the QCMD sensor and no nonspecific TXTL adsorption. Note that ECL are a mix of several phospholipids of different molecular weights, the average molecular weight of which is not communicated by the manufacturer but roughly estimated from its approximate composition.

References

1. Hamill, O. P. & Martinac, B. Molecular Basis of Mechanotransduction in Living Cells. *Physiological Reviews* **81**, 685–740 (2001).
2. Hillier, A. C. & Ward, M. D. Scanning electrochemical mass sensitivity mapping of the quartz crystal microbalance in liquid media. *Anal. Chem.* **64**, 2539–2554 (1992).
