# Supplementary material for: Cell-free expression with a quartz crystal microbalance enables rapid, dynamic, and label-free characterization of membrane-interacting proteins
Source: Commun Biol. 2024 Aug 17;7:1005. doi: 10.1038/s42003-024-06690-9 (PMC11329788; doi:10.1038/s42003-024-06690-9)
Supplement: Supplementary file 2 — Description of Additional Supplementary File [file 42003_2024_6690_MOESM2_ESM.pdf]

Description of Additional Supplementary Files

File name: Supplementary Data 1

Description: Source data behind the graphs in the paper.

File name: Supplementary Data 2

Description: Plasmids and strains used in this study.
