# Supplementary material for: Cell-free expression with a quartz crystal microbalance enables rapid, dynamic, and label-free characterization of membrane-interacting proteins
Source: Commun Biol. 2024 Aug 17;7:1005. doi: 10.1038/s42003-024-06690-9 (PMC11329788; doi:10.1038/s42003-024-06690-9)
Supplement: Supplementary file 4 — Supplementary Data 2 [file 42003_2024_6690_MOESM4_ESM.pdf]

List of plasmids and linear DNA used in this study

DNA Name	Plasmid or Linear	ID	Benchling Link
P70a-deGFP (Plasmid)	Plasmid	No ID	https://benchling.com/s/seq-1gNUXg0A3lBVf556eAXw?m=slm-nMLpZCFR9Gaw7jLqSTsF
P70a-T7RNAp (Plasmid)	Plasmid	No ID	https://benchling.com/s/seq-rAhPBqYvIG7zTaUGC2iF?m=slm-iIQuca6mgC1I85vv3D8A
T7p14-deGFP (Plasmid)	Plasmid	No ID	https://benchling.com/s/seq-JhiUQwAQhJCodnSWSo4A?m=slm-Y8hrFrHrg12N4PLGE2qk
T7p14-MscL (Plasmid)	Plasmid	No ID	https://benchling.com/s/seq-Q1of0ufkrvJfn1kLdYB2?m=slm-NcNrIxmDvjehtIlYv2LC
T7p14-aHL-eGFP (Plasmid)	Plasmid	No ID	https://benchling.com/s/seq-ReWms9Pv6eWuCcDW9WtT?m=slm-1hAwFn7qVoLruklk6YYT
P70a-eGFP-LactC2 (Plasmid)	Plasmid	No ID	https://benchling.com/s/seq-5wgQIAMPP7oiaOfZr6qk?m=slm-JMV9zcV5njYsKTYI982A
P70a-MinD	Linear	No ID	https://benchling.com/s/seq-P9tMTfEpKqf6zn7YympR?m=slm-O5uV66h40py0DxHZ5F8U
P70a-MinE	Linear	No ID	https://benchling.com/s/seq-Bz5QSKvYUqgU26dei5k3?m=slm-WdxE1xtsglR211boklX2
P70a-MinC	Linear	No ID	https://benchling.com/s/seq-kdLu9i9uStCEJ265sE4u?m=slm-b973qHb6Xp7AJiFCEAPR
Zorya (*zorABE* from DSM-1576 in pSG (ATCC 8739)	Plasmid	**CBS-5891**	https://benchling.com/s/seq-GCoULHjWslwgEWmsCDBe?m=slm-jxQrbhIy8EdBAKv3Vh67
pKD13 Lambda Red recombineering plasmid	Plasmid	**CBS-217**	https://benchling.com/s/seq-zm5YCVYWj72qS0IVCTQn?m=slm-ahBh68usU5X1VbIjo8sf
pCP20 Lambda Red recombineering plasmid	Plasmid	**CBS-218**	https://benchling.com/s/seq-6w98X5CyYnlhT6z34kFA?m=slm-krmpkl1Tla5BXvhg1XoH
pKD46 Lambda Red recombineering plasmid	Plasmid	**CBS-219**	https://benchling.com/s/seq-T9kbgiwOTDCA9HAicsqy?m=slm-WdI0GZoV0zLoFYnGXUv5
Zorya DSM-157 ZorA N-GFP (Plasmid	Plasmid	**OD-686**	https://benchling.com/s/seq-bURXCcWOY4Iiay1iwjRd?m=slm-jAHBw3IPJR5oJFQKH8uI

Zorya DSM-157 ZorA C-GFP	Plasmid	**OD-687**	https://benchling.com/s/seq-kKwQHzJUnTVb5Z8EwrS2?m=slm-fcsBBrISu5JitFLe6F0L
Zorya DSM-157 ZorB N-GFP	Plasmid	**OD-688**	https://benchling.com/s/seq-v0e5fwPsgA8dt0LTL562?m=slm-fU7VZv5f6n6mxmstV8fn
Zorya DSM-157 ZorB C-GFP	Plasmid	**OD-689**	https://benchling.com/s/seq-SDZ5c7BitFo0kMom510F?m=slm-u7qNneSHfs57WyMczC6B
Zorya DSM-157 ZorE N-GFP	Plasmid	**OD-690**	https://benchling.com/s/seq-jLAHw9noBGbwGmykbplo?m=slm-opaXyzohUfoESu2kyO3J
Zorya DSM-157 ZorE C-GFP	Plasmid	**OD-691**	https://benchling.com/s/seq-gyw4KShLp2LVTvbLpnWs?m=slm-UEcOStbQVtpLHwJrCNHX
Zorya DSM-1576 ZorA N-His	Plasmid	**OD-953**	https://benchling.com/s/seq-IbkQmDq30FKQanEpfQMA?m=slm-Bq3VDPsaDj4r61J8IUIl
Zorya DSM-1576 ZorB N-FLAG	Plasmid	**OD-954**	https://benchling.com/s/seq-dW2baJobidD79Pl9CNC5?m=slm-yNx0obSuMvLQBqI3iMix
Zorya DSM-1576 ZorB C-Myc	Plasmid	**OD-955**	https://benchling.com/s/seq-vMoGy3mKXItlcSeWkpVU?m=slm-nHrWaXgWf9zTixfknwh9
Zorya DSM-1576 ZorE-C3xFLAG	Plasmid	**OD-956**	https://benchling.com/s/seq-m6Nl8R35Dfww62Y4AqP9?m=slm-DOuCOFIm4z7SmchLul9J
Zorya DSM-1576 ZorB N-Myc	Plasmid	**OD-957**	https://benchling.com/s/seq-GiZzWdpQN8j55YhFmtSS?m=slm-UfQGUbJwobMlU95mlu6A
Zorya DSM-1576 ZorA C-His	Plasmid	**OD-958**	https://benchling.com/s/seq-g593EN35xSTcMCrvPjCm?m=slm-3ru0xBxzH4hbUyLe8eKs
Zorya DSM-1576 ZorE C-Myc	Plasmid	**OD-959**	https://benchling.com/s/seq-mCuifTZkK9dei82Ql6tj?m=slm-Mj5RUkQ24G866gD1x4ft
Zorya DSM-1576 ZorB C-FLAG	Plasmid	**OD-960**	https://benchling.com/s/seq-BqdggFDaNLTu3Dn0U4FH?m=slm-8bIN0PgiY7OmiYPz7J8V
T7p14-ZorA	Linear	No ID	https://benchling.com/s/seq-kucNipM65t3OUAgZZ4Tg?m=slm-b5YPUZ3pilxPcOle5AoO
T7p14-ZorB	Linear	No ID	https://benchling.com/s/seq-Fi8GIsp5XOyezW6tdU0s?m=slm-o87Mrr7ausTbZpkMeGEq
T7p14-ZorE	Linear	No ID	https://benchling.com/s/seq-oxj65rw6pujF98N2QqSj?m=slm-mLMh0to7dfCn90v5tMB1

List of strains and phages used in this work.

ID	Strain	Descritption	Source
**CBS-521**	*Escherichia coli* DSM1576 (ATCC 8739)		DSMZ
**OD-675**	*Escherichia coli* DSM1576 (ATCC 8739)	Δ*zorABE* (4,265,744 -> 4,269,528)	This work
	Enterobacteria phage T4	DSM No.: 4505	DSMZ
	Enterobacteria phage T5	DSM No.: 16353	DSMZ
	Enterobacteria phage T7	DSM No.: 4623	DSMZ
	Enterobacteria phage phiX174	DSM No.: 4497	DSMZ
